# Genomic-based taxonomic classification of the family *Erythrobacteraceae*

**DOI:** 10.1099/ijsem.0.004293

**Published:** 2020-07-29

**Authors:** Lin Xu, Cong Sun, Chen Fang, Aharon Oren, Xue-Wei Xu

**Affiliations:** ^1^​ Key Laboratory of Marine Ecosystem Dynamics, Ministry of Natural Resources & Second Institute of Oceanography, Ministry of Natural Resources, Hangzhou 310012, PR China; ^2^​ College of Life Sciences and Medicine, Zhejiang Sci-Tech University, Hangzhou 310018, PR China; ^3^​ College of Oceanography, Hohai University, Nanjing 210000, PR China; ^4^​ The Institute of Life Sciences, The Hebrew University of Jerusalem, Edmond J. Safra Campus, Jerusalem 9190401, Israel; ^5^​ School of Oceanography, Shanghai Jiao Tong University, Shanghai 200030, PR China

**Keywords:** *Erythrobacteraceae*, phylogenomic reconstruction, AAI, evolutionary distance

## Abstract

The family *Erythrobacteraceae *, belonging to the order *Sphingomonadales *, class *Alphaproteobacteria *, is globally distributed in various environments. Currently, this family consist of seven genera: *Altererythrobacter *, *Croceibacterium *, *Croceicoccus *, *Erythrobacter *, *Erythromicrobium *, *Porphyrobacter* and *Qipengyuania *. As more species are identified, the taxonomic status of the family *Erythrobacteraceae* should be revised at the genomic level because of its polyphyletic nature evident from 16S rRNA gene sequence analysis. Phylogenomic reconstruction based on 288 single-copy orthologous clusters led to the identification of three separate clades. Pairwise comparisons of average nucleotide identity, average amino acid identity (AAI), percentage of conserved protein and evolutionary distance indicated that AAI and evolutionary distance had the highest correlation. Thresholds for genera boundaries were proposed as 70 % and 0.4 for AAI and evolutionary distance, respectively. Based on the phylo-genomic and genomic similarity analysis, the three clades were classified into 16 genera, including 11 novel ones, for which the names *Alteraurantiacibacter*, *Altericroceibacterium*, *Alteriqipengyuania*, *Alteripontixanthobacter*, *Aurantiacibacter*, *Paraurantiacibacter*, *Parerythrobacter*, *Parapontixanthobacter*, *Pelagerythrobacter*, *Tsuneonella* and *Pontixanthobacter* are proposed. We reclassified all species of *Erythromicrobium* and *Porphyrobacter* as species of *Erythrobacter *. This study is the first genomic-based study of the family *Erythrobacteraceae *, and will contribute to further insights into the evolution of this family.

## Introduction

The family *Erythrobacteraceae *, belonging to the order *Sphingomonadales *, class *Alphaproteobacteria* [1], is distributed globally, inhabiting various environments including subterrestrial, lake, intertidal areas, mangrove, coastal and deep-sea sediments [2–10], soil [11–13], desert sands [14, 15], a stadium seat [16], seawater [17–19], estuary water [20–22], fresh water [23, 24], hot springs [25–27], air [28] as well as plants and animals [29–36] (Table S1, available in the online version of this article). The members of the family *Erythrobacteraceae* are Gram-stain-negative, rod or pleomorphic coccoid-shaped, pink-, red-, orange- or yellow-pigmented, and aerobic chemoorganotrophs [1]. The majority require NaCl for growth [1]. Ubiquinone-10 (Q-10) is the major respiratory quinone [1, 2, 30].

The family was established by Lee *et al*. who included the genera *Erythrobacter* (*Erb. litoralis* and *Erb. longus*), *Erythromicrobium* (*Erm. ramosum*) and *Porphyrobacter* (* Por. neustonensis* and *Por. tepidarius *) based on 16S rRNA gene phylogeny in 2005 [37]. Four other genera including *Altererythrobacter* (*Aeb*.), *Croceibacterium* (*Crb*.), *Croceicoccus* (*Ccc*.) and *Qipengyuania* (*Qpy*.) were later proposed by Kwon *et al*. [38], Liu *et al.* [30], Xu *et al.* [4] and Feng *et al.* [2], respectively, based on 16S rRNA gene phylogeny [2, 4, 30, 38]. At the time of writing (September 2019), the genera *Altererythrobacter *, *Croceibacterium *, *Croceicoccus *, *Erythrobacter *, *Erythromicrobium *, *Porphyrobacter* and *Qipengyuania* consist of 41, two, four, 23, one, eight and one species, respectively [9, 10, 17, 19, 22, 30, 35, 39–43]. With the increase in the number of species proposed, the taxonomic status of the family *Erythrobacteraceae* should be revised in view of the polyphyletic nature of the group based on 16S rRNA gene sequence comparison [16, 30, 44]. The family *Erythrobacteraceae* includes aerobic anoxygenic phototrophic bacteria (AAPB), which can harvest light energy and play a significant role in the carbon cycling of the oceans globally [45–47]. Members of the family also show bioremediation and industrial potential, such as degradation of benzo[a]pyrene [48] and oil [49], and production of erythrazoles [50] and erythrolic acids [51]. A comprehensively taxonomic investigation of the family *Erythrobacteraceae* could not only lead to an improved classification of its members, but also broaden our understanding of their ecology and potential biotechnological applications.

Development of genome sequencing technologies has made bacterial genomic data more and more accessible, resulting in a revolution in bacterial taxonomy [52–54]. Phylogenomic reconstruction can provide a higher-resolution phylogeny than that based on 16S rRNA gene or several housekeeping genes [55–58]. In addition, genomic similarity calculations including average nucleotide identity (ANI), average amino acid identity (AAI) and percentage of conserved protein (POCP) provide numerical thresholds for delineation of each taxon [59–61]. Therefore, a genome-wide investigation of the taxonomy of the family *Erythrobacteraceae* was performed to revise the taxonomic status of this family.

## Methods

### Collection of *Erythrobacteraceae* type strains

In addition to the 47 *Erythrobacteraceae* type strains for which genome sequences were available, 27 type strains were obtained from culture collections including the China General Microbiological Culture Collection (CGMCC), the Deutsche Sammlung von Mikroorganismen und Zellkulturen GmbH (DSMZ), the Japan Collection of Microorganisms (JCM), the Korean Collection for Type Cultures (KCTC), the Korea Environmental Microorganism Bank (KEMB), the Collection of the Laboratorium voor Microbiologie en Microbiele Genetica (LMG) and the Marine Culture Collection of China (MCCC) or received as gifts from other scholars (Table 1 and Acknowledgements). These type strains were cultivated under appropriate conditions proposed previously [3, 12, 14, 20, 28, 31–33, 36, 44, 62–77] for subsequent genomic sequencing.

**Table 1.** Genomic information for *Erythrobacteraceae* strains included in this study DOE-JGI, U.S. Department of Energy, Joint Genome Institute; KRIBB, Korea Research Institute of Bioscience and Biotechnology; SDU, Shandong University.

Strain	NCBI GenBank Accession	Genome size (Mbp)	Gene count	Contig count	G+C content (%)	Reference
*Aeb. aerius* 100921-2^T^	WTZA00000000	2.75	2793	2	66.3	This study
*Aeb. aerophilus* Ery1^T^	QXFK00000000	3.65	3638	19	65.4	[17]
*Aeb. aestiaquae* KCTC 42006^T^	WTYZ00000000	2.87	2825	2	57.2	This study
*Aeb. aestuarii* JCM 16339^T^	WTYY00000000	2.24	2497	5	62.6	This study
*Aeb. amylolyticus* NS1^T^	CP032570	2.79	2791	1	67.0	[10]
*Aeb. aquaemixtae* KCTC 52763^T^	WTYX00000000	2.98	2933	4	58.5	This study
*Aeb. aquimixticola* SSKS-13^T^	SSHH00000000	3.43	3349	5	63.9	[40]
*Aeb. atlanticus* 26DY36^T^	CP011452, CP011453	3.51	3425	2	61.9	[120]
*Aeb. aurantiacus* MCCC 1A09962^T^	WTYW00000000	2.90	2907	7	61.2	This study
*Aeb. buctensis* M0322^T^	WTYV00000000	3.77	3734	22	66.0	This study
*Aeb. confluentis* KCTC 52259^T^	WTYU00000000	2.93	2892	5	59.1	This study
*Aeb. dongtanensis* KCTC 22672^T^	CP016591	3.01	2976	1	65.8	[121]
*Aeb. endophyticus* LMG 29518^T^	WTYT00000000	3.47	3314	13	58.6	This study
*Aeb. epoxidivorans* CGMCC 1.7731^T^	CP012669	2.79	2819	1	61.5	[48]
*Aeb. flavus* MS1-4^T^	PHSO00000000	3.28	3154	29	60.5	[7]
*Aeb. gangjinensis* JCM 17802^T^	WTYS00000000	2.89	2889	1	55.5	This study
*Aeb. halimionae* LMG 29519^T^	WTYR00000000	2.81	2778	2	63.6	This study
*Aeb. indicus* DSM 18604^T^	WTYQ00000000	3.11	3011	20	55.8	This study
*Aeb. insulae* BPTF-M16^T^	QURJ00000000	3.32	3997	1055	52.8	[41]
*Aeb. ishigakiensis* NBRC 107699^T^	CP015963	2.68	2670	1	56.9	[122]
*Aeb. luteolus* SW-109^T^	WTYP00000000	2.89	2841	3	59.3	This study
*Aeb. lutipelagi* GH1-16^T^	SKCJ00000000	3.10	3114	2	60.6	[42]
*Aeb. mangrovi* C9-11^T^	CP022889	2.70	2650	1	63.5	[6]
*Aeb. marensis* KCTC 22370^T^	CP011805	2.88	2784	1	64.7	KRIBB
*Aeb. marinus* H32^T^	WTYO00000000	3.00	2898	16	68.2	This study
*Aeb. maritimus* HME9302^T^	QBKA00000000	2.68	2737	2	60.8	[43]
*Aeb. namhicola* JCM 16345^T^	CP016545	2.59	2590	1	65.0	This study
*Aeb. oceanensis* MCCC 1A09965^T^	WTYN00000000	2.87	2892	14	63.9	This study
*Aeb. rigui* KCTC 42620^T^	RSEL00000000	2.86	2903	30	66.7	[112]
*Aeb. salegens* MCCC 1K01500^T^	WTYM00000000	3.63	3630	69	64.6	This study
*Aeb. sediminis* KCTC 42453^T^	WTYL00000000	3.16	3102	6	61.5	This study
*Aeb. soli* MCCC 1K02066^T^	WTYK00000000	3.08	2998	15	67.0	This study
*Aeb. troitsensis* JCM 17037^T^	LMAU00000000	2.90	2848	9	64.7	[123]
*Aeb. xiamenensis* CGMCC 1.12494^T^	FXWG00000000	3.09	3064	5	61.8	DOE-JGI
*Aeb. xinjiangensis* CCTCC AB 207166^T^	RSEK00000000	3.11	3153	59	64.2	[17]
*Aeb. xixiisoli* S36^T^	WTYJ00000000	3.88	3768	9	63.3	This study
*Crb. ferulae* SX2RGS8^T^	QZVQ00000000	3.61	3434	36	66.5	[30]
*Crb. mercuriale* Coronado^T^	JTDN00000000	3.48	3205	10	67.3	[124]
*Ccc. marinus* E4A9^T^	CP019602-CP019604	4.11	3956	3	64.5	[125]
*Ccc. mobilis* Ery22^T^	LYWZ00000000	4.21	4061	32	62.5	[5]
*Ccc. naphthovorans* PQ-2^T^	CP011770- CP011772	3.86	4007	3	62.6	[110]
*Ccc. pelagius* Ery9^T^	LYWY00000000	3.31	3264	40	62.8	[5]
*Erb. aquimaris* JCM 12189^T^	WTYI00000000	2.66	2680	3	61.8	This study
*Erb. aquimixticola* JSSK-14^T^	RAHX00000000	2.55	2633	2	63.0	[35]
*Erb. arachoides* RC4-10-4^T^	WTYH00000000	2.94	2929	1	65.4	This study
*Erb. atlanticus* s21-N3^T^	CP011310, CP015441	3.23	3296	2	58.3	[109]
*Erb. citreus* CGMCC 1.8703^T^	WTYG00000000	3.03	3045	24	64.2	This study
*Erb. gaetbuli* DSM 16225^T^	WTYF00000000	2.78	2752	4	64.1	This study
*Erb. gangjinensis* CGMCC 1.15024^T^	CP018097, CP018098	2.72	2695	2	62.7	[126]
*Erb. jejuensis* JCM 16677^T^	WTYE00000000	4.15	4124	1	60.2	This study
*Erb. litoralis* DSM 8509^T^	CP017057	3.25	3164	1	65.2	[127]
*Erb. longus* DSM 6997^T^	JMIW00000000	3.60	3430	14	57.4	[127]
*Erb. luteus* KA37^T^	LBHB00000000	2.89	2876	22	67.2	[118]
*Erb. lutimaris* S-5^T^	QRBB00000000	3.31	3219	12	65.5	SDU
*Erb. marinus* KCTC 23554^T^	LDCP00000000	2.84	2818	5	59.1	This study
*Erb. marisflavi* KEM-5^T^	VCAO00000000	2.67	2656	18	61.7	[22]
*Erb. nanhaisediminis* CGMCC 1.7715^T^	FOWZ00000000	2.90	2870	12	62.0	DOE-JGI
*Erb. odishensis* KCTC 23981^T^	QYOS00000000	3.19	3137	25	63.7	[9]
*Erb. pelagi* JCM 17468^T^	WTYD00000000	3.03	2936	9	64.2	This study
*Erb. seohaensis* SW-135^T^	CP024920	2.94	2919	1	61.7	[78]
*Erb. spongiae* HN-E23^T^	RPFZ00000000	2.86	2867	2	65.5	[35]
*Erb. vulgaris* DSM 17792^T^	WTYC00000000	3.23	3212	19	60.6	This study
*Erb. xanthus* CCTCC AB 2015396^T^	QXFM00000000	4.38	4320	151	64.5	[9]
*Erb. zhengii* V18^T^	QXFL00000000	3.80	3812	29	62.7	[9]
*Erm. ramosum* JCM 10282^T^	WTYB00000000	3.24	3175	10	64.3	This study
* Por. algicida* KEMB 9005-328^T^	WTYA00000000	3.22	3255	21	60.7	This study
* Por. colymbi* JCM 18338^T^	MUYK00000000	4.31	4092	53	66.5	[85]
* Por. cryptus* DSM 12079^T^	AUHC00000000	2.95	2902	36	67.9	DOE-JGI
* Por. dokdonensis* DSM 17193^T^	MUYI00000000	3.00	2885	13	64.8	[85]
* Por. donghaensis* DSM 16220^T^	MUYG00000000	3.37	3199	11	66.2	[85]
* Por. neustonensis* DSM 9434^T^	CP016033	3.09	2955	1	65.3	[128]
* Por. sanguineus* JCM 20691^T^	MUYH00000000	3.02	2931	34	63.6	[85]
* Por. tepidarius* DSM 10594^T^	MUYJ00000000	3.22	3151	32	65.9	[85]
*Qpy*. *sediminis* CGMCC 1.12928^T^	CP037948	2.42	2400	1	66.87	[129]

### Sequencing and assembly of genomic sequences

Genomic sequencing and assembly were performed as described previously [78]. Cells were harvested by centrifuge at 12,000 ***g*** for 30 s. Genomic DNA was extracted by using AxyPre Bacterial Genomic DNA Miniprep Kit (Corning Life Sciences) according to its manual. Genomes were sequenced on the HiSeq 2000 system (Illumina) by Solexa paired-end sequencing technology with a paired-end library with insert length of 500 bp by the Novogene Corporation (Beijing, PR China). Draft genomes were assembled by using SPAdes version 3.11.1 [79] based on clean reads generated from raw reads by quality trimming. The collection of assembled and obtained genomes covered 92 % (74/80) of the *Erythrobacteraceae* type strains, comprising 88 % (36/41), 100 % (2/2), 100 % (4/4), 96 % (22/23), 100 % (1/1), 100 % (8/8) and 100 % (1/1) of the genera *Altererythrobacter *, *Croceibacterium *, *Croceicoccus *, *Erythrobacter *, *Erythromicrobium *, *Porphyrobacter* and *Qipengyuania *.

### Genomic annotation and comparative genomic analysis

Genomes for annotation and comparative analysis were selected following assessment of genomic completeness (>95 %) and contamination (<5 %) using CheckM software version 1.0.7 [80] with the command ‘checkm lineage_wf -x fasta bins/ checkm/’. rRNA and tRNA genes were searched by the command RNAmmer 1.2 package [81] and the tRNAscan-SE web server (http://lowelab.ucsc.edu/tRNAscan-SE/) [82], respectively. Annotated 16S rRNA genes were used to compare sequence identities on the EzBioCloud web server (www.ezbiocloud.net/identify) [83] to confirm that a genome represented its corresponding type strain. Coding sequences (CDSs) were predicted and annotated by using Rapid Annotation using Subsystem Technology (RAST) web server version 2.0 (http://rast.nmpdr.org/rast.cgi) [84]. The DNA G+C contents were also calculated on the RAST web server version 2.0.

Comparative genomic analysis was performed as previously described [85, 86]. Orthologous clusters (OCs) were identified by comparing whole protein sequences translated from CDSs pairwise with the execution of Proteinortho version 5.16b [87] with command ‘-e 1e-5 -cov=50 -identity=50’, which is accordance with the threshold values for a group of OCs sharing identities more than 50 % and coverage longer than half of their sequence lengths. Subsequently, single-copy OCs were filtered by an in-house Perl script.

### 16s rRNA gene phylogenetic and phylogenomic reconstructions

In accordance with previous polyphasic taxonomic studies of the members in the family *Erythrobacteraceae* [19, 22, 35, 40, 88], *Rhodospirillum rubrum* ATCC 11170^T^ was chosen as an outgroup, with its 16S rRNA gene sequence and genomic sequences obtained from the NCBI GenBank database under the accession numbers D30778 and CP000230–CP000231, respectively. 16S rRNA gene phylogeny was reconstructed as described by Xu *et al.* [89]. Gene sequences of 80 *Erythrobacteraceae* type strains and an outgroup were aligned with clustal_w [90] built in in the mega7 software [91]. Then, aligned sequences were processed into maximum-likelihood phylogenetic analysis [92], using mega7 software with the substitution model and the bootstrap value set as Kimura's two-parameter model [93] and 1000 replicates, respectively.

Protein and gene sequences of filtered single-copy OCs were both performed in the phylogenomic analyses. Protein sequences were aligned by using mafft version 7 [94] with the parameter ‘-auto’, while gene sequences were aligned by mapping nucleotides on amino acids based on aligned protein sequences through PAL2NAL program version 14 [95]. Aligned sequences were refined to select the most reliable positions through trimAL version 1.4.1 [96] with the parameter ‘-automated1’ and concatenated through our in-house perl script. Concatenation and partition methods were both applied in this study. The best substitution models for were proposed by IQ-Tree 1.6.1 software [97] with the command ‘-m MFP’. Subsequently, LG+F+R9 and GTR+F+R8 were estimated as the best substitution models for concatenations of amino acid and nucleotide sequences, respectively, and the best substitution models for partition methods are listed in Table S2. Maximum-likelihood phylogenomic trees were reconstructed by using IQ-Tree 1.6.1 software [97] with the bootstrap value set to 100 replicates.

### Genomic similarity analysis

ANI, AAI and POCP values were used to calculate genomic similarities. ANI values were calculated by the orthologous average nucleotide identity tool (OrthoANI version 0.93.1) [98] implemented with the blast algorithm [99]. AAI values were obtained using the Kostas lab AAI calculator web server (http://enve-omics.ce.gatech.edu/aai/) [100]. POCP values were obtained according to the formula ‘POCP=(*C1 +C2*)/(*T1 +T2*)×100 %’ where *C1* and *C2* indicated the conserved number of predicted proteins in the two pairwise compared genomes, respectively, as well as T1 and T2 stands for the total number of predicted proteins in the two pairwise compared genomes, respectively [59], following comparative genomic analysis by using Proteinortho version 5.16b with the command ‘-e=1e-5 -cov=50 -identity=40’. In addition, the t-tests of AAI, ANI and POCP values of inter- and intra-group were calculated by using the function ‘*t.text*’ within R version 3.4.2 [101].

## Discussion

### Characteristics of *Erythrobacteraceae* genomes

All obtained genomes were of high quality with genomic completeness of 97.6–99.9 % (average 99.3 %; median 99.4 %) and contamination of 0–4.9 % (average 0.7 %; median 0.4 %), as shown in Table S2. Sequence identity analysis of annotated 16S rRNA genes from the genomes sequenced in this study indicated that each represented its type strain with high identities of 99.2–100.0 % (Table S3). Several strains, including *Aeb. confluentis* KCTC 52259^T^, *Aeb. indicus* DSM 18604^T^, *Aeb. luteolus* SW-109^T^, *Erb. citreus* CGMCC 1.8703^T^ and *Erb. jejuensis* JCM 16677^T^, had multi-copy 16S rRNA genes, whose sequences were identical.

**Fig. 1.**
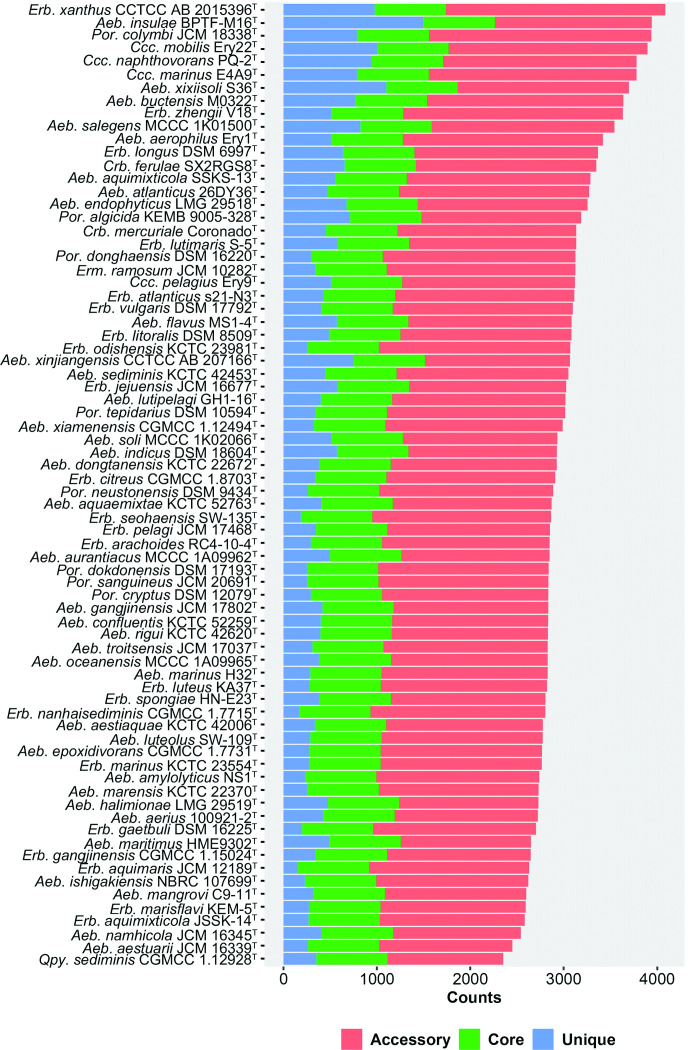
Accessory, core and unique OCs distributed in each type strain belonging to the family *Erythrobacteraceae *.

Genomic sizes, gene counts and G+C contents were 2.24–4.38 Mbp (average 3.16 Mbp; median 3.06 Mbp), 2400–4320 (average 3117; median 2987) and 52.8–68.2 % (average 63.0 %; median 63.6 %), respectively (Table 1). Comparative genomic analysis revealed that the pan-genome of the family *Erythrobacteraceae* harboured 49, 006 OCs, among which 763 OCs were shared by all type strains, which also had 1,233–2,375 accessory and 157–1,500 unique OCs (Fig. 1). The percentages of accessory, core and unique OCs in each type strain varied greatly with values of 18.7–32.5, 42.6–66.7 and 6.0–38.1 %, respectively, which showed a rich genetic diversity in this family. A total of 288 single-copy OCs (Table S4) were included in our phylogenomic analyses.

### 16s rRNA gene phylogeny

As stated before, several genera within the family *Erythrobacteraceae* did not form an independent clade in the 16S rRNA gene phylogenetic tree (Fig. S1): (1) the genus *Erythrobacter *, being the type genus of the family, could be divided into four clades, one of which was grouped with the genera *Erythromicrobium* and *Porphyrobacter *; (2) the genus *Altererythrobacter* showed five clades which also included the genera *Croceicoccus* and *Qipengyuania *; (3) the genera *Erythromicrobium* and *Qipengyuania *, each consisting of a single species, were clustered in clades mostly containing of *Porphyrobacter* and *Altererythrobacter *, respectively. The genera *Croceibacterium* and *Croceicoccus* formed two independent clades, and they did not belong to monophyletic clades which could be separated from other genera. Thus, 16S rRNA gene sequences did not confirm monophyletic relationships within the genera of the family [2, 4, 30, 37, 38]. Only 19 nodes accounting for 26.0 % exhibited bootstrap values higher than 70 %, indicating that this phylogenetic tree was not reliable enough to correctly reveal the taxonomic status of the genera of the family.

### Phylogenomic and genomic similarity analyses proposing three clades

**Fig. 2.**
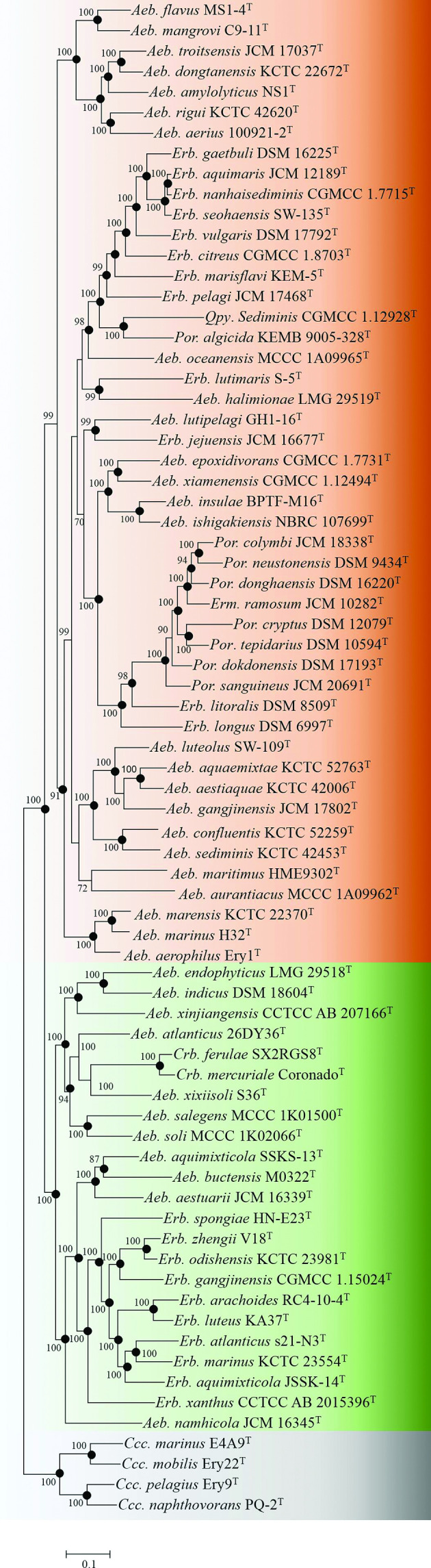
A maximum-likelihood tree based on the partition of 288 single-copy OC protein sequences showing the phylogenetic relationship of type strains belonging to the family *Erythrobacteraceae *. Bootstrap values are based on 100 replicates. Bar., 0.1 substitutions per nucleotide position. The backgrounds coloured brown, green and grey indicate Clades I, II and III, respectively. *Rhodospirillum rubrum* ATCC 11170^T^ was used as an outgroup (not shown).

Four phylogenomic trees, based on 288 single-copy OC, amino acid or nucleotide sequences, with annotations and substitution models are shown in Table S4 and had similar topological structures with 59 nodes accounting for 80.8 %, and were identical in all four calculated phylogenetic relationship parameters (Fig. 2 and Figs. S2–4). In all four phylogenomic trees, the bootstrap value of most nodes (66/73–68/73) exceeded 70 %, indicating those phylogenies were robust. Compared with 16S rRNA gene phylogeny, those similar and robust phylogenomic trees could provide a reliable taxonomic status for the family *Erythrobacteraceae *.

**Fig. 3.**
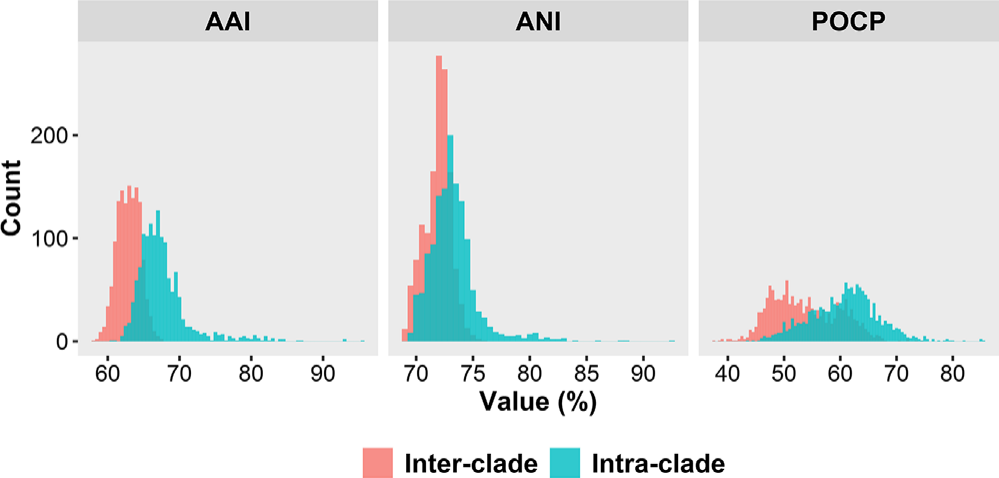
Histograms of AAI, ANI and POCP values regarding inter- and intra-clade. Red and blue indicate inter-clade and intra-clade, respectively.

Based on these phylogenomic trees, the family *Erythrobacteraceae* can be divided into three separate clades, Clades I, II and III, consisting of 47, 23 and four species, respectively (Fig. 2). Genomic similarity analyses by AAI, ANI and POCP calculations also supported that the three clades were significantly separated with *p* value<2.2×10^−16^ (Fig. 3). Clades I and II contained most species. Clade III only contained four *Croceicoccus* species, indicating that the taxonomic status of this genus should not be changed.

### AAI value and evolutionary distance classifying genera

**Fig. 4.**
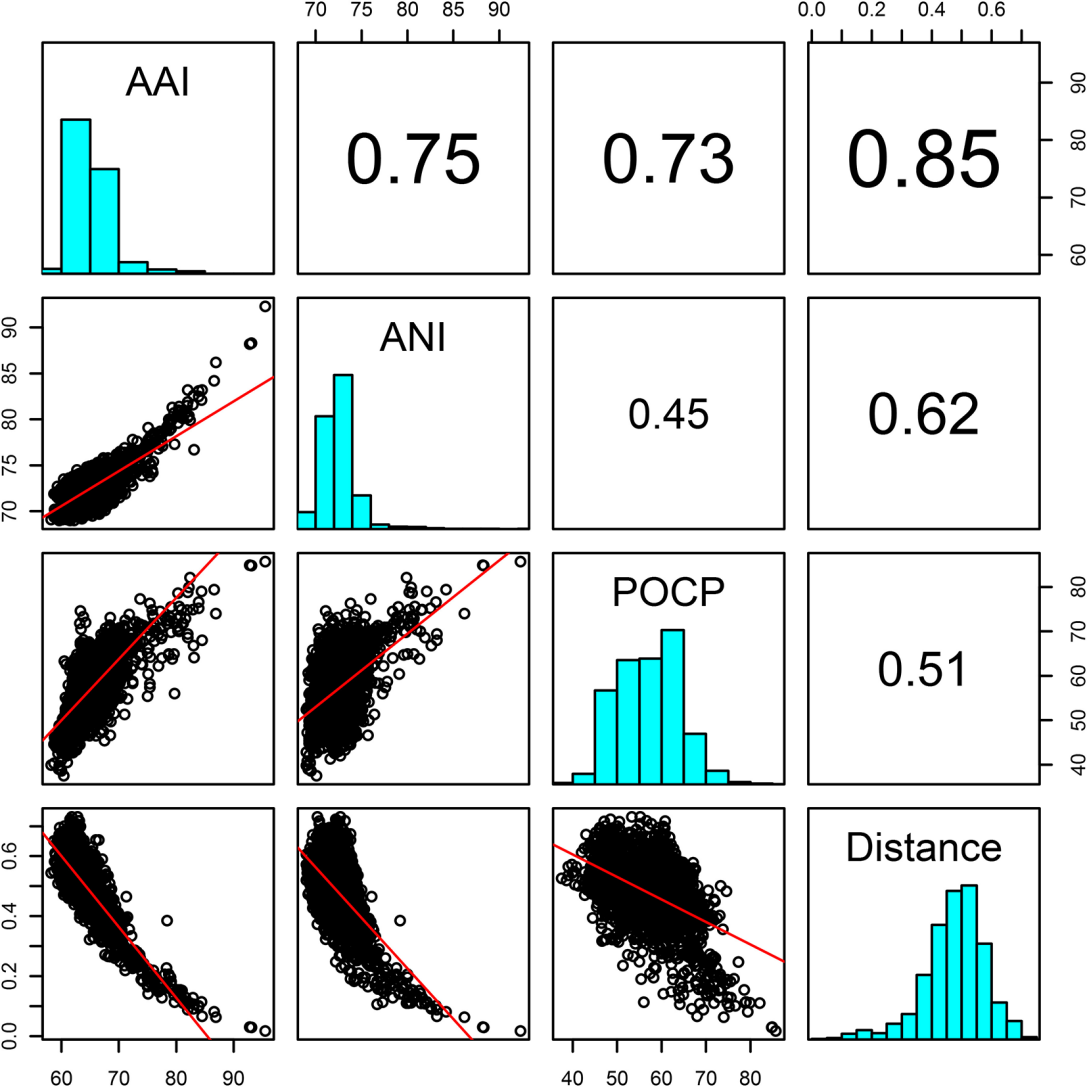
Pairwise correlations of ANI, AAI, POCP and evolutionary distance calculated from the genomes of *Erythrobacteraceae* type strains.

A robust core-genome phylogeny of the family was obtained. Although there is no generally recognized genus boundary, recent studies suggested AAI (60–80 %) and POCP (50 %) could be thresholds for distinguishing genera [59, 61]. Evolutionary distance is also a relatively conserved criterion for inferring evolutionary relationships [102]. Pairwise comparisons of ANI, AAI, POCP and evolutionary distance indicated that the pair of AAI and evolutionary distance had a much higher correlation coefficient (*r_cc_*=0.85) than other pairs (Fig. 4). Type strains shared pairwise >50 % of POCP values, which is similar to the result of a phylogenomic study of the *Roseobacter* group [58], suggesting that POCP values could not be applied for delineating genera within the family *Erythrobacteraceae *. AAI was more suitable to distinguish each taxon in the family than ANI and POCP (Figs. S5–S7). Thus, AAI and evolutionary distance were selected to classify genera of *Erythrobacteraceae *.

**Fig. 5.**
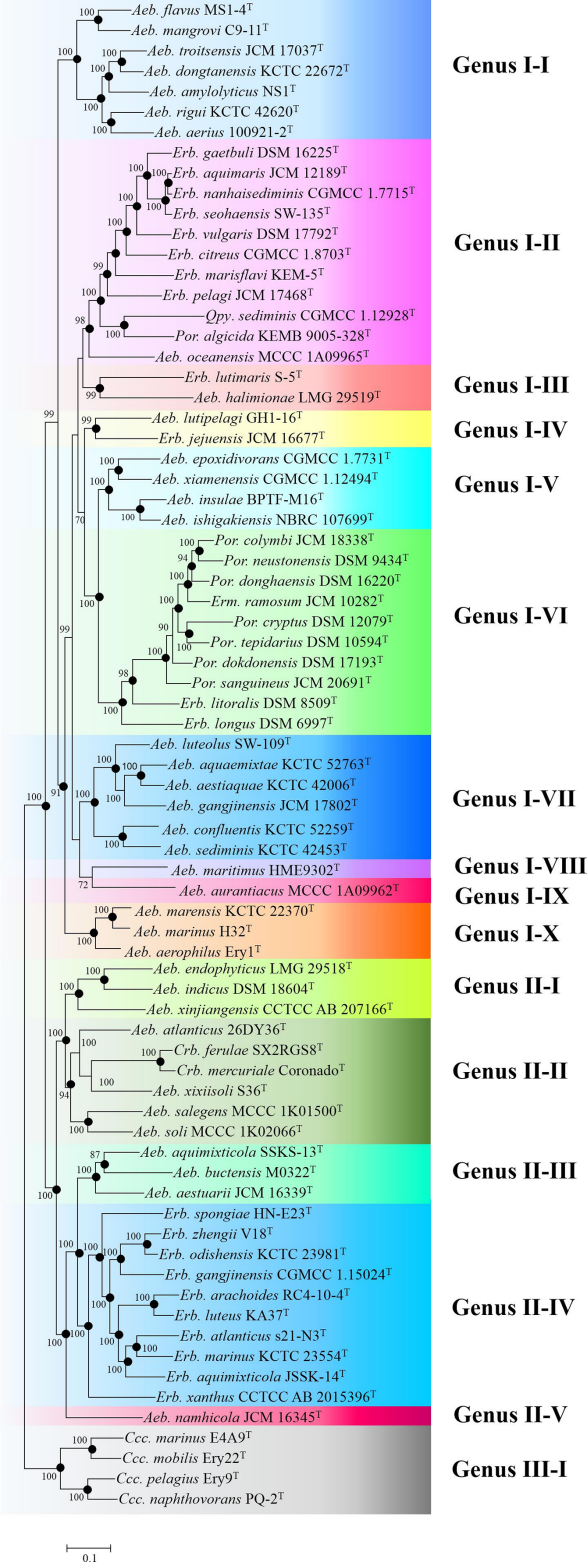
Proposed taxonomic status for the family *Erythrobacteraceae *. Maximum-likelihood tree based on the partition of 288 single-copy OC protein sequences. Bootstrap values are based on 100 replicates. Bar, 0.1 substitutions per nucleotide position.

**Fig. 6.**
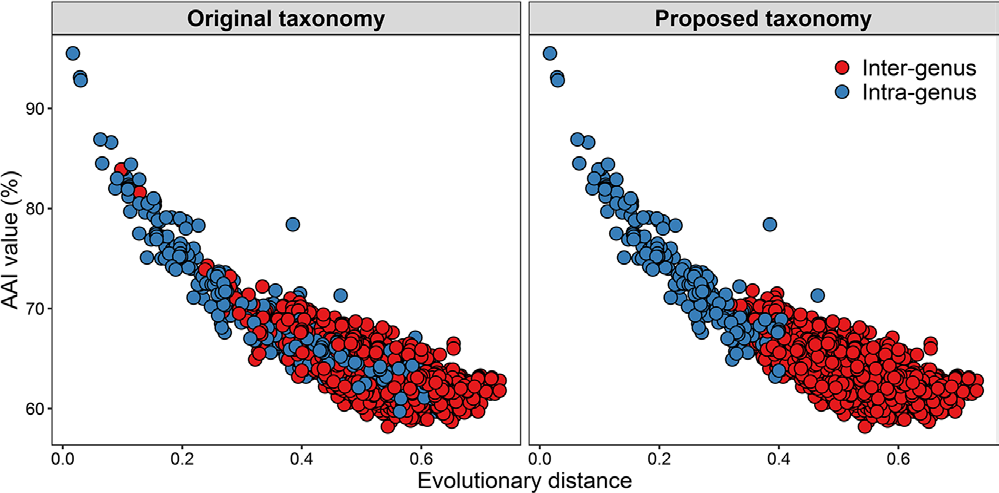
Comparison of original and proposed taxonomy for the family *Erythrobacteraceae* based on AAI values and evolutionary distances. Red and blue indicate inter-genus and intra-genus, respectively.

Since phylogenetic tree topology is a major criterion for classifying genera, we propose that one genus should be clustered into one group only. Based on this criterion, Clade I is then composed of 10 genera (Fig. 5), including Genus I-I (*Aeb. flavus* MS1-4^T^, *Aeb. mangrovi* C9-11^T^, *Aeb. troitsensis* JCM 17037^T^, *Aeb. dongtanensis* KCTC 22672^T^, *Aeb. amylolyticus* NS1^T^, *Aeb. rigui* KCTC 42620^T^ and *Aeb. aerius* 10092102^T^), Genus I-II (*Erb. gaetbuli* DSM 16225^T^, *Erb. aquimaris* JCM 12189^T^, *Erb. nanhaisediminis* CGMCC 1.7715^T^, *Erb. seohaensis* SW-135^T^, *Erb. vulgaris* DSM 17792^T^, *Erb. citreus* CGMCC 1.8703^T^, *Erb. marisflavi* KEM-5^T^, *Erb. pelagi* JCM 17468^T^, *Qpy. sediminis* M1^T^, *Por. algicida* KEMB 9005-328^T^ and *Aeb. oceanensis* MCCC 1A09965^T^), Genus I-III (*Erb. lutimaris* S-5^T^ and *Aeb. halimionae* LMG 29519^T^), Genus I-IV (*Aeb. lutipelagi* GH1-6^T^ and *Erb. jejuensis* JCM 16677^T^), Genus I-VI (*Aeb. epoxidivorans* CGMCC 1.7731^T^, *Aeb. xiamensis* CGMCC 1.12494^T^, *Aeb. insulae* BPTF-M16^T^ and *Aeb. ishigakiensis* NBRC 107699^T^), Genus I-VII (* Por. colymbi* JCM 18338^T^, *Por. neustonensis* DSM 9434^T^, *Por. donghaensis* DSM 16220^T^, *Erm. ramosum* JCM 10282^T^, *Por. cryptus* DSM 12079^T^, *Por. tepidarius* DSM 10594^T^, *Por. dokdonensis* DSM 17193^T^, *Por. sanguineus* JCM 20691^T^, *Erb. litoralis* DSM 8509^T^ and *Erb. longus* DSM 6997^T^), Genus I-VIII (*Aeb. luteolus* SW-109^T^, *Aeb. aquaemixtae* KCTC 52763^T^, *Aeb. aestiaquae* KCTC 42006^T^, *Aeb. gangjinensis* JCM 17802^T^, *Aeb. confluentis* KCTC 52259^T^ and *Aeb. sediminis* KCTC 42453^T^), Genus I-IX (*Aeb. maritimus* HME 9302^T^) and Genus I-X (*Aeb. aurantiacus* MCCC 1A09962^T^). The type strains of each of these genera exhibited pairwise evolutionary distance <0.4 %, except for *Aeb. oceanensis* MCCC 1A09965^T^ and *Qpy. sediminis* M1^T^. These two type strains also showed a pairwise AAI value of 67.3 %, while the pairwise AAI value for the majority of this clade (96.8 %, 1047/1081) were higher than 70%. Clade III consisted of one genus, whose species had AAI values of 68.1–77.5 % and evolutionary distances of 0.13–0.27. Based on the analysis of these two clades, the genus boundary for the family is here proposed as AAI values of 70% and an evolutionary distance of 0.4 (Fig. 6).

Based on these criteria, Clade II with all nodes with bootstrap values of >85 % could be divided into five genera (Fig. 5), consisting of Genus II-I (*Aeb. endophyticus* LMG 29518^T^, *Aeb. indicus* DSM 18604^T^ and *Aeb. xinjiangensis* CCTCC AB 207166^T^), Genus II-II (*Aeb. atlanticus* 26DY36^T^, *Crb. ferulae* SX2RGS8^T^, *Crb. mercuriale* Coronado^T^, *Aeb. xixiisoli* S36^T^, *Aeb. salegens* MCCC 1K01500^T^, *Aeb. soli* MCCC 1K02066^T^), Genus II-III (*Aeb. aquimixticola* SSKS-13^T^, *Aeb. buctensis* M0322^T^ and *Aeb. aestuarii* JCM 16339^T^), Genus II-IV (*Erb. spongiae* HN-E23^T^, *Erb. zhengii* V18^T^, *Erb. odishensis* KCTC 23981^T^, *Erb. gangjiensis* CGMCC 1.15204^T^, *Erb. arachoides* RC4-10-4^T^, *Erb. luteus* KA37^T^, *Erb. atlanticus* s21-N3^T^, *Erb. marinus* KCTC 23554^T^, *Erb. aquimixticola* JSSK-14^T^ and *Erb. xanthus* CCTCC AB 2015396^T^) and Genus II-V (*Aeb. namhicola* 16345^T^).

We therefore propose that phylogenomic topology supplemented with AAI values and evolutionary distance values could replace the phylogeny based on 16S rRNA gene sequences in the taxonomy of the family *Erythrobacteraceae *.

### Genotype and phenotype support the proposal of new genera

Comparison of genomic contents within the family *Erythrobacteraceae* revealed that 12 443 OCs could be indicators for distinguishing newly proposed genera. While considerable metabolic diversity is found within the family, metabolic pathways involving carbon, nitrogen, phosphorus and sulfur could not be applied to refine the taxonomic status of this family. Therefore, the pathways of aerobic anoxygenic photosynthesis and flagella biosynthesis, which contain multiple genes and reactions [47, 103], were selected to investigate their value as indicators for their taxonomic status.

Aerobic anoxygenic photosynthesis is encoded by a series of genes that were found in all Genus I-VI species, *Aeb. ishigakiensis* NBRC 107699^T^ (Genus I-V), *Erb. marinus* KCTC 23554^T^ (Genus II-III) and *Erb. odishensis* KCTC 239891^T^ (Genus II-III), as shown in Table 2. Phenotypic characteristics revealed that Genus I-VI consisted of AAPB [23, 24, 26, 27, 29, 31, 104–106], while other genera did not include AAPB. Moreover, phylogenetic analysis indicated that genes for aerobic anoxygenic photosynthesis of *Aeb. ishigakiensis* NBRC 107699^T^, *Erb. marinus* KCTC 23554^T^ and *Erb. odishensis* KCTC 239891^T^ were paralogs of such genes in Genus I-VI (Fig. S8).

**Table 2.** Comparisons of genotype and phenotype regarding aerobic anoxygenic photosynthesis and flagella biosynthesis in the family *Erythrobacteraceae
* Genera: 1, Genus I-I; 2, Genus I-II; 3, Genus I-III; 4, Genus I-IV; 5, Genus I-V; 6, Genus I-VI; 7, Genus I-VII; 8, Genus I-VIII; 9, Genus I-IX; 10, Genus I-X; 11, Genus II-I; 12, Genus II-II; 13, Genus II-III; 14, Genus II-IV; 15, Genus II-V; 16, Genus III. +, Gene detected; −, gene not detected.

Pathway	1	2	3	4	5	6	7	8	9	10	11	12	13	14	15	16
Aerobic anoxygenic photosynthesis																
*bchD*	−	−	+	−	+	+	+	−	−	−	−	−	−	+	−	−
*bchE*	−	−	+	−	+	+	+	−	−	−	−	−	−	+	−	−
*bchG*	−	−	+	−	+	+	+	−	−	−	−	−	−	+	−	−
*bchL*	−	−	+	−	+	+	+	−	−	−	−	−	−	+	−	−
*bchM*	−	−	+	−	+	+	+	−	−	−	−	−	−	+	−	−
*bchN*	−	−	+	−	+	+	+	−	−	−	−	−	−	+	−	−
*bchP*	−	−	+	−	+	+	+	−	−	−	−	−	−	+	−	−
*bchX*	−	−	+	−	+	+	+	−	−	−	−	−	−	+	−	−
*bchY*	−	−	+	−	+	+	+	−	−	−	−	−	−	+	−	−
*bchZ*	−	−	+	−	+	+	+	−	−	−	−	−	−	+	−	−
*hemD*	+	+	−	+	+	+	+	+	+	+	+	+	+	+	+	+
*hemF*	+	+	+	+	+	+	+	−	−	−	+	+	+	+	−	+
*pufL*	−	−	−	−	+	+	−	−	−	−	−	−	−	+	−	−
Phenotype	−	−	−	−	−	+	−	−	−	−	−	−	−	−	−	−
Flagella biosynthesis																
*flgA*	−	+	−	−	−	−	−	−	−	−	−	−	−	−	−	−
*flgB*	−	+	−	−	−	+	−	−	−	+	−	+	−	+	−	+
*flgC*	−	+	−	−	−	+	−	−	−	+	−	+	−	+	−	+
*flgD*	−	+	−	−	−	+	−	−	−	+	−	+	−	+	−	+
*flgE*	−	+	−	−	−	+	−	−	−	+	−	+	−	+	−	+
*flgF*	−	+	−	−	−	+	−	−	−	+	−	+	−	+	−	+
*flgG*	−	+	−	−	−	+	−	−	−	+	−	+	−	+	−	+
*flgH*	−	+	−	−	−	+	−	−	−	+	−	+	−	+	−	+
*flgI*	−	+	−	−	−	+	−	−	−	+	−	+	−	+	−	+
*flgJ*	−	+	−	−	−	+	−	−	−	+	−	+	−	+	−	+
*flgK*	−	+	−	−	−	−	−	−	−	+	−	+	−	+	−	+
*flgL*	−	+	−	−	−	−	−	−	−	+	−	+	−	+	−	+
*flhA*	−	+	−	−	−	+	−	−	−	+	−	+	−	+	−	+
*flhB*	−	+	−	−	−	+	−	−	−	+	−	+	−	+	−	+
*fliC*	−	+	−	−	−	+	−	−	−	+	−	+	−	+	−	+
*fliD*	−	+	−	−	−	+	−	−	−	+	−	+	−	+	−	+
*fliE*	−	+	−	−	−	+	−	−	−	+	−	+	−	+	−	+
*fliF*	−	+	−	−	−	+	−	−	−	+	−	+	−	+	−	+
*fliG*	−	+	−	−	−	+	−	−	−	+	−	+	−	+	−	+
*fliL*	−	+	−	−	−	+	−	−	−	+	−	+	−	+	−	+
*fliM*	−	+	−	−	−	−	−	−	−	−	−	−	−	−	−	−
*fliN*	−	+	−	−	−	+	−	−	−	+	−	+	−	+	−	+
*fliP*	−	+	−	−	−	+	−	−	−	+	−	+	−	+	−	+
*fliQ*	−	+	−	−	−	+	−	−	−	+	−	+	−	+	−	+
*fliR*	−	+	−	−	−	+	−	−	−	+	−	+	−	+	−	+
Phenotype	−	+	−	−	−	+	−	−	−	+	−	+	−	+	−	+

Flagella can be used for locomotion and sensing, which improves the survival of prokaryotes [107, 108]. Comparison of gene contents showed that several strains in Genus I-II (* Por. algicida* KEMB 9005-328^T^), Genus I-VI (*Erb. litoralis* DSM 8509^T^, *Erb. longus* DSM 6997^T^, *Erm. ramosum* JCM 10282^T^, *Por. colymbi* JCM 18338^T^, *Por. cryptus* DSM 12079^T^, *Por. neustonensis* DSM 9434^T^ and *Por. sanguineus* JCM 20691^T^), Genus I-X (*Aeb. marinus* H32^T^), Genus II-II (*Aeb. atlanticus* 26DY36^T^ and *Aeb. soli* MCCC 1K02066^T^), Genus II-IV (*Erb. atlanticus* s21-N3^T^) and Genus III (*Ccc. mobilis* Ery22^T^ and *Ccc. naphthovorans* PQ-2^T^) had genes related to flagella biosynthesis. Microscopic observations showed flagella in those strains [5, 8, 14, 23, 24, 26, 29, 31, 44, 104, 109, 110], except for *Aeb. marinus* H32^T^.

Based on the phylogenomic and genomic similarity analyses, we propose that the family *Erythrobacteraceae* could be reclassfied into 16 genera including 11 novel genera, for which the names *Alteraurantiacibacter*, *Altericroceibacterium*, *Alteriqipengyuania*, *Alteripontixanthobacter*, *Aurantiacibacter*, *Paraurantiacibacter*, *Parapontixanthobacter*, *Parerythrobacter*, *Pelagerythrobacter*, *Pontixanthobacter* and *Tsuneonella* are proposed. Because the species of *Erythromicrobium* and *Porphyrobacter* were merged into the genus *Erythrobacter *, those two genera are no longer necessary in taxonomic discussions, but their names remain validly published and can still be used.

## Description of *Tsuneonella* gen. nov.

*Tsuneonella* (Tsu.ne.o.nel'la. N.L. fem. n. *Tsuneonella*, named in honour of Tsuneo Shiba who established genus *Erythrobacter *).

Cells are Gram-stain-negative, ovoid to rod, non-spore-forming and non-motile. Aerobic or facultatively aerobic. Contains carotenoid pigments but not bacteriochlorophyll *a*. The predominant ubiquinone is Q-10. The major fatty acid (>10 %) is summed feature 8 (C_18 : 1_ *ω*7*c* and/or C_18 : 1_ *ω*6*c*). The major polar lipids are diphosphatidylglycerol and phosphatidylethanolamine. The genus represents a distinct branch in the family *Erythrobacteraceae* of the class *Alphaproteobacteria* based on the core-genomic phylogeny. The DNA G+C content is 60.5–67.0 % (by genome). The type species is *Tsuneonella dongtanensis*.

## Description of *Tsuneonella aeria* comb. nov.

*Tsuneonella aeria* (a.e'ri.a. L. fem. adj. *aeria* pertaining to the air, aerial).

Basonym: *Altererythrobacter aerius* Xue *et al.* 2016.

The description is the same as for *Aeb. aerius* [28]. Core-genomic phylogenetic analysis strongly supported the placement of this species in the genus *Tsuneonella*. The type strain, 100921-2^T^ (=CFCC 14287^T^=KCTC 42844^T^), was isolated from air at the foot of Xiangshan Mountain, Beijing, PR China. The DNA G+C content of the type strain is 66.3 % (by genome).

## Description of *Tsuneonella amylolytica* comb. nov.

*Tsuneonella amylolytica* (a.my.lo.ly'ti.ca. Gr. neut. n. *amylon* starch; Gr. fem. adj. *lytikê* able to loosen, able to dissolve; N.L. fem. adj. *amylolytica* starch dissolving).

Basonym: *Altererythrobacter amylolyticus* Qu *et al*. 2019.

The description is the same as for *Aeb. amylolyticus* [10]. Core-genomic phylogenetic analysis strongly supported the placement of this species in the genus *Tsuneonella*. The type strain, NS1^T^ (=CGMCC 1.13679^T^=NBRC 113553^T^), was isolated from sediment of Taihu Lake in Jiangsu Province, PR China. The DNA G+C content of type strain 67.0 % (by genome).

## Description of *Tsuneonella dongtanensis* comb. nov.

*Tsuneonella dongtanensis* (dong.tan.en'sis. N.L. fem. adj. *dongtanensis* pertaining to Dongtan, a wetland region in Chongming Island, Shanghai, PR China).

Basonym: *Altererythrobacter dongtanensis* Fan *et al.* 2011.

The description is the same as for *Aeb. dongtanensis* [111]. Core-genomic phylogenetic analysis strongly supported the placement of this species in the genus *Tsuneonella*. The type strain, JM27^T^ (=KCTC 22672^T^=CCTCC AB 209199^T^), was isolated from a tidal flat (Dongtan Wetland, Chongming Island, Shanghai, PR China). The DNA G+C content of the type strain is 65.8 % (by genome).

## Description of *Tsuneonella flava* comb. nov.

*Tsuneonella flava* (fla'va. L. fem. adj. *flava* yellow, the colour of colonies and pigments of the bacterium).

Basonym: *Altererythrobacter flavus* Ma *et al*. 2018.

The description is the same as for *Aeb. flavus* [44]. Core-genomic phylogenetic analysis strongly supported the placement of this species in the genus *Tsuneonella*. The type strain, MS1-4^T^ (=MCCC 1K02683^T^=NBRC 112977^T^), was isolated from mangrove sediment of the Jiulong River Estuary, Fujian Province, PR China. The DNA G+C content of the type strain is 60.5 % (by genome).

## Description of *Tsuneonella mangrovi* comb. nov.

*Tsuneonella mangrovi* (man.gro'vi. N.L. gen. n. *mangrovi* of or belonging to a mangrove wetland).

Basonym: *Altererythrobacter mangrovi* Liao *et al*. 2017.

The description is the same as for *Aeb. mangrovi* [6]. Core-genomic phylogenetic analysis strongly supported the placement of this species in the genus *Tsuneonella*. The type strain, C9-11^T^ (=MCCC 1K03311^T^=JCM 32056^T^), was isolated from a mangrove sediment sample collected from Yunxiao Mangrove National Nature Reverse in Zhangzhou, Fujian Province, PR China. The DNA G+C content of the type strain is 63.5 % (by genome).

## Description of *Tsuneonella rigui* comb. nov.

*Tsuneonella rigui* (ri’gu.i. L. gen. n. *rigui* of a well-watered place).

Basonym: *Altererythrobacter rigui* Kang *et al.* 2016.

The description is the same as for *Aeb. rigui* [112]. Core-genomic phylogenetic analysis strongly supported the placement of this species in the genus *Tsuneonella*. The type strain, WW3^T^ (=KCTC 42620^T^=JCM 30975^T^), was isolated from freshwater of Woopo wetland, Republic of Korea. The DNA G+C content of the type strain is 66.7 % (by genome).

## Description of *Tsuneonella troitsensis* comb. nov.

*Tsuneonella troitsensis* (troi.tsen'sis. N.L. fem. adj. *troitsensis* referring to Troitsa Bay, from where the organism was isolated).

Basonym: *Altererythrobacter troitsensis* Nedashkovskaya *et al*. 2013.

The description is the same as for *Aeb. troitsensis* [34]. Core-genomic phylogenetic analysis strongly supported the placement of this species in the genus *Tsuneonella*. The type strain, KMM 6042^T^ (=KCTC 12303^T^=JCM 17037^T^), was isolated from the sea urchin *Strongylocentrotus intermedius*. The DNA G+C content of the type strain is 64.7 % (by genome).

## Emended description of the genus *Qipengyuania* Feng *et al.* 2015

The description is as given by Feng *et al.* [2] with the following amendment. Cells are aerobic or facultatively aerobic. Contains carotenoid pigments but not bacteriochlorophyll *a*. Positive or negative for oxidase. The major fatty acid (>10%) is summed feature 8 (C_18 : 1_ *ω*7*c* and/or C_18 : 1_ *ω*6*c*). The major polar lipids are phosphatidylcholine, phosphatidylethanolamine and phosphatidylglycerol. The genus represents a distinct branch in the family *Erythrobacteraceae* of the class *Alphaproteobacteria* based on the core-genomic phylogeny. The DNA G+C content is 60.6–66.7 % (by genome). The type species for the genus is *Qipengyuania sediminis *.

## Description of *Qipengyuania algicida* comb. nov.

*Qipengyuania algicida* (al.gi.ci’da. L. fem. n. *alga* alga; L. suff. –*cida* from L. v. *caedere* to kill; N.L. fem. n. *algicida* a killer of algae).

Basonym: *Porphyrobacter algicida* Kristyanto *et al*. 2017.

The description is the same as for *Por. algicida* [44]. Core-genomic phylogenetic analysis strongly supported the placement of this species in the genus *Qipengyuania *. The type strain, Yeonmyeong 2-22^T^ (=KEMB 9005–328^T^=JCM 31499^T^), was isolated from surface seawater collected from Geoje Island in the South Sea, Republic of Korea. The DNA G+C content of the type strain is 60.7 % (by genome).

## Description of *Qipengyunia aquimaris* comb. nov.

*Qipengyuania aquimaris* (a.qui.ma'ris. L. fem. n. *aqua* water; L. neut. n. *mare* the sea; N.L. gen. n. *aquimaris* of the water of the sea).

Basonym: *Erythrobacter aquimaris* Yoon *et al*. 2004.

The description is the same as for *Erb. aquimaris* [73]. Core-genomic phylogenetic analysis strongly supported the placement of this species in the genus *Qipengyuania *. The type strain, SW-110^T^ (=KCCM 41818^T^=JCM 12189^T^), was isolated from sea water of a tidal flat of the Yellow Sea in the Republic of Korea. The DNA G+C content of the type strain is 61.8 % (by genome).

## Description of *Qipengyuania citrea* comb. nov.

*Qipengyuania citrea* (ci'tre.a. L. fem. adj. *citrea*, describing the lemon-yellow pigmentation).

Basonym: *Erythrobacter citreus* Denner *et al*. 2002.

The description is the same as for *Erb. citreus* [75]. Core-genomic phylogenetic analysis strongly supported the placement of this species in the genus *Qipengyuania *. The type strain, RE35/F1^T^ (=CIP 107092^T^=DSM 14432^T^=JCM 21816^T^), was isolated from the western Mediterranean Sea (Bay of Calvi, Corsica, France). The DNA G+C content of the type strain is 64.2 % (by genome).

## Description of *Qipengyuania gaetbuli* comb. nov.

*Qipengyuania gaetbuli* (gaet.bu'li. N.L. gen. n. *gaetbuli* of gaetbul, the Korean name for a tidal flat).

Basonym: *Erythrobacter gaetbuli* Yoon *et al.* 2005.

The description is the same as for *Erb. gaetbuli* [3]. Core-genomic phylogenetic analysis strongly supported the placement of this species in the genus *Qipengyuania *. The type strain, SW-161^T^ (=KCTC 12227^T^=DSM 16225^T^), was isolated from a tidal flat of the Yellow Sea in the Republic of Korea. The DNA G+C content of the type strain is 64.1 % (by genome).

## Description of *Qipengyuania marisflavi* comb. nov.

*Qipengyuania marisflavi* (ma.ris.fla'vi. L. neut. n. *mare* the sea; L. masc. adj. *flavus* yellow; N.L. gen. n. *marisflavi* of the Yellow Sea).

Basonym: *Erythrobacter marisflavi* Park *et al*. 2019.

The description is the same as for *Erb. marisflavi* [22]. Core-genomic phylogenetic analysis strongly supported the placement of this species in the genus *Qipengyuania *. The type strain, KEM-5^T^ (=KACC 19865^T^=KCTC 62896^T^=NBRC 113546^T^), was isolated from water collected from an estuary environment where the ocean and a river meet at Seocheon, Republic of Korea. The DNA G+C content of the type strain is 61.7 % (by genome).

## Description of *Qipengyuania nanhaisediminis* comb. nov.

*Qipengyuania nanhaisediminis* (nan.hai.se.di'mi.nis. Chin. n. *nanhai* meaning 'the South China Sea'; L. gen. n. *sediminis* of a sediment; N.L. gen. n. *nanhaisediminis* of a sediment from the South China Sea).

Basonym: *Erythrobacter nanhaisediminis* Xu *et al*. 2010.

The description is the same as for *Erb. nanhaisediminis* [113]. Core-genomic phylogenetic analysis strongly supported the placement of this species in the genus *Qipengyuania *. The type strain, T30^T^ (=CGMCC 1.7715^T^=JCM 16125^T^), was isolated from the South China Sea. The DNA G+C content of the type strain is 62.0 % (by genome).

## Description of *Qipengyuania oceanensis* comb. nov.

*Qipengyuania oceanensis* (o.ce.a.nen'sis. L. fem. adj. *oceanensis*, belonging to the ocean).

Basonym: *Altererythrobacter oceanensis* Yang *et al.* 2014.

The description is the same as for *Aeb. oceanensis* [70]. Core-genomic phylogenetic analysis strongly supported the placement of this species in the genus *Qipengyuania *. The type strain, Y2^T^ (=CGMCC 1.12752^T^=LMG 28109^T^), was isolated from a deep-sea sediment of the western Pacific Ocean. The DNA G+C content of the type strain is 63.9 % (by genome).

## Description of *Qipengyuania pelagi* comb. nov.

*Qipengyuania pelagi* (pe'la.gi. L. gen. n. *pelagi* of/from the sea, reflecting isolation of the type strain from seawater of the Red Sea).

Basonym: *Erythrobacter pelagi* Wu *et al*. 2012.

The description is the same as for *Erb. pelagi* [77]. Core-genomic phylogenetic analysis strongly supported the placement of this species in the genus *Qipengyuania *. The type strain, UST081027-248^T^ (=JCM 17468^T^=NRRL 59511^T^), was isolated from shallow seawater collected from the middle of the Red Sea. The DNA G+C content of the type strain is 64.2 % (by genome).

## Emended description of *Qipengyuania sediminis* Feng *et al*. 2015

*Qipengyuania sediminis* (se.di'mi.nis. L. gen. n. *sediminis* of sediment)

The description is identical to that given for *Qpy. sediminis* [2], except for the DNA G+C content. The type strain, M1^T^ (=CGMCC 1.12928^T^=JCM 30182^T^), was isolated from a borehole sediment sample collected from Qiangtang Basin in Qinghai-Tibetan Plateau, PR China. The DNA G+C content of the type strain is 66.7% (by genome).

## Description of *Qipengyuania seohaensis* comb. nov.

*Qipengyuania seohaensis* (seo.ha.en'sis. N.L. fem. adj. *seohaensis* of Seohae, the Korean name of the Yellow Sea in Korea, from where the type strain was isolated).

Basonym: *Erythrobacter seohaensis* Yoon *et al*. 2005.

The description is the same as for *Erb. seohaensis* [3]. Core-genomic phylogenetic analysis strongly supported the placement of this species in the genus *Qipengyuania *. The type strain, SW-135^T^ (=KCTC 12228^T^=DSM 16221^T^=JCM 21815T), was isolated from a tidal flat of the Yellow Sea in the Republic of Korea. The DNA G+C content of the type strain is 61.7 % (by genome).

## Description of *Qipengyuania vulgaris* comb. nov.

*Qipengyuania vulgaris* (vul.ga'ris. L. fem. adj. *vulgaris*, ordinary, usual, common).

Basonym: *Erythrobacter vulgaris* Ivanova *et al*. 2006.

The description is the same as for *Erb. vulgaris* [36]. Core-genomic phylogenetic analysis strongly supported the placement of this species in the genus *Qipengyuania *. The type strain, 022-2-10^T^ (=KMM 3465^T^=CIP 107841^T^=DSM 17792^T^), was isolated from the starfish *Stellaster equestris* collected from the East China Sea. The DNA G+C content of the type strain is 60.6 % (by genome).

## Description of *Alteriqipengyuania* gen. nov.

*Alteriqipengyuania* (Al.te.ri.qi.peng.yu.an'i.a. L. adj. *alter*, another, other, different; N.L. fem. n. *Qipengyuania *, a genus name; N.L. fem. n. *Alteriqipengyuania*, another or different *Qipengyuania *).

Cells are Gram-stain-negative, rod-shaped, non-spore-forming, non-motile and aerobic. Oxidase- and catalase-positive. Contains carotenoid pigments but not bacteriochlorophyll *a*. The predominant ubiquinone is Q-10. The major fatty acids (>10%) are C_17 : 1_ **ω6*c* and summed feature 8 (C_18 : 1_ **ω7*c* and/or C_18 : 1_ **ω6*c*). The genus represents a distinct branch in the family *Erythrobacteraceae* of the class *Alphaproteobacteria* based on the core-genomic phylogeny. The DNA G+C content is 63.6–65.5 ol% (by genome). The type species is *Alteriqipengyuania lutimaris*.

## Description of *Alteriqipengyuania halimionae* comb. nov.

*Alteriqipengyuania halimionae* (ha.li.mi.o'nae. N.L. gen. n. *halimionae* of the marsh plant *Halimione portulacoides*).

Basonym: *Altererythrobacter halimionae* Fidalgo *et al*. 2017.

The description is the same as for *Aeb. halimionae* [32]. Core-genomic phylogenetic analysis strongly supported the placement of this species in the genus *Alteriqipengyuania*. The type strain, CPA5^T^ (=CECT 9130^T^=LMG 29519^T^), was isolated from the surface-sterilized aboveground tissues of the halophyte *Halimione portulacoides*. The DNA G+C content of the type strain is 65.5 % (by genome).

## Description of *Alteriqipengyuania lutimaris* comb. nov.

*Alteriqipengyuania lutimaris* (lu.ti.ma'ris. L. neut. n. *lutum* mud; L. neut. n. *mare* the sea; N.L. gen. n. *lutimaris* of a marine mud).

Basonym: *Erythrobacter lutimaris* Jung *et al.* 2014.

The description is the same as for *Erb. lutimaris* [114]. Core-genomic phylogenetic analysis strongly supported the placement of this species in the genus *Alteriqipengyuania*. The type strain, S-5^T^ (=KCTC 42109^T^=CECT 8624^T^), was isolated from a tidal flat sediment of Saemankum in the Republic ofKorea. The DNA G+C content of the type strain is 63.6 % (by genome).

## Description of *Parerythrobacter* gen. nov.

*Parerythrobacter* (Par.e.ry.thro.bac'ter. Gr. prep. *para*, beside, alongside of, near, like; N.L. masc. n. *Erythrobacter *, a genus name; N.L. masc. n. *Parerythrobacter*, near or like *Erythrobacter *).

Cells are Gram-stain-negative, rod-shaped, non-spore-forming, non-motile and strictly aerobic. Oxidase- and catalase-positive. Contains carotenoid pigments but not bacteriochlorophyll *a*. Requires NaCl for growth. The predominant ubiquinone is Q-10. The major fatty acids (>10%) are C_17 : 1_ **ω6*c* and summed feature 8 (C_18 : 1_ **ω7*c* and/or C_18 : 1_ **ω6*c*). The major polar lipids are diphosphatidylglycerol, phosphatidylcholine, phosphatidylethanolamine, phosphatidylglycerol and sphingoglycolipid. The genus represents a distinct branch in the family *Erythrobacteraceae* of the class *Alphaproteobacteria* based on the core-genomic phylogeny. The DNA G+C content of the type strain is 60.2–60.6 % (by genome). The type species is *Parerythrobacter jejueneis*.

## Description of *Parerythrobacter jejuensis* comb. nov.

*Parerythrobacter jejuensis* (je.ju.en'sis. N.L. masc. adj. *jejuensis* of or belonging to Jeju Island in the Republic of Korea, where the type strain was isolated).

Basonym: *Erythrobacter jejuensis* Yoon *et al*. 2013.

The description is the same as for *Erb. jejuensis* [76]. Core-genomic phylogenetic analysis strongly supported the placement of this species in the genus *Parerythrobacter*. The type strain, CNU001^T^ (=KCTC 23090^T^=JCM 16677^T^), was isolated from seawater collected off Jeju Island, Republic of Korea. The DNA G+C content of the type strain is 60.2 % (by genome).

## Description of *Parerythrobacter lutipelagi* comb. nov.

*Parerythrobacter lutipelagi* (lu.ti.pe.la'gi. L. neut. n. *lutum*, mud; L. neut. n. *pelagus* the sea; N.L. gen. n. *lutipelagi* of mud of the sea, where the type strain was isolated).

Basonym: *Altererythrobacter lutipelagi* Lee 2019.

The description is the same as for *Aeb. lutipelagi* [42]. Core-genomic phylogenetic analysis strongly supported the placement of this species in the genus *Parerythrobacter*. The type strain, GH1-16^T^ (=KCTC 52845^T^=NBRC 113275^T^), was isolated from a tidal mudflat sample collected at the seashore of Gangwha Island, Republic of Korea. The DNA G+C content of the type strain is 60.6 % (by genome).

## Emended description of the genus *Altererythrobacter* Kwon *et al*. 2007, emend. Xue e*t al*. 2012, emend. Xue *et al*. 2016

The description is as given by Kwon *et al*. 2007 [38], Xue *et al.* [15] and Xue *et al*. 2016 [28] with the following amendment. Cells are aerobic and non-motile. Oxidase- and catalase-positive. Requires NaCl for growth. The major fatty acid (>10%) is C_18 : 1_ *ω*7*c*. The major polar lipids are phosphatidylethanolamine, phosphatidylglycerol and sphingoglycolipid. The genus represents a distinct branch in the family *Erythrobacteraceae* of the class *Alphaproteobacteria* based on the core-genomic phylogeny. The DNA G+C content is 52.0–61.8 % (by genome). The type species for the genus is *Altererythrobacter epoxidivorans *.

## Emended description of *Altererythrobacter epoxidivorans* Kwon *et al*. 2007

*Altererythrobacter epoxidivorans* (e.po.xi.di.vo'rans. N.L. neut. n. *epoxidum* epoxide; L. pres. part. *vorans* devouring; N.L. part. adj. *epoxidivorans* epoxide-devouring).

The description is identical to that given for *Aeb. epoxidivorans* [38], except for the DNA G+C content. The type strain, JCS350^T^ (=KCCM 42314^T^=JCM 13815^T^), was isolated from cold-seep sediments of Kagoshima Bay, Japan. The DNA G+C content of the type strain is 61.5 % (by genome).

## Emended description of *Altererythrobacter ishigakiensis* Matsumoto *et al*. 2011

*Altererythrobacter ishigakiensis* (i.shi.ga.ki.en'sis. N.L. masc. adj. *ishigakiensis* of or belonging to Ishigaki island, Okinawa, Japan, where the type strain was isolated).

The description is identical to that given for *Aeb. ishigakiensis* [115], except for the DNA G+C content. The type strain, JPCCMB0017^T^ (=NITE-AP48^T^=ATCC BAA-2084^T^= NBRC 107699^T^), was isolated from the coastal area of Okinawa, Japan. The DNA G+C content of the type strain is 56.9 % (by genome).

## Emended description of *Altererythrobacter xiamenensis* Lei *et al*. 2014

*Altererythrobacter xiamenensis* (xia.men.en'sis. N.L. masc. adj. *xiamenensis* of Xiamen, a city in Fujian, PR China, where the type strain was first isolated).

The description is identical to that given for *Aeb. xiamenensis* [18], except for the DNA G+C content. The type strain, LY02^T^ (=CGMCC 1.12494^T^=KCTC 32398^T^=NBRC 109638^T^), was isolated from red tide seawater in Xiamen, Fujian Province, PR China. The DNA G+C content of the type strain is 61.8 % (by genome).

## Emended description of the genus *Erythrobacter* Shiba *et al*. 1982

The description is as given by Shiba *et al.* 1982 [29] with the following amendment. Cells are motile or non-motile. Positive or negative for oxidase. Requires NaCl for growth. The major fatty acids (>10%) are C_18 : 1_ **ω7*c* and C_17 : 1_ **ω6*c*. The major polar lipids include a sphingoglycolipid. The genus represents a distinct branch in the family *Erythrobacteraceae* of the class *Alphaproteobacteria* based on the core-genomic phylogeny. The DNA G+C content is 57.4–67.9 % (by genome). The type species for the genus is *Erythrobacter longus *.

## Description of *Erythrobacter colymbi* comb. nov.

*Erythrobacter colymbi* (co.lym’bi. L. gen. n. *colymbi*, of a swimming pool, thus indicating the site of isolation of the type strain).

Basomym: *Porphyrobacter colymbi* Rainey *et al*. 2003.

The description is the same as for *Por. colymbi* [24]. Core-genomic phylogenetic analysis strongly supported the placement of this species in the genus *Erythrobacter *. The type strain, TPW-24^T^ (=JCM 18338^T^= KCTC 32078^T^), was isolated from swimming pool water in Tokyo, Japan. The DNA G+C content of the type strain is 66.5 % (by genome).

## Description of *Erythrobacter cryptus* comb. nov.

*Erythrobacter cryptus* (cryp'tus. N.L. masc. adj. *cryptus* from Gr. masc. adj. *kryptos* hidden, to indicate the cryptic relationship of this species to the closely related organisms).

Basomym: *Porphyrobacter cryptus* Rainey *et al*. 2003.

The description is the same as for *Por. cryptus* [26]. Core-genomic phylogenetic analysis strongly supported the placement of this species in the genus *Erythrobacter *. The type strain, ALC-2^T^ (=DSM 12079^T^=ATCC BAA-386^T^), was isolated from the hot spring at Alcafache in Portugal. The DNA G+C content of the type strain is 67.9 % (by genome).

## Description of *Erythrobacter dokdonensis* comb. nov.

*Erythrobacter dokdonensis* (dok.do.nen'sis. N.L. masc. adj. *dokdonensis* of Dokdo, from where the strain was isolated).

Basomym: *Porphyrobacter dokdonensis* Yoon *et al*. 2006.

The description is the same as for *Por. dokdonensis* [106]. Core-genomic phylogenetic analysis strongly supported the placement of this species in the genus *Erythrobacter *. The type strain, DSW-74^T^ (=KCTC 12395^T^=DSM 17193^T^), was isolated from sea water off the island of Dokdo, Korea. The DNA G+C content of the type strain is 64.8 % (by genome).

## Description of *Erythrobacter donghaensis* comb. nov.

*Erythrobacter donghaensis* (dong.ha.en'sis. N.L. masc. adj. *donghaensis* of Donghae, the Korean name for the East Sea in the Republic of Korea from which the strains were isolated).

Basomym: *Porphyrobacter donghaensis* Yoon *et al*. 2004.

The description is the same as for *Por. donghaensis* [105]. Core-genomic phylogenetic analysis strongly supported the placement of this species in the genus *Erythrobacter *. The type strain, SW-132^T^ (=KCTC 12229^T^=DSM 16220^T^), was isolated from sea water from the East Sea in the Republic of Korea. The DNA G+C content of the type strain is 66.2 % (by genome).

## Emended description of *Erythrobacter litoralis* Yurkov *et al*. 1994

*Erythrobacter litoralis* (li.to.ra'lis. L. masc. adj. *litoralis*, at the beach or coast, referring to the supralitoral habitat).

The description is identical to that given for *Erb. litoralis* [31], except for the DNA G+C content. The type strain, T4^T^ (=ATCC 700002^T^=CIP 106926^T^=DSM 8509^T^=JCM 10281^T^=NBRC 102620^T^), was isolated from a marine cyanobacterial mat in a supralitoral zone. The DNA G+C content of the type strain is 65.2 % (by genome).

## Emended description of *Erythrobacter longus* Shiba *et al*. 1982

*Erythrobacter longus* (lon'gus. L. masc. adj. *longus*, long).

The description is identical to that given for *Erb. longus* [29], except for the DNA G+C content. The type strain, Och01^T^ (=ATCC 33941^T^=CIP 104268^T^=DSM 6997^T^= JCM 6170^T^=NBRC 14126^T^), was isolated from high-tidal seaweed *Enteromorpha linza*. The DNA G+C content of the type strain is 57.4 % (by genome).

## Description of *Erythrobacter neustonensis* comb. nov.

*Erythrobacter neustonensis* [neu.sto.nen'sis. N.L. masc. adj. derived from Gr. n. *neustos*, swimming (floating), referring to occurrence of the bacterium as a member of the neuston (organisms floating at the air–water interface surface layer of a body of water)].

Basomym: *Porphyrobacter neustonensis* Fuerst *et al*. 1993.

The description is the same as for *Por. neustonensis* [23]. Core-genomic phylogenetic analysis strongly supported the placement of this species in the genus *Erythrobacter *. The type strain, ACM 2844^T^ (=CIP 104070^T^=DSM 9434^T^), was isolated from air–water interface of freshwater subtropical pond in Brisbane, Australia. The DNA G+C content of the type strain is 65.3 % (by genome).

## Description of *Erythrobacter ramosus* comb. nov.

*Erythrobacter ramosus* (ra.mo'sus. L. masc. adj. *ramosus*, ramifying, referring to the morphology of the cells).

Basomym: *Erythromicrobium ramosum* Yurkov *et al*. 1994.

The description is the same as for *Erm. ramosum* [31]. Core-genomic phylogenetic analysis strongly supported the placement of this species in the genus *Erythrobacter *. The type strain, E5^T^ (=ATCC 700003^T^=CIP 106927^T^=DSM 8510^T^=JCM 10282^T^=NBRC 102621^T^), was isolated from a cyanobacterial mat from an alkaline spring. The DNA G+C content of the type strain is 64.3 % (by genome).

## Description of *Erythrobacter sanguineus* comb. nov.

*Erythrobacter sanguineus* (san.gui'ne.us. L. masc. adj. *sanguineus* blood-coloured).

Basomym: *Porphyrobacter sanguineus* Hiraishi *et al*. 2002.

The description is the same as for *Por. sanguineus* [104]. Core-genomic phylogenetic analysis strongly supported the placement of this species in the genus *Erythrobacter *. The type strain, A91^T^ (=ATCC 25659^T^=DSM 11302^T^=IAM 12620^T^=ICPB 4167^T^=NBRC 15763^T^=JCM 20691^T^), was isolated from sea water collected in Baltic Sea. The DNA G+C content of the type strain is 63.6 % (by genome).

## Description of *Erythrobacter tepidarius* comb. nov.

*Erythrobacter tepidarius* (te.pi.da’ri.us. L. neut. n. *tepidarium*, a warm bath fed by natural thermal water; N.L. masc. adj. *tepidarius*, warm bathing).

Basomym: *Porphyrobacter tepidarius* Hanada *et al*. 1997.

The description is the same as for *Por. tepidarius* [27]. Core-genomic phylogenetic analysis strongly supported the placement of this species in the genus *Erythrobacter *. The type strain, OT3^T^ (=DSM 10594^T^), was isolated from a cyanobacterial mat in brackish water of a hot spring in Shidzuoka Prefecture, Japan. The DNA G+C content of the type strain is 65.9 % (by genome).

## Description of *Pontixanthobacter* gen. nov.

*Pontixanthobacter* (Pon.ti.xan.tho.bac'ter. L. masc. n. *pontus*, the sea; Gr. masc. adj. *xanthos*, yellow; N.L. masc. n. *bacter*, rod or staff; N.L. masc. n. *Pontixanthobacter*, a yellow bacterium from the sea).

Cells are Gram-stain-negative, ovoid to rod, non-spore-forming, non-motile and aerobic. Positive and negative for oxidase. Catalase-positive. Contains carotenoid pigments but not bacteriochlorophyll *a*. The predominant ubiquinone is Q-10. The major fatty acid (>10 %) is summed feature 8 (C_18 : 1_ **ω7*c* and/or C_18 : 1_ **ω6*c*). The major polar lipids are phosphatidylcholine, phosphatidylethanolamine, phosphatidylglycerol and sphingoglycolipid. The genus represents a distinct branch in the family *Erythrobacteraceae* of the class *Alphaproteobacteria* based on the core-genomic phylogeny. The DNA G+C content is 55.5–61.5 % (by genome). The type species is *Pontixanthobacter luteolus*.

## Description of *Pontixanthobacter aestiaquae* comb. nov.

*Pontixanthobacter aestiaquae* (aes.ti.a'quae. L. masc. n. *aestus* the sea tide; L. fem. n. *aqua* water; N.L. gen. n. *aestiaquae* of the water of the sea tide).

Basonym: *Altererythrobacter aestiaquae* Jung *et al*. 2014.

The description is the same as for *Aeb. aestiaquae* [62]. Core-genomic phylogenetic analysis strongly supported the placement of this species in the genus *Pontixanthobacter*. The type strain, HDW-31^T^ (=KCTC 42006^T^=CECT 8527^T^), was isolated from seawater of Hwang-do in the Republic of Korea. The DNA G+C content of the type strain is 57.2 % (by genome).

## Description of *Pontixanthobacter aquaemixtae* comb. nov.

*Pontixanthobacter aquaemixtae* (a.quae.mi'xtae. L. fem. n. *aqua* water; L. fem. perf. part. *mixta* mixed; N.L. fem. gen. n. *aquaemixtae* of mixed waters).

Basonym: *Altererythrobacter aquaemixtae* Park *et al*. 2017.

The description is the same as for *Aeb. aquaemixtae* [64]. Core-genomic phylogenetic analysis strongly supported the placement of this species in the genus *Pontixanthobacter*. The type strain, JSSK-8^T^ (=KCTC 52763^T^=NBRC 112764^T^), was isolated from the place where the ocean and a freshwater spring meet at Jeju Island, Republic of Korea. The DNA G+C content of the type strain is 58.5 % (by genome).

## Description of *Pontixanthobacter confluentis* comb. nov.

*Pontixanthobacter confluentis* (con.flu.en'tis. L. gen. n. *confluentis* of a meeting place of waters).

Basonym: *Altererythrobacter confluentis* Park *et al*. 2016.

The description is the same as for *Aeb. confluentis* [20]. Core-genomic phylogenetic analysis strongly supported the placement of this species in the genus *Pontixanthobacter*. The type strain, KEM-4^T^ (=KCTC 52259^T^=NBRC 112305^T^), was isolated from water collected from an estuary environment where the ocean and a river meet at Seocheon, Republic of Korea. The DNA G+C content of the type strain is 59.1 % (by genome).

## Description of *Pontixanthobacter gangjinensis* comb. nov.

*Pontixanthobacter gangjinensis* (gang.jin.en'sis. N.L. masc. adj. *gangjinensis* pertaining to Gangjin bay where the type strain was isolated).

Basonym: *Altererythrobacter gangjinensis* Jeong *et al*. 2013.

The description is the same as for *Aeb. gangjinensis* [67]. Core-genomic phylogenetic analysis strongly supported the placement of this species in the genus *Pontixanthobacter*. The type strain, KJ7^T^ (=KACC 16190^T^=JCM 17802^T^), was isolated from a tidal flat of the Gangjin bay in the Republic of Korea. The DNA G+C content of the type strain is 55.5 % (by genome).

## Description of *Pontixanthobacter luteolus* comb. nov.

*Pontixanthobacter luteolus* (lu.te'o.lus. L. masc. adj. *luteolus*, yellowish).

Basonym: *Altererythrobacter luteolus* Yoon *et al*. 2005. emend. Kwon *et al*. 2007

The description is the same as for *Aeb. luteolus* [38, 68]. Core-genomic phylogenetic analysis strongly supported the placement of this species in the genus *Pontixanthobacter*. The type strain, SW-109^T^ (=KCTC 12311^T^=JCM 12599^T^), was isolated from a tidal flat of the Yellow Sea in the Republic of Korea. The DNA G+C content of the type strain is 59.3 % (by genome).

## Description of *Pontixanthobacter sediminis* comb. nov.

*Pontixanthobacter sediminis* (se.di'mi.nis. L. gen. n. *sediminis* of sediment).

Basonym: *Altererythrobacter sediminis* Kim *et al*. 2016.

The description is the same as for *Aeb. sediminis* [72]. Core-genomic phylogenetic analysis strongly supported the placement of this species in the genus *Pontixanthobacter*. The type strain, CAU 1172^T^ (=KCTC 42453^T^=NBRC 110917^T^), was isolated from a sample of lagoon sediment from along the east coast of the Republic of Korea. The DNA G+C content of the type strain is 61.5 % (by genome).

## Description of *Alteripontixanthobacter* gen. nov.

*Alteripontixanthobacter* (Al.te.ri.pon.ti.xan.tho.bac'ter. L. adj. *alter*, another, other, different; N.L. masc. n. *Pontixanthobacter*, a genus name; N.L. masc. n. *Alteripontixanthobacter*, another or different *Pontixanthobacter*).

Cells are Gram-stain-negative, rod, non-spore-forming, non-motile and aerobic. Oxidase- and catalase-positive. Contains carotenoid pigments but not bacteriochlorophyll *a*. Requires NaCl for growth. The predominant ubiquinone is Q-10. The major fatty acids (>10%) are summed feature 8 (C_18 : 1_ **ω7*c* and/or C_18 : 1_ **ω6*c*), summed feature 3 (C_16 : 1_ **ω7*c* and/or C_16 : 1_ **ω6*c*) and C_16 : 0_. The major polar lipids are diphosphatidylglycerol, phosphatidylcholine, phosphatidylethanolamine, phosphatidylglycerol and sphingoglycolipid. The genus represents a distinct branch in the family *Erythrobacteraceae* of the class *Alphaproteobacteria* based on the core-genomic phylogeny. The DNA G+C content is 60.8 % (by genome). The type species is *Alteripontixanthobacter maritimus*.

## Description of *Alteripontixanthobacter maritimus* comb. nov.

*Alteripontixanthobacter maritimus* (ma.ri'ti.mus. L. masc. adj. *maritimus* of the marine environment).

Basonym: *Altererythrobacter maritimus* Kang *et al*. 2019.

The description is the same as for *Aeb. maritimus* [43]. Core-genomic phylogenetic analysis strongly supported the placement of this species in the genus *Alteripontixanthobacter*. The type strain, HME9302^T^ (=KCTC 32463^T^=KACC 17617^T^=CECT 8417^T^), was isolated from seawater in the Republic of Korea. The DNA G+C content of the type strain is 60.8 % (by genome).

## Description of *Parapontixanthobacter* gen. nov.

*Parapontixanthobacter* (Pa.ra.pon.ti.xan.tho.bac'ter. Gr. prep. *para*, beside, alongside of, near, like; N.L. masc. n. *Pontixanthobacter*, a genus name; N.L. masc. n. *Parapontixanthobacter*, near or like *Pontixanthobacter*).

Cells are Gram-stain-negative, coccoid, non-spore-forming, non-motile and strictly aerobic. Oxidase-negative and catalase-positive. Contains carotenoid pigments but not bacteriochlorophyll *a*. Requires NaCl for growth. Reduces nitrate to nitrite. The predominant ubiquinone is Q-10. The major fatty acids (>10 %) are summed feature 8 (C_18 : 1_ **ω7*c* and/or C_18 : 1_ **ω6*c*), summed feature 3 (C_16 : 1_ **ω7*c* and/or C_16 : 1_ **ω6*c*) and C_16 : 0_. The major polar lipids are diphosphatidylglycerol, phosphatidylethanolamine, phosphatidylglycerol and sphingoglycolipid. The genus represents a distinct branch in the family *Erythrobacteraceae* of the class *Alphaproteobacteria* based on the core-genomic phylogeny. The DNA G+C content is 61.2 % (by genome).The type species is *Parapontixanthobacter aurantiacus*.

## Description of *Parapontixanthobacter aurantiacus* comb. nov.

*Parapontixanthobacter aurantiacus* (au.ran.ti'a.cus. N.L. masc. adj. *aurantiacus*, orange-coloured).

Basonym: *Altererythrobacter aurantiacus* Zhang *et al*. 2016.

The description is the same as for *Aeb. aurantiacus* [65]. Core-genomic phylogenetic analysis strongly supported the placement of this species in the genus *Parapontixanthobacter*. The type strain, O30^T^ (=CGMCC 1.12762^T^=JCM 19853^T^=LMG 28110^T^=MCCC 1A09962^T^), was isolated from a deep-sea sediment of the west Pacific Ocean. The DNA G+C content of the type strain is 61.2 % (by genome).

## Description of *Pelagerythrobacter* gen. nov.

*Pelagerythrobacter* (Pe.lag.e.ry.th.ro.bac'ter. L. neut. n. *pelagus* the sea; N.L. masc. n. *Erythrobacter *, a genus name; N.L. masc. n. *Pelagerythrobacter*, *Erythrobacter* from the sea).

Cells are Gram-stain-negative, rod-shaped, non-spore-forming and aerobic. Motile or non-motile. Positive and negative for oxidase. Catalase-positive. Contains carotenoid pigments but not bacteriochlorophyll *a*. Requires NaCl for growth. The predominant ubiquinone is Q-10. The major fatty acid (>10 %) is C_18 : 1_ **ω7*c*. The major polar lipids are diphosphatidylglycerol, phosphatidylcholine, phosphatidylethanolamine, phosphatidylglycerol and sphingoglycolipid. The genus represents a distinct branch in the family *Erythrobacteraceae* of the class *Alphaproteobacteria* based on the core-genomic phylogeny. The DNA G+C content is 64.7–68.2 % (by genome).The type species is *Pelagerythrobacter marinus*.

## Description of *Pelagerythrobacter aerophilus* comb. nov.

*Pelagerythrobacter aerophilus* (a.e.ro'phi.lus. Gr. masc. n. *aer*, air; N.L. adj. *philus* from Gr. masc. adj. *philos* friend, loving; N.L. masc. adj. *aerophilus*, air-loving).

Basonym: *Altererythrobacter aerophilus* Meng *et al*. 2019.

The description is the same as for *Aeb. aerophilus* [17]. Core-genomic phylogenetic analysis strongly supported the placement of this species in the genus *Pelagerythrobacter*. The type strain, Ery1^T^ (=KCTC 62387^T^=CGMCC 1.16499^T^=MCCC 1A10037^T^), was isolated from deep-sea seawater of the Mariana Trench. The DNA G+C content of the type strain is 65.4 % (by genome).

## Description of *Pelagerythrobacter marinus* comb. nov.

*Pelagerythrobacter marinus* (ma.ri'nus. L. masc. adj. *marinus* of the sea, marine).

Basonym: *Altererythrobacter marinus* Lai *et al*. 2009.

The description is the same as for *Aeb. marinus* [69]. Core-genomic phylogenetic analysis strongly supported the placement of this species in the genus *Pelagerythrobacter*. The type strain, H32^T^ (=CCTCC AB 208229^T^=LMG 24629^T^=MCCC 1A01070^T^), was isolated from deep seawater of the Indian Ocean. The DNA G+C content of the type strain is 68.2 % (by genome).

## Description of *Pelagerythrobacter marensis* comb. nov.

*Pelagerythrobacter marensis* (ma.ren'sis. N.L. masc. adj. *marensis* of Mara Island, Jeju, Republic of Korea, where the type strain was isolated).

Basonym: *Altererythrobacter marensis* Seo *et al*. 2010.

The description is the same as for *Aeb. marensis* [116]. Core-genomic phylogenetic analysis strongly supported the placement of this species in the genus *Pelagerythrobacter*. The type strain, MSW-14^T^ (=KCTC 22370^T^=DSM 21428^T^), was isolated from seawater collected around Mara Island, Jeju, Republic of Korea. The DNA G+C content of the type strain is 64.7 % (by genome).

## Description of *Altericroceibacterium* gen. nov.

*Altericroceibacterium* (Al.te.ri.cro.ce.i.bac.te'ri.um. L. masc. adj. *alter* another, other, different; N.L. neut. n. *Croceibacterium *, a genus name; N.L. neut. n. *Altericroceibacterium*, another or different *Croceibacterium *).

Cells are Gram-stain-negative, rod-shaped, non-spore-forming, aerobic and non-motile. Positive and negative for oxidase. Catalase-positive. Contains carotenoid pigments but not bacteriochlorophyll *a*. Requires NaCl for growth. The predominant ubiquinone is Q-10. The major fatty acid (>10%) is summed feature 8 (C_18 : 1_ **ω7*c* and/or C_18 : 1_ **ω6*c*). The major polar lipid is phosphatidylethanolamine. The genus represents a distinct branch in the family *Erythrobacteraceae* of the class *Alphaproteobacteria* based on the core-genomic phylogeny. The DNA G+C content is 55.8–64.2 % (by genome). The type species is *Altericroceibacterium indicum*.

## Description of *Altericroceibacterium endophyticum* comb. nov.

*Altericroceibacterium endophyticum* (en.do.phy'ti.cum. Gr. pref. *endo* within; Gr. n. *phyton* plant; L. neut. suff. -*icum* adjectival suffix used with the sense of belonging to; N.L. neut. adj. *endophyticum* within plant, endophytic).

Basonym: *Altererythrobacter endophyticus* Fidalgo *et al*. 2017.

The description is the same as for *Aeb. endophyticus* [32]. Core-genomic phylogenetic analysis strongly supported the placement of this species in the genus *Altericroceibacterium*. The type strain, BR75^T^ (=CECT 9129^T^=LMG 29518^T^), was isolated from the surface-sterilized belowground tissues of the halophyte *Halimione portulacoides*. The DNA G+C content of the type strain is 58.6 % (by genome).

## Description of *Altericroceibacterium indicum* comb. nov.

*Altericroceibacterium indicum* (in'di.cum. L. neut. adj. *indicum* pertaining to India, where the type strain was isolated).

Basonym: *Altererythrobacter indicus* Kumar *et al*. 2008.

The description is the same as for *Aeb. indicus* [33]. Core-genomic phylogenetic analysis strongly supported the placement of this species in the genus *Altericroceibacterium*. The type strain, MSSRF26^T^ (=LMG 23789^T^=DSM 18604^T^), isolated from the rhizosphere of mangrove-associated wild rice (*Porteresia coarctata* Tateoka). The DNA G+C content of the type strain is 55.8 % (by genome).

## Description of *Altericroceibacterium xinjiangense* comb. nov.

*Altericroceibacterium xinjiangense* (xin.jiang.en'se. N.L. neut. adj. *xinjiangense* of or pertaining to Xinjiang, an autonomous region in north-west China).

Basonym: *Altererythrobacter xinjiangensis* Xue *et al*. 2012.

The description is the same as for *Aeb. xinjiangensis* [15]. Core-genomic phylogenetic analysis strongly supported the placement of this species in the genus *Altericroceibacterium*. The type strain, S3-63^T^ (=CCTCC AB 207166^T^=CIP 110125^T^), was isolated from sand from the desert of Xinjiang, PR China. The DNA G+C content of the type strain is 64.2 % (by genome).

## Emended description of the genus *Croceibacterium* Liu *et al*. 2019

The description is as given by Liu *et al.* [30]with the following amendment. Cells are pleomorphic. Some species can motile by means of polar flagella. Positive or negative for oxidase. The major fatty acid (>10 %) is summed feature 8 (C_18 : 1_ **ω7*c* and/or C_18 : 1_ **ω6*c*). The major polar lipids are diphosphatidylglycerol, phosphatidylethanolamine and phosphatidylglycerol. The genus represents a distinct branch in the family *Erythrobacteraceae* of the class *Alphaproteobacteria* based on the core-genomic phylogeny. The DNA G+C content is 61.9–67.0 % (by genome). The type species for the genus is *Croceibacterium ferulae *.

## Description of *Croceibacterium atlanticum* comb. nov.

*Croceibacterium atlanticum* (at.lan'ti.cum. L. neut. adj. *atlanticum* of or pertaining to the Atlantic Ocean, where the type strain was isolated).

Basonym: *Altererythrobacter atlanticus* Wu *et al*. 2014.

The description is the same as for *Aeb. atlanticus* [8]. Core-genomic phylogenetic analysis strongly supported the placement of this species in the genus *Croceibacterium *. The type strain, 26DY36^T^ (=CGMCC 1.12411^T^=JCM 18865^T^), was isolated from a deep-sea sediment sample collected from the North Atlantic Rise. The DNA G+C content of the type strain is 61.9 % (by genome).

## Description of *Croceibacterium salegens* comb. nov.

*Croceibacterium salegens* (sal.e'gens. L. masc. n. *sal*, *salis* salt; L. pres. part. *egens* needy; N.L. part. adj. *salegens* salt-needy).

Basonym: *Altererythrobacter salegens* Liang *et al*. 2017.

The description is the same as for *Aeb. salegens* [71]. Core-genomic phylogenetic analysis strongly supported the placement of this species in the genus *Croceibacterium *. The type strain, XY-R17^T^ (=KCTC 52267^T^=MCCC 1K01500^T^), was isolated from the surface sediment of Mai Po Inner Deep Bay Ramsar Site in Hong Kong. The DNA G+C content of the type strain is 64.6% (by genome).

## Description of *Croceibacterium soli* comb. nov.

*Croceibacterium soli* (so'li. L. gen. n. *soli* of soil).

Basonym: *Altererythrobacter soli* Zhao *et al.* 2017.

The description is the same as for *Aeb. soli* [14]. Core-genomic phylogenetic analysis strongly supported the placement of this species in the genus *Croceibacterium *. The type strain, MN-1^T^ (=KCTC 52135^T^=MCCC 1K02066^T^), isolated from a desert sand sample collected from Tengger desert, north-western PR China. The DNA G+C content of the type strain is 67.0 % (by genome).

## Description of *Croceibacterium xixiisoli* comb. nov.

*Croceibacterium xixiisoli* (xi.xi.i.so'li. L. gen. n. *soli* of soil. N.L. gen. n. *xixiisoli* from Xixi soil).

Basonym: *Altererythrobacter xixiisoli* Yuan *et al*. 2017.

The description is the same as for *Aeb. xixiisoli* [12]. Core-genomic phylogenetic analysis strongly supported the placement of this species in the genus *Croceibacterium *. The type strain, S36^T^ (=CGMCC 1.12804^T^=NBRC 110413^T^), was isolated from soil of the Xixi wetland in Hangzhou, eastern PR China. The DNA G+C content of the type strain is 63.3 % (by genome).

## Description of *Alteraurantiacibacter* gen. nov.

*Alteraurantiacibacter* (Al.ter.au.ran.ti.a.ci.bac'ter. L. masc. adj. *alter* another, other, different; N.L. masc. n. *Aurantiacibacter*, a genus name; N.L. masc. n. *Alteraurantiacibacter*, another or different *Aurantiacibacter*).

Cells are Gram-stain-negative, pleomorphic, non-spore-forming, aerobic and non-motile. Oxidase- and catalase-positive. Contains carotenoid pigments but not bacteriochlorophyll *a*. Requires NaCl for growth. The predominant ubiquinone is Q-10. The major fatty acid (>10%) is summed feature 8 (C_18 : 1_ **ω7*c* and/or C_18 : 1_ **ω6*c*). The major polar lipids are phosphatidylethanolamine and phosphatidylglycerol. The genus represents a distinct branch in the family *Erythrobacteraceae* of the class *Alphaproteobacteria* based on the core-genomic phylogeny. The DNA G+C content is 62.5–66.0 % (by genome). The type species is *Alteraurantiacibacter aestuarii*.

## Description of *Alteraurantiacibacter aestuarii* comb. nov.

*Alteraurantiacibacter aestuarii* (aes.tu.a'ri.i. L. gen. n. *aestuarii* of a tidal flat, from where the type strain was isolated).

Basonym: *Altererythrobacter aestuarii* Park *et al*. 2011.

The description is the same as for *Aeb. aestuarii* [63]. Core-genomic phylogenetic analysis strongly supported the placement of this species in the genus *Alteraurantiacibacter*. The type strain, KYW147^T^ (=KCTC 22735^T^=JCM 16339^T^), was isolated from a seawater sample collected from the South Sea, Republic of Korea. The DNA G+C content of the type strain is 62.5 % (by genome).

## Description of *Alteraurantiacibacter aquimixticola* comb. nov.

*Alteraurantiacibacter aquimixticola* (a.qui.mix.ti’co.la. L. fem. n. *aqua* water; L. masc. perf. part. *mixtus* mixed; L. suff. -*cola* inhabitant; N.L. masc. n. *aquimixticola* an inhabitant of mixed waters).

Basonym: *Altererythrobacter aquimixticola* Park *et al*. 2019.

The description is the same as for *Aeb. aquimixticola* [40]. Core-genomic phylogenetic analysis strongly supported the placement of this species in the genus *Alteraurantiacibacter*. The type strain, SSKS-13^T^ (=KACC 19863^T^=KCTC 62900^T^=NBRC 113545^T^), was isolated from sediment sampled at the junction between the ocean and a freshwater spring at Jeju island of the South Sea, Republic of Korea. The DNA G+C content of the type strain is 63.9 % (by genome).

## Description of *Alteraurantiacibacter buctensis* comb. nov.

*Alteraurantiacibacter buctensis* (buc.ten'sis. N.L. masc. adj. *buctensis* referring to the acronym BUCT, Beijing University of Chemical Technology, where the strain was identified).

Basonym: *Altererythrobacter buctensis* Zhang *et al*. 2016.

The description is the same as for *Aeb. buctensis* [66]. Core-genomic phylogenetic analysis strongly supported the placement of this species in the genus *Alteraurantiacibacter*. The type strain, M0322^T^ (=CGMCC 1.12871^T^=JCM 30112^T^), was isolated from the Mohe Basin, PR China. The DNA G+C content of the type strain is 66.0 % (by genome).

## Description of *Aurantiacibacter* gen. nov

*Aurantiacibacter* (Au.ran.ti.a.ci.bac'ter. N.L. masc. adj. *aurantiacus*, orange-coloured; N.L. masc. n. *bacter*, rod or staff; N.L. masc. n. *Aurantiacibacter*, orange-coloured rod).

Cells are Gram-stain-negative, pleomorphic and non-spore-forming. Aerobic or facultative anaerobic. Positive or negative for oxidase. Catalase-positive. Contains carotenoid pigments but not bacteriochlorophyll *a*. The predominant ubiquinone is Q-10. The major fatty acid (>10 %) is summed feature 8 (C_18 : 1_ **ω7*c* and/or C_18 : 1_ **ω6*c*). The major polar lipid is phosphatidylethanolamine. The genus represents a distinct branch in the family *Erythrobacteraceae* of the class *Alphaproteobacteria* based on the core-genomic phylogeny. The DNA G+C content is 58.3–67.2 % (by genome). The type species is *Aurantiacibacter gangjinensis*.

## Description of *Aurantiacibacter aquimixticola* comb. nov.

*Aurantiacibacter aquimixticola* (a.qui.mix.ti’co.la. L. fem. n. *aqua* water; L. masc. perf. part. *mixtus* mixed; L. suff. -*cola* from L. n. *incola* dweller, inhabitant; N.L. masc. n. *aquimixticola* an inhabitant of mixed waters).

Basonym: *Erythrobacter aquimixticola* Park *et al*. 2017.

The description is the same as for *Erb. aquimixticola* [88]. Core-genomic phylogenetic analysis strongly supported the placement of this species in the genus *Aurantiacibacter*. The type strain, JSSK-14^T^ (=KCTC 52764^T^=NBRC 112765^T^), was isolated from water from the place where the ocean and a freshwater spring meet at Jeju island, Republic of Korea. The DNA G+C content of the type strain is 63.0 % (by genome).

## Description of *Aurantiacibacter arachoides* comb. nov.

*Aurantiacibacter arachoides* (a.ra.cho'i.des. N.L. fem. n. *arachis*, peanut; L. suff. –*oides*, looking like; N.L. masc. adj. *arachoides*, looking like a peanut).

Basonym: *Erythrobacter arachoides* Xing *et al*. 2017.

The description is the same as for *Erb. arachoides* [74]. Core-genomic phylogenetic analysis strongly supported the placement of this species in the genus *Aurantiacibacter*. The type strain, RC4-10-4^T^ (=CGMCC 1.15507^T^=JCM 31277^T^), was isolated from an ice core in the East Rongbuk Glacier, Tibetan Plateau. The DNA G+C content of the type strain is 65.4 % (by genome).

## Description of *Aurantiacibacter atlanticus* comb. nov.

*Aurantiacibacter atlanticus* (at.lan'ti.cus. N.L. masc. adj. *atlanticus* referring to the Atlantic Ocean, where the type strain was isolated).

Basonym: *Erythrobacter atlanticus* Zhuang *et al*. 2015.

The description is the same as for *Erb. atlanticus* [109]. Core-genomic phylogenetic analysis strongly supported the placement of this species in the genus *Aurantiacibacter*. The type strain, s21-N3^T^ (=MCCC 1A00519^T^=KCTC 42697^T^) was isolated from deep-sea sediment of the Atlantic Ocean. The DNA G+C content of the type strain is 58.3 % (by genome).

## Description of *Aurantiacibacter gangjinensis* comb. nov.

*Aurantiacibacter gangjinensis* (gang.jin.en'sis. N.L. masc. adj. *gangjinensis* referring to Gangjin, the name of the bay in Korea from which the type strain was isolated).

Basonym: *Erythrobacter gangjinensis* Lee *et al*. 2010.

The description is the same as for *Erb. gangjinensis* [117]. Core-genomic phylogenetic analysis strongly supported the placement of this species in the genus *Aurantiacibacter*. The type strain, K7-2^T^ (=KCTC 22330^T^=JCM 15420^T^), was isolated from seawater of Gangjin Bay, Republic of Korea. The DNA G+C content of the type strain is 62.7 % (by genome).

## Description of *Aurantiacibacter luteus* comb. nov.

*Aurantiacibacter luteus* (lu'te.us. L. masc. adj. *luteus* orange-coloured, referring to the colour of the colony).

Basonym: *Erythrobacter luteus* Lei *et al.* 2015.

The description is the same as for *Erb. luteus* [118]. Core-genomic phylogenetic analysis strongly supported the placement of this species in the genus *Aurantiacibacter*. The type strain, KA37^T^ (=MCCC 1F01227^T^=KCTC 42179^T^), was isolated from a mangrove sediment sample collected from Yunxiao mangrove National Nature Reserve, Fujian Province, PR China. The DNA G+C content of the type strain is 67.2 % (by genome).

## Description of *Aurantiacibacter marinus* comb. nov.

*Aurantiacibacter marinus* (ma.ri'nus. L. masc. adj. *marinus* of the sea, marine).

Basonym: *Erythrobacter marinus* Jung *et al*. 2012.

The description is the same as for *Erb. marinus* [119]. Core-genomic phylogenetic analysis strongly supported the placement of this species in the genus *Aurantiacibacter*. The type strain, HWDM-33^T^ (=KCTC 23554^T^=CCUG 60528^T^), was isolated from seawater of Hwang-do, an island of the Yellow Sea, Republic of Korea. The DNA G+C content of the type strain is 59.1 % (by genome).

## Description of *Aurantiacibacter odishensis* comb. nov.

*Aurantiacibacter odishensis* (o.dish.en'sis. N.L. masc. adj. *odishensis* of or belonging to Odisha, a coastal state in India rich in bacterial diversity).

Basonym: *Erythrobacter odishensis* Subhash *et al*. 2013.

The description is the same as for *Erb. odishensis* [13]. Core-genomic phylogenetic analysis strongly supported the placement of this species in the genus *Aurantiacibacter*. The type strain, JA747^T^ (=KCTC 23981^T^=NBRC 108930^T^), was isolated from a soil sample of a solar saltern at Humma, Odisha, India. The DNA G+C content of the type strain is 63.7 % (by genome).

## Description of *Aurantiacibacter spongiae* comb. nov.

*Aurantiacibacter spongiae* (spon'gi.ae. L. gen. n. *spongiae* of a sponge, the source of the type strain).

Basonym: *Erythrobacter spongiae* Zhuang *et al*. 2019.

The description is the same as for *Erb. spongiae* [35]. Core-genomic phylogenetic analysis strongly supported the placement of this species in the genus *Aurantiacibacter*. The type strain, HN-E23^T^ (=MCCC 1K03331^T^=LMG 30457^T^), was isolated from a sponge sample. The DNA G+C content of the type strain is 65.5 % (by genome).

## Description of *Aurantiacibacter xanthus* comb. nov.

*Aurantiacibacter xanthus* (xan'thus. Gr. masc. adj. *xanthos* yellow; N.L. masc. adj. *xanthus* yellow).

Basonym: *Erythrobacter xanthus* Li *et al*. 2017.

The description is the same as for *Erb. xanthus* [19]. Core-genomic phylogenetic analysis strongly supported the placement of this species in the genus *Aurantiacibacter*. The type strain, SM1501^T^ (=KCTC 42669^T^=CCTCC AB 2015396^T^), was isolated from surface seawater of the South China Sea. The DNA G+C content of the type strain is 64.5 % (by genome).

## Description of *Aurantiacibacter zhengii* comb. nov.

*Aurantiacibacter zhengii* [zheng'i.i. N.L. gen. n. *zhengii* of TianLing Zheng (June 1955 to August 2017), a Chinese microbiologist, for his contributions to marine microorganism resources, biological control of harmful red tides, and our understanding of the interactions between bacteria and algae].

Basonym: *Erythrobacter zhengii* Fang *et al*. 2019.

The description is the same as for *Erb. zhengii* [9]. Core-genomic phylogenetic analysis strongly supported the placement of this species in the genus *Aurantiacibacter*. The type strain, V18^T^ (=KCTC 62389^T^=MCCC 1K03475^T^), was isolated from deep-sea sediment of the Pacific Ocean. The DNA G+C content of the type strain is 62.7 % (by genome).

## Description of *Paraurantiacibacter* gen. nov.

*Paraurantiacibacter* (Par.au.ran.ti.a.ci.bac'ter. Gr. prep. *para*, beside, alongside of, near, like; N.L. masc. n. *Aurantiacibacter*, a genus name; N.L. masc. n. *Paraurantiacibacter*, near or like *Aurantiacibacter*).

Cells are Gram-stain-negative, ovoid- to rod-shaped, non-spore-forming and aerobic. Oxidase- and catalase-positive. Requires NaCl for growth. Contains carotenoid pigments but not bacteriochlorophyll *a*. The predominant ubiquinone is Q-10. The major fatty acids (>10 %) are C_18 : 1_ **ω7*c* and summed feature 3 (C_16 : 1_ **ω7*c* and/or C_16 : 1_ **ω6*c*). The major polar lipids are diphosphatidylglycerol, phosphatidylcholine, phosphatidylethanolamine, phosphatidylglycerol and sphingoglycolipid. The genus represents a distinct branch in the family *Erythrobacteraceae* of the class *Alphaproteobacteria* based on the core-genomic phylogeny. The DNA G+C content is 65.0 % (by genome). The type species is *Paraurantiacibacter namhicola*.

## Description of *Paraurantiacibacter namhicola* comb. nov.

*Paraurantiacibacter namhicola* (nam.hi'co.la. N.L. n. *namhae* Namhae, the Korean name of the South Sea; L. suff. -*cola* from L. n. *incola* a dweller, inhabitant; N.L. masc. n. *namhicola* a dweller of the South Sea, referring to the isolation of the type strain).

Basonym: *Altererythrobacter namhicola* Park *et al*. 2011.

The description is the same as for *Aeb. namhicola* [63]. Core-genomic phylogenetic analysis strongly supported the placement of this species in the genus *Paraurantiacibacter*. The type strain, KYW48^T^ (=KCTC 22736^T^=JCM 16345^T^), was isolated from a seawater sample collected from the South Sea, Republic of Korea. The DNA G+C content of the type strain is 65.0 % (by genome).

## Emended description of the genus *Croceicoccus* Xu *et al*. 2009 emend. Huang *et al*. 2015

The description is as given by Xu *et al*. [4] and Huang *et al*. [110] with the following amendments. Cells are coccid or rods. The major fatty acid (>10 %) is C_18 : 1_ **ω7*c*. The genus represents a distinct branch in the family *Erythrobacteraceae* of the class *Alphaproteobacteria* based on the core-genomic phylogeny. The DNA G+C content is 62.5–64.5 % (by genome). The type species for the genus is *Croceicoccus marinus *.

## Emended description of *Croceicoccus marinus* Xu *et al*. 2009 emend. Huang *et al*. 2015

*Croceicoccus marinus* (ma.ri'nus. L. masc. adj. *marinus* of or belonging to the sea, marine)

The description is identical to that given for *Ccc. marinus* [4, 110], except for the DNA G+C content. The type strain, E4A9^T^ (=CGMCC 1.6776^T^=JCM 14846^T^), was isolated from a deep-sea sediment sample collected from a polymetallic nodule region in the East Pacific Ocean. The DNA G+C content of the type strain is 64.5% (by genome).

## Emended description of *Croceicoccus naphthovorans* Huang *et al.* 2015

*Croceicoccus naphthovorans* (naph.tho.vo'rans. Gr. fem. n. *naphtha* oil; L. pres. part. *vorans* devouring; N.L. part. adj. *naphthovorans* oil-degrading).

The description is identical to that given for *Ccc. naphthovorans* [110], except for the DNA G+C content. The type strain, PQ-2^T^ (=CGMCC 1.12805^T^=NBRC 110381^T^) was isolated from marine biofilm collected from a boat shell at a harbour of Zhoushan island in Zhejiang Province, PR China. The DNA G+C content of the type strain is 62.6 % (by genome).

## Supplementary Data

Supplementary material 1
