# Supplementary material for: Genomic-based taxonomic classification of the family Erythrobacteraceae
Source: Int J Syst Evol Microbiol. 2020 Jul 29;70(8):4470–95. doi: 10.1099/ijsem.0.004293 (PMC7660246; doi:10.1099/ijsem.0.004293)
Supplement: Supplementary material 1 [file ijsem-70-4470-s001.pdf]

# Supplementary material

## Genomic-based taxonomic classification of the family *Erythrobacteraceae*

Lin Xu[1,2], Cong Sun[1,2], Chen Fang[1,3], Aharon Oren[4] and Xue-Wei Xu[1,5]

[1] Key Laboratory of Marine Ecosystem Dynamics, Ministry of Natural Resources & Second Institute of Oceanography, Ministry of Natural Resources, Hangzhou 310012, PR China

[2] College of Life Sciences and Medicine, Zhejiang Sci-Tech University, Hangzhou 310018, PR China

[3] College of Oceanography, Hohai University, Nanjing 210000, PR China

[4] The Institute of Life Sciences, The Hebrew University of Jerusalem, Edmond J. Safra Campus, Jerusalem 9190401, Israel

[5] School of Oceanography, Shanghai Jiao Tong University, Shanghai 200030, PR China

*Corresponding author: 36th North Baochu Road, Ministry of Natural Resources & Second Institute of Oceanography, Ministry of Natural Resources, Hangzhou 310012, PR China (X.-W. Xu)

Email address: xuxw@sio.org.cn (X.-W. Xu)

Table S1. Currently validly-published genera and species belonging to the family *Erythrobacteraceae*.

| Genus and species | Type strain | 16S rRNA gene accession number | Source | Reference |
|---|---|---|---|---|
| *Altererythrobacter* | | | | |
| *Aeb. aerius* | 100921-2$^{T}$ | KU311004. | Air | [1] |
| *Aeb. aerophilus* | Ery1$^{T}$ | MG183676 | Seawater | [2] |
| *Aeb. aestiaquae* | HDW-31$^{T}$ | KJ658262 | Seawater | [3] |
| *Aeb. aestuarii* | KYW147$^{T}$ | FJ997597 | Seawater | [4] |
| *Aeb. amylolyticus* | NS1$^{T}$ | KX601069 | Lake sediment | [5] |
| *Aeb. aquaemixtae* | JSSK-8$^{T}$ | KY614064 | Ocean and freshwater spring junction water | [6] |
| *Aeb. aquiaggeris* | KEM-3$^{T}$ | KX812543 | Estuary water | [7] |
| *Aeb. aquimixticola* | SSKS-13$^{T}$ | MK194299 | Ocean and freshwater spring junction sediment | [8] |
| *Aeb. atlanticus* | 26DY36$^{T}$ | KC018454 | Marine sediment | [9] |
| *Aeb. aurantiacus* | O30$^{T}$ | KF924607 | Marine sediment | [10] |
| *Aeb. buctensis* | M0322$^{T}$ | KJ599648 | Mudstone core | [11] |
| *Aeb. confluentis* | KEM-4$^{T}$ | KX129915 | Estuary water | [12] |
| *Aeb. deserti* | THG-S3$^{T}$ | KY287245 | Desert sand | [13] |
| *Aeb. dongtanensis* | JM27$^{T}$ | GU166344 | Tidal flat | [14] |
| *Aeb. endophyticus* | BR75$^{T}$ | KY310591 | Salt marsh plant | [15] |
| *Aeb. epoxidivorans* | JCS350$^{T}$ | DQ304436 | Marine sediment | [16] |
| *Aeb. flavus* | MS1-4$^{T}$ | KX099616 | Mangrove sediment | [17] |
| *Aeb. fulvus* | S-54$^{T}$ | KY117470 | Soil | [18] |
| *Aeb. gangjinensis* | KJ7$^{T}$ | JF751048 | Tidal flat | [19] |
| *Aeb. halimionae* | CPA5$^{T}$ | KY310593 | Salt marsh plant | [15] |

| Genus and species | Type strain | 16S rRNA gene accession number | Source | Reference |
|---|---|---|---|---|
| *Aeb. indicus* | MSSRF26$^{T}$ | DQ399262 | Wild rice | [20] |
| *Aeb. insulae* | BPTF-M16$^{T}$ | MH206217 | Tidal flat | [21] |
| *Aeb. ishigakiensis* | JPCCMB0017$^{T}$ | AB363004 | Marine sediment | [22] |
| *Aeb. lauratis* | YIM 75003$^{T}$ | KX808673 | Hot spring | [23] |
| *Aeb. luteolus* | SW-109$^{T}$ | AY739662 | Tidal flat | [24] |
| *Aeb. lutipelagi* | GH1-16$^{T}$ | LT797153 | Tidal flat | [25] |
| *Aeb. mangrovi* | C9-11$^{T}$ | MF034045 | Mangrove sediment | [26] |
| *Aeb. marensis* | MSW-14$^{T}$ | FM177586 | Seawater | [27] |
| *Aeb. marinus* | H32$^{T}$ | EU726272 | Seawater | [28] |
| *Aeb. maritimus* | HME9302$^{T}$ | KF385494 | Seawater | [29] |
| *Aeb. namhicola* | KYW48$^{T}$ | FJ935793 | Seawater | [4] |
| *Aeb. oceanensis* | Y2$^{T}$ | KF924606 | Marine sediment | [30] |
| *Aeb. palmitatis* | YIM 75004$^{T}$ | KX808674 | Hot spring | [23] |
| *Aeb. rigui* | WW3$^{T}$ | KP997219 | Freshwater | [31] |
| *Aeb. salegens* | XY-R17$^{T}$ | KT886062 | Marine sediment | [32] |
| *Aeb. sediminis* | CAU1172$^{T}$ | KP779619 | Marine sediment | [33] |
| *Aeb. soli* | MN-1$^{T}$ | KT906300 | Desert sand | [34] |
| *Aeb. troitsensis* | KMM 6042$^{T}$ | AY676115 | Sea urchin | [35] |
| *Aeb. xiamenensis* | LY02$^{T}$ | KC520828 | Seawater | [36] |
| *Aeb. xinjiangensis* | S3-63$^{T}$ | HM028673 | Desert sand | [37] |
| *Aeb. xixiisoli* | S36$^{T}$ | KJ150597 | Soil | [38] |
| *Croceibacterium* | | | | |

| Genus and species | Type strain | 16S rRNA gene accession number | Source | Reference |
|---|---|---|---|---|
| *Crb. ferulae* | SX2RGS8$^T$ | KX289944 | Medicinal plant | [39] |
| *Crb. mercuriale* | Coronado$^T$ | KP122961 | Stadium seat | [40] |
| *Croceicoccus* | | | | |
| *Ccc. marinus* | E4A9$^T$ | EF623998 | Marine sediment | [41] |
| *Ccc. mobilis* | Ery22$^T$ | KT383846 | Marine sediment | [42] |
| *Ccc. naphthovorans* | PQ-2$^T$ | KF145127 | Marine biofilm | [43] |
| *Ccc. pelagius* | Ery9$^T$ | KT383844 | Marine sediment | [42] |
| *Erythrobacter* | | | | |
| *Erb. aquimaris* | SW-110$^T$ | AY461441 | Tidal flat | [44] |
| *Erb. aquimixticola* | JSSK-14$^T$ | KY614066 | Ocean and freshwater spring junction water | [45] |
| *Erb. arachoides* | RC4-10-4$^T$ | KU302715 | Glacier | [46] |
| *Erb. atlanticus* | s31-N3$^T$ | KP994305 | Marine sediment | [47] |
| *Erb. citreus* | RE35F/1$^T$ | AF118020 | Seawater | [48] |
| *Erb. flavus* | SW-46$^T$ | AF500004 | Seawater | [49] |
| *Erb. gaetbuli* | SW-161$^T$ | AY562220 | Intertidal sediment | [50] |
| *Erb. gangjinensis* | K7-2$^T$ | EU428782 | Seawater | [51] |
| *Erb. jejuensis* | CNU001$^T$ | DQ453142 | Seawater | [52] |
| *Erb. litoralis* | T4$^T$ | AF465836 | Marine cyanobacterial mat | [53] |
| *Erb. longus* | OCh101$^T$ | AF465835 | Seaweed | [54] |
| *Erb. luteus* | KA37$^T$ | KP197673 | Mangrove sediment | [55] |
| *Erb. lutimaris* | S-5$^T$ | KJ870094 | Intertidal sediment | [56] |
| *Erb. marinus* | HWDM-33$^T$ | HQ117934 | Seawater | [57] |

| Genus and species | Type strain | 16S rRNA gene accession number | Source | Reference |
|---|---|---|---|---|
| *Erb. marisflavi* | KEM-5$^{T}$ | MK121890 | Estuary water | [58] |
| *Erb. nanhaisediminis* | T30$^{T}$ | FJ654473 | Marine sediment | [59] |
| *Erb. odishensis* | JA747$^{T}$ | HE680094 | Saltern soil | [60] |
| *Erb. pelagi* | UST081027-248$^{T}$ | HQ203045 | Seawater | [61] |
| *Erb. seohaensis* | SW-135$^{T}$ | AY562219 | Intertidal sediment | [50] |
| *Erb. spongiae* | HN-E23$^{T}$ | MG655147 | Marine sponge | [62] |
| *Erb. vulgaris* | 022-2-10$^{T}$ | AY706935 | Marine starfish | [63] |
| *Erb. xanthus* | SM1501$^{T}$ | KU529278 | Seawater | [64] |
| *Erb. zhengii* | V18$^{T}$ | MG183678 | Marine sediment | [65] |
| *Erythromicrobium* | | | | |
| *Erm. ramosum* | E5$^{T}$ | AF465837 | Alkaline spring cyanobacterial spring | [53] |
| *Porphyrobacter* | | | | |
| *Por. algicida* | 2-22$^{T}$ | KU981071 | Seawater | [66] |
| *Por. colymbi* | TPW-24$^{T}$ | AB702992 | Swimming pool water | [67] |
| *Por. cryptus* | ALC-2$^{T}$ | AF465834 | Hot spring | [68] |
| *Por. dokdonensis* | DSW-74$^{T}$ | DQ011529 | Seawater | [69] |
| *Por. donghaensis* | SW-132$^{T}$ | AY559428 | Seawater | [70] |
| *Por. neustonensis* | ACM 2844$^{T}$ | AB033327 | Freshwater | [71] |
| *Por. sanguineus* | A91$^{T}$ | AB021493 | Seawater | [72] |
| *Por. tepidarius* | OT3$^{T}$ | AB033328 | Hot spring | [73] |
| *Qipengyuania* | | | | |
| *Qpy. sediminis* | M1$^{T}$ | KJ734993 | Subterrestrial sediment | [74] |

Table S2. Genomic completeness and contamination as well as rRNA and tRNA gene counts of obtained *Erythrobacteraceae* genomes.

| Genomes | Genomic quality | | Gene counts | |
|---|---|---|---|---|
| | Completeness (%) | Contamination (%) | rRNA gene | tRNA gene |
| *Aeb. aerius* 100921-2$^T$ | 99.4 | 0.1 | 3 | 48 |
| *Aeb. aerophilus* Ery1$^T$ | 99.6 | 4.9 | 3 | 45 |
| *Aeb. aestiaquae* KCTC 42006$^T$ | 98.5 | 0.2 | 3 | 42 |
| *Aeb. aestuarii* JCM 16339$^T$ | 99.1 | 0.4 | 3 | 45 |
| *Aeb. amylolyticus* NS1$^T$ | 99.5 | 0.3 | 3 | 45 |
| *Aeb. aquaemixtae* KCTC 52763$^T$ | 99.0 | 1.1 | 3 | 45 |
| *Aeb. aquimixticola* SSKS-13$^T$ | 98.8 | 0.2 | 3 | 45 |
| *Aeb. atlanticus* 26DY36$^T$ | 99.5 | 1.8 | 6 | 47 |
| *Aeb. aurantiacus* MCCC 1A09962$^T$ | 98.7 | 0.3 | 3 | 41 |
| *Aeb. buctensis* M0322$^T$ | 98.9 | 0.5 | 3 | 47 |
| *Aeb. confluentis* KCTC 52259$^T$ | 99.4 | 0.1 | 6 | 46 |
| *Aeb. dongtanensis* KCTC 22672$^T$ | 99.5 | 0.2 | 3 | 47 |
| *Aeb. endophyticus* LMG 29518$^T$ | 99.3 | 0.3 | 3 | 45 |
| *Aeb. epoxidivorans* CGMCC 1.7731$^T$ | 99.2 | 1.3 | 3 | 43 |
| *Aeb. flavus* MS1-4$^T$ | 99.1 | 1.0 | 4 | 46 |
| *Aeb. gangjinensis* JCM 17802$^T$ | 99.2 | 0.1 | 3 | 42 |
| *Aeb. halimionae* LMG 29519$^T$ | 99.2 | 0.5 | 3 | 44 |
| *Aeb. indicus* DSM 18604$^T$ | 99.4 | 0.9 | 17 | 54 |
| *Aeb. insulae* BPTF-M16$^T$ | 98.4 | 2.8 | 9 | 47 |
| *Aeb. ishigakiensis* NBRC 107699$^T$ | 98.9 | 0.7 | 3 | 42 |
| *Aeb. luteolus* SW-109$^T$ | 99.9 | 0.3 | 12 | 52 |

| Genomes | Genomic quality | | Gene counts | |
|---|---|---|---|---|
| | Completeness (%) | Contamination (%) | rRNA gene | tRNA gene |
| *Aeb. lutipelagi* GH1-16$^T$ | 99.6 | 0.6 | 3 | 44 |
| *Aeb. mangrovi* C9-11$^T$ | 99.0 | 0.8 | 3 | 46 |
| *Aeb. marensis* KCTC 22370$^T$ | 99.5 | 0.6 | 3 | 45 |
| *Aeb. marinus* H32$^T$ | 99.4 | 0.3 | 3 | 46 |
| *Aeb. maritimus* HME9302$^T$ | 99.2 | 1.7 | 3 | 43 |
| *Aeb. namhicola* JCM 16345$^T$ | 99.5 | 0 | 3 | 44 |
| *Aeb. oceanensis* MCCC 1A09965$^T$ | 99.2 | 0.2 | 3 | 45 |
| *Aeb. rigui* KCTC 42620$^T$ | 99.3 | 0 | 3 | 49 |
| *Aeb. salegens* MCCC 1K01500$^T$ | 99.3 | 0.7 | 3 | 47 |
| *Aeb. sediminis* KCTC 42453$^T$ | 99.2 | 0.2 | 3 | 42 |
| *Aeb. soli* MCCC 1K02066$^T$ | 99.6 | 0 | 3 | 48 |
| *Aeb. troitsensis* JCM 17037$^T$ | 99.6 | 0.2 | 3 | 45 |
| *Aeb. xiamenensis* CGMCC 1.12494$^T$ | 99.1 | 0.3 | 3 | 44 |
| *Aeb. xinjiangensis* CCTCC AB 207166$^T$ | 97.8 | 0.7 | 3 | 47 |
| *Aeb. xixiisoli* S36$^T$ | 99.4 | 2.4 | 3 | 48 |
| *Crb. ferulae* SX2RGS8$^T$ | 97.6 | 1.1 | 3 | 51 |
| *Crb. mercuriale* Coronado$^T$ | 98.1 | 0.4 | 3 | 48 |
| *Ccc. marinus* E4A9$^T$ | 99.6 | 1.8 | 6 | 48 |
| *Ccc. mobilis* Ery22$^T$ | 99.4 | 2.7 | 12 | 54 |
| *Ccc. naphthovorans* PQ-2$^T$ | 99.5 | 0.3 | 3 | 45 |
| *Ccc. pelagius* Ery9$^T$ | 99.3 | 0.5 | 9 | 51 |
| *Erb. aquimaris* JCM 12189$^T$ | 99.6 | 0 | 3 | 45 |

| Genomes | Genomic quality | | Gene counts | |
|---|---|---|---|---|
| | Completeness (%) | Contamination (%) | rRNA gene | tRNA gene |
| *Erb. aquimixticola* JSSK-14$^T$ | 99.2 | 1.2 | 3 | 45 |
| *Erb. arachoides* RC4-10-4$^T$ | 99.5 | 0.4 | 3 | 46 |
| *Erb. atlanticus* s21-N3$^T$ | 99.1 | 1.1 | 3 | 45 |
| *Erb. citreus* CGMCC 1.8703$^T$ | 99.8 | 0.4 | 12 | 52 |
| *Erb. gaetbuli* DSM 16225$^T$ | 99.8 | 0.3 | 3 | 45 |
| *Erb. gangjinensis* CGMCC 1.15024$^T$ | 99.2 | 0.6 | 3 | 43 |
| *Erb. jejuensis* JCM 16677$^T$ | 98.3 | 4.8 | 6 | 61 |
| *Erb. litoralis* DSM 8509$^T$ | 99.2 | 0.9 | 3 | 45 |
| *Erb. longus* DSM 6997$^T$ | 99.6 | 0.6 | 3 | 42 |
| *Erb. luteus* KA37$^T$ | 99.5 | 0 | 3 | 45 |
| *Erb. lutimaris* S-5$^T$ | 99.5 | 0.3 | 11 | 44 |
| *Erb. marinus* KCTC 23554$^T$ | 99.2 | 0.5 | 3 | 43 |
| *Erb. marisflavi* KEM-5$^T$ | 99.9 | 0 | 3 | 43 |
| *Erb. nanhaisediminis* CGMCC 1.7715$^T$ | 99.8 | 0.1 | 3 | 44 |
| *Erb. odishensis* KCTC 23981$^T$ | 99.6 | 0.3 | 3 | 45 |
| *Erb. pelagi* JCM 17468$^T$ | 99.5 | 0 | 3 | 44 |
| *Erb. seohaensis* SW-135$^T$ | 99.8 | 0.1 | 3 | 44 |
| *Erb. spongiae* HN-E23$^T$ | 99.8 | 0.5 | 3 | 45 |
| *Erb. vulgaris* DSM 17792$^T$ | 98.9 | 0 | 3 | 45 |
| *Erb. xanthus* CCTCC AB 2015396$^T$ | 99.2 | 0.5 | 12 | 55 |
| *Erb. zhengii* V18$^T$ | 99.9 | 3.0 | 3 | 46 |
| *Erm. ramosum* JCM 10282$^T$ | 99.5 | 1.1 | 3 | 44 |

| Genomes | Genomic quality | | Gene counts | |
|---|---|---|---|---|
| | Completeness (%) | Contamination (%) | rRNA gene | tRNA gene |
| *Por. algicida* KEMB 9005-328$^{T}$ | 99.9 | 1.0 | 3 | 47 |
| *Por. colymbi* JCM 18338$^{T}$ | 99.3 | 0.9 | 3 | 44 |
| *Por. cryptus* DSM 12079$^{T}$ | 99.5 | 0 | 3 | 44 |
| *Por. dokdonensis* DSM 17193$^{T}$ | 99.3 | 0.8 | 3 | 44 |
| *Por. donghaensis* DSM 16220$^{T}$ | 99.5 | 1.0 | 3 | 44 |
| *Por. neustonensis* DSM 9434$^{T}$ | 99.5 | 0.3 | 6 | 47 |
| *Por. sanguineus* JCM 20691$^{T}$ | 99.6 | 0.7 | 3 | 44 |
| *Por. tepidarius* DSM 10594$^{T}$ | 99.7 | 0 | 3 | 44 |
| *Qpy. sediminis* CGMCC 1.12928$^{T}$ | 99.5 | 0.2 | 3 | 44 |

Table S3. Sequence identities of annotated 16S rRNA genes within sequenced *Erythrobacteraceae* genomes in this study through the EzBioCloud web server. Red indicates that the type strain has multi-copy 16S rRNA genes.

| Genome | Closest type strain | Identity (%) |
|---|---|---|
| *Aeb. aerius* 100921-2$^T$ | *Aeb. aerius* 100921-2$^T$ | 99.2 |
| *Aeb. aestiaquae* KCTC 42006$^T$ | *Aeb. aestiaquae* HDW-31$^T$ | 100.0 |
| *Aeb. aestuarii* JCM 16339$^T$ | *Aeb. aestuarii* KYW147$^T$ | 100.0 |
| *Aeb. aquaemixtae* KCTC 52763$^T$ | *Aeb. aquaemixtae* JSSK-8$^T$ | 100.0 |
| *Aeb. aurantiacus* MCCC 1A09962$^T$ | *Aeb. aurantiacus* O30$^T$ | 99.9 |
| *Aeb. buctensis* M0322$^T$ | *Aeb. buctensis* M0322$^T$ | 100.0 |
| *Aeb. confluentis* KCTC 52259$^T$ | *Aeb. confluentis* KEM-4$^T$ | 100.0 |
| | *Aeb. confluentis* KEM-4$^T$ | 100.0 |
| *Aeb. endophyticus* LMG 29518$^T$ | *Aeb. endophyticus* BR75$^T$ | 100.0 |
| *Aeb. gangjinensis* JCM 17802$^T$ | *Aeb. gangjinensis* KJ7$^T$ | 100.0 |
| *Aeb. halimionae* LMG 29519$^T$ | *Aeb. halimionae* CPA5$^T$ | 100.0 |
| *Aeb. indicus* DSM 18604$^T$ | *Aeb. indicus* MSSRF26$^T$ | 100.0 |
| | *Aeb. indicus* MSSRF26$^T$ | 100.0 |
| | *Aeb. indicus* MSSRF26$^T$ | 100.0 |
| | *Aeb. indicus* MSSRF26$^T$ | 100.0 |
| | *Aeb. indicus* MSSRF26$^T$ | 100.0 |
| *Aeb. luteolus* SW-109$^T$ | *Aeb. luteolus* SW-109$^T$ | 100.0 |
| | *Aeb. luteolus* SW-109$^T$ | 100.0 |
| | *Aeb. luteolus* SW-109$^T$ | 100.0 |
| | *Aeb. luteolus* SW-109$^T$ | 100.0 |
| *Aeb. marinus* H32$^T$ | *Aeb. marinus* H32$^T$ | 100.0 |

| Genome | Closest type strain | Identity (%) |
|---|---|---|
| *Aeb. oceanensis* MCCC 1A09965$^{T}$ | *Aeb. oceanensis* Y2$^{T}$ | 100.0 |
| *Aeb. salegens* MCCC 1K01500$^{T}$ | *Aeb. salegens* XY-R17$^{T}$ | 100.0 |
| *Aeb. sediminis* KCTC 42453$^{T}$ | *Aeb. sediminis* CAU1172$^{T}$ | 100.0 |
| *Aeb. soli* MCCC 1K02066$^{T}$ | *Aeb. soli* MN-1$^{T}$ | 100.0 |
| *Aeb. xixiisoli* S36$^{T}$ | *Aeb. xixiisoli* S36$^{T}$ | 99.9 |
| *Erb. aquimaris* JCM 12189$^{T}$ | *Erb. aquimaris* SW-110$^{T}$ | 100.0 |
| *Erb. arachoides* RC4-10-4$^{T}$ | *Erb. arachoides* RC4-10-4$^{T}$ | 99.3 |
| *Erb. citreus* CGMCC 1.8703$^{T}$ | *Erb. citreus* RE35F/1$^{T}$<br>*Erb. citreus* RE35F/1$^{T}$<br>*Erb. citreus* RE35F/1$^{T}$<br>*Erb. citreus* RE35F/1$^{T}$ | 100.0<br>100.0<br>100.0<br>100.0 |
| *Erb. gaetbuli* DSM 16225$^{T}$ | *Erb. gaetbuli* SW-161$^{T}$ | 100.0 |
| *Erb. jejuensis* JCM 16677$^{T}$ | *Erb. jejuensis* CNU001$^{T}$<br>*Erb. jejuensis* CNU001$^{T}$ | 99.6<br>99.6 |
| *Erb. pelagi* JCM 17468$^{T}$ | *Erb. pelagi* JCM 17468$^{T}$ | 99.8 |
| *Erb. vulgaris* DSM 17792$^{T}$ | *Erb. vulgaris* 022 2-10$^{T}$ | 100.0 |
| *Erm. ramosum* JCM 10282$^{T}$ | *Erm. ramosum* DSM 8510$^{T}$ | 100.0 |
| *Por. algicida* KEMB 9005-328$^{T}$ | *Por. algicida* KEMB 9005-328$^{T}$ | 100.0 |

Table S4. Annotations of OCs for the phylogenomic analysis assigned to COG and KEGG databases and their best substitutional model.

| Group | Annotation | COG | KEGG | Best substitution model | |
|---|---|---|---|---|---|
| | | | | Nucleotides | Amino acids |
| 1 | SSU ribosomal protein S4p (S9e) | J | Ribosome | GTR+F+R5 | LG+R4 |
| 2 | Queuine tRNA-ribosyltransferase | J | Transfer RNA biogenesis | GTR+F+R6 | LG+F+R5 |
| 3 | S-adenosylmethionine:tRNA ribosyltransferase-isomerase | J | Transfer RNA biogenesis | TIM3+F+R6 | LG+F+R5 |
| 4 | Peptidyl-prolyl cis-trans isomerase | O | Necroptosis | GTR+F+R5 | LG+F+I+G4 |
| 5 | Phosphopantetheine adenylyltransferase | H | Pantothenate and CoA biosynthesis | TVM+F+R5 | LG+R5 |
| 6 | (2E,6E)-farnesyl diphosphate synthase | H | Terpenoid backbone biosynthesis | TIM3+F+R5 | LG+F+R5 |
| 7 | Exodeoxyribonuclease VII small subunit | L | DNA repair and recombination proteins<br>Mismatch repair | TIM3+F+R5 | Dayhoff+I+G4 |
| 8 | Phosphoribosylformylglycinamidine synthase, synthetase subunit | F | Nucleotide metabolism | GTR+F+R6 | LG+F+R5 |
| 9 | Phosphoserine phosphatase | E | Amino acid metabolism<br>Energy metabolism | TIM3+F+R6 | LG+F+R6 |
| 10 | tRNA dimethylallyltransferase | J | Biosynthesis of secondary metabolites<br>Transfer RNA biogenesis | TVM+F+R6 | LG+F+I+G4 |
| 11 | Ketol-acid reductoisomerase (NADP(+)) | E | Amino acid metabolism<br>Metabolism of cofactors and vitamins | GTR+F+R5 | LG+R5 |
| 12 | Phosphate transport ATP-binding protein PstB | P | ABC transporters | GTR+F+R5 | LG+R5 |
| 13 | Phosphate transport system regulatory protein PhoU | P | Signaling proteins | GTR+F+R5 | LG+R4 |
| 14 | Phosphate regulon transcriptional regulatory protein PhoB | T | Signaling proteins | GTR+F+R5 | LG+R5 |
| 15 | Inositol-1-monophosphatase | G | Inositol phosphate metabolism<br>Phosphatidylinositol signaling system<br>Streptomycin biosynthesis | GTR+F+R6 | LG+R4 |

| Group | Annotation | COG | KEGG | Best substitution model | |
|---|---|---|---|---|---|
| | | | | Nucleotides | Amino acids |
| 16 | Translation elongation factor P | J | Translation factors | GTR+F+R5 | LG+R3 |
| 17 | Thiamine-phosphate pyrophosphorylase | H | Thiamine metabolism | GTR+F+I+G4 | LG+F+R5 |
| 18 | Transketolase | G | Pentose phosphate pathway | GTR+F+R6 | LG+F+R6 |
| 19 | ATP-dependent DNA helicase RecG | L | Homologous recombination | GTR+F+R6 | LG+F+R5 |
| 20 | Transcription-repair coupling factor | L | Nucleotide excision repair | GTR+F+R6 | LG+F+R5 |
| 21 | NAD kinase | G | Nicotinate and nicotinamide metabolism | GTR+F+R5 | LG+I+G4 |
| 22 | Protein translocase subunit SecA | U | Bacterial secretion system<br>Quorum sensing | GTR+F+R6 | LG+F+R6 |
| 23 | Glutamate N-acetyltransferase (EC 2.3.1.35) | E | Arginine biosynthesis | TIM3+F+R6 | LG+F+R5 |
| 24 | Thioredoxin | O | Chaperones and folding catalysts | GTR+F+I+G4 | LG+G4 |
| 25 | Nucleotidyl transferase | M | Fructose and mannose metabolism | GTR+F+R6 | LG+R5 |
| 26 | Adenosylhomocysteinase | H | Cysteine and methionine metabolism | GTR+F+R6 | LG+F+R5 |
| 27 | Peroxiredoxin | O | Apoptosis | GTR+F+I+G4 | LG+I+G4 |
| 28 | Excinuclease ABC subunit C | L | Nucleotide excision repair | GTR+F+R6 | LG+F+R5 |
| 29 | Translation initiation factor 1 | J | Translation factors | TIM2+F+R5 | LG+R2 |
| 30 | Septum formation protein Maf | D | Signaling proteins | GTR+F+R6 | LG+F+R6 |
| 31 | Phosphoribosylaminoimidazole-succinocarboxamide synthase | F | Purine metabolism | GTR+F+I+G4 | LG+R5 |
| 32 | Glutamine-fructose-6-phosphate aminotransferase | M | Alanine, aspartate and glutamate metabolism<br>Amino sugar and nucleotide sugar metabolism | GTR+F+R6 | LG+F+R6 |
| 33 | Type II restriction adenine-specific methylase | L | Prokaryotic defense system | GTR+F+R6 | LG+R6 |
| 34 | Ribonuclease HII | L | DNA replication | GTR+F+R5 | LG+F+R5 |
| 35 | Topoisomerase IV subunit A | L | DNA replication | GTR+F+R5 | LG+F+R5 |

| Group | Annotation | COG | KEGG | Best substitution model | |
|---|---|---|---|---|---|
| | | | | Nucleotides | Amino acids |
| | | | DNA repair and recombination | | |
| 36 | Ferredoxin, 2Fe-2S | C | Energy metabolism | TIM3+F+R4 | WAG+I+G4 |
| 37 | Hydrolase SMc00528, alpha/beta fold family | S | Function unknown | TIM+F+R5 | LG+R4 |
| 38 | DNA-binding response regulator ChvI | T | Signaling proteins | GTR+F+R5 | LG+R6 |
| 39 | PTS system permease (IIAMan), nitrogen regulatory IIA protein | G | Galactose metabolism<br>Phosphotransferase system | GTR+F+I+G4 | LG+I+G4 |
| 40 | Phosphocarrier protein, nitrogen regulation associated | G | Phosphotransferase system | TPM3u+F+I+G4 | LG+I+G4 |
| 41 | Recombination protein RecR | L | Homologous recombination | TIM3+F+R5 | LG+R5 |
| 42 | Methionyl-tRNA formyltransferase | J | Aminoacyl-tRNA biosynthesis<br>One carbon pool by folate | GTR+F+R5 | LG+F+I+G4 |
| 43 | tRNA pseudouridine(38-40) synthase | J | Transfer RNA biogenesis | TVM+F+R5 | WAG+F+R5 |
| 44 | Argininosuccinate lyase | E | Alanine, aspartate and glutamate metabolism | GTR+F+R6 | LG+F+R6 |
| 45 | Diaminopimelate decarboxylase | E | Lysine biosynthesis | GTR+F+R6 | LG+F+R5 |
| 46 | Adenylosuccinate synthetase | F | Purine metabolism | GTR+F+R6 | LG+R5 |
| 47 | Replication-associated recombination protein RarA | L | DNA replication and repair | GTR+F+R6 | LG+F+R6 |
| 48 | Argininosuccinate synthase | E | Alanine, aspartate and glutamate metabolism | GTR+F+R5 | WAG+R4 |
| 49 | Excinuclease ABC subunit A | L | Nucleotide excision repair | GTR+F+R6 | LG+R6 |
| 50 | Malate dehydrogenase | C | Carbon fixation pathways in prokaryotes<br>Citrate cycle<br>Cysteine and methionine metabolism<br>Glyoxylate and dicarboxylate metabolism<br>Pyruvate metabolism | TIM+F+R5 | LG+F+R4 |

| Group | Annotation | COG | KEGG | Best substitution model | |
|---|---|---|---|---|---|
| | | | | Nucleotides | Amino acids |
| 51 | 2-oxoglutarate dehydrogenase E1 component | C | Citrate cycle | GTR+F+R6 | LG+R5 |
| 52 | E2 component of 2-oxoglutarate dehydrogenase complex | C | Citrate cycle<br>Glycolysis / Gluconeogenesis<br>Pyruvate metabolism | GTR+F+R6 | LG+F+R5 |
| 53 | LSU ribosomal protein L17p | J | Ribosome | TIM+F+I+G4 | LG+I+G4 |
| 54 | DNA-directed RNA polymerase alpha subunit | K | RNA polymerase | GTR+F+R5 | LG+R4 |
| 55 | SSU ribosomal protein S11p | L | Ribosome | TIM2+F+I+G4 | LG+R2 |
| 56 | SSU ribosomal protein S13p | J | Ribosome | TIM2+F+I+G4 | LG+I+G4 |
| 57 | Protein translocase subunit SecY | U | Bacterial secretion system<br>Quorum sensing | GTR+F+R6 | LG+F+R5 |
| 58 | LSU ribosomal protein L15p | J | Ribosome | TIM3+F+I+G4 | WAG+I+G4 |
| 59 | LSU ribosomal protein L30p | J | Ribosome | GTR+F+I+G4 | LG+R3 |
| 60 | SSU ribosomal protein S5p | J | Ribosome | TIM3+F+R5 | WAG+F+R4 |
| 61 | LSU ribosomal protein L18p | J | Ribosome | TN+F+I+G4 | WAG+I+R4 |
| 62 | LSU ribosomal protein L6p | J | Ribosome | TIM+F+R5 | LG+I+G4 |
| 63 | SSU ribosomal protein S8p | J | Ribosome | TIM+F+R5 | LG+R3 |
| 64 | SSU ribosomal protein S14p | J | Ribosome | TIM2+F+I+G4 | LG+R2 |
| 65 | LSU ribosomal protein L5p | J | Ribosome | GTR+F+R5 | LG+R4 |
| 66 | LSU ribosomal protein L24p | J | Ribosome | TIM2+F+R4 | LG+G4 |
| 67 | LSU ribosomal protein L14p | J | Ribosome | TIM2+F+I+G4 | LG+I+G4 |
| 68 | SSU ribosomal protein S17p | J | Ribosome | TIM+F+I+G4 | WAG+G4 |
| 69 | LSU ribosomal protein L29p | J | Ribosome | TIM2+F+I+G4 | LG+I+G4 |

| Group | Annotation | COG | KEGG | Best substitution model | |
|---|---|---|---|---|---|
| | | | | Nucleotides | Amino acids |
| 70 | LSU ribosomal protein L16 | J | Ribosome | TIM2+F+R4 | LG+R3 |
| 71 | SSU ribosomal protein S3p | J | Ribosome | TIM2+F+R5 | LG+R4 |
| 72 | LSU ribosomal protein L22p | J | Ribosome | GTR+F+R5 | LG+I+G4 |
| 73 | SSU ribosomal protein S19p | J | Ribosome | GTR+F+R4 | JTTDCMut+I+G4 |
| 74 | LSU ribosomal protein L2p | J | Ribosome | GTR+F+R5 | LG+R4 |
| 75 | LSU ribosomal protein L23p | J | Ribosome | GTR+F+I+G4 | LG+I+G4 |
| 76 | LSU ribosomal protein L4p | J | Ribosome | GTR+F+R5 | LG+I+G4 |
| 77 | LSU ribosomal protein L3p | J | Ribosome | TIM2+F+R5 | LG+F+R5 |
| 78 | SSU ribosomal protein S10p | J | Ribosome | TIM2+F+I+G4 | LG+R2 |
| 79 | Translation elongation factor G | J | Translation factors | GTR+F+R6 | LG+F+R5 |
| 80 | SSU ribosomal protein S7p | J | Ribosome | GTR+F+I+G4 | LG+I+G4 |
| 81 | ApaG protein | P | Signaling proteins | GTR+F+R5 | WAG+F+G4 |
| 82 | RNA binding methyltransferase FtsJ like | J | Ribosome biogenesis | GTR+F+R6 | LG+F+R6 |
| 83 | 2-keto-3-deoxy-D-arabino-heptulosonate-7-phosphate synthase II | E | Phenylalanine, tyrosine and tryptophan biosynthesis<br>Quorum sensing | GTR+F+R6 | LG+F+R5 |
| 84 | NifU-like domain protein | O | Not assigned | TVM+F+R5 | LG+F+I+G4 |
| 85 | Ferric uptake regulation protein FUR | P | Transcription factors | GTR+F+R4 | LG+R3 |
| 86 | Metal-dependent hydrolase YbeY | S | Drug metabolism - other enzymes<br>Pyrimidine metabolism | TIM3+F+R5 | LG+F+I+G4 |
| 87 | Phosphate starvation-inducible protein PhoH, predicted ATPase | T | Signaling proteins | GTR+F+R5 | LG+R5 |
| 88 | tRNA-i(6)A37 methylthiotransferase | J | Transfer RNA biogenesis | GTR+F+R6 | LG+F+R5 |

| Group | Annotation | COG | KEGG | Best substitution model | |
|---|---|---|---|---|---|
| | | | | Nucleotides | Amino acids |
| 89 | A/G-specific adenine glycosylase | L | Base excision repair | GTR+F+R6 | WAG+F+R6 |
| 90 | 3-Oxoacyl-[acyl-carrier-protein] synthase, KASIII | I | Fatty acid biosynthesis | TIM3+F+R6 | LG+I+G4 |
| 91 | Hypothetical metal-binding enzyme, YcbL homolog | S | Pyruvate metabolism | GTR+F+R5 | LG+R5 |
| 92 | Aerobic cobaltochelatase CobT subunit | H | Porphyrin and chlorophyll metabolism | GTR+F+R6 | LG+F+R5 |
| 93 | ATP synthase alpha chain | C | Oxidative phosphorylation<br>Photosynthesis | GTR+F+R5 | LG+R5 |
| 94 | ATP synthase gamma chain | C | Oxidative phosphorylation<br>Photosynthesis | GTR+F+R5 | LG+F+R5 |
| 95 | ATP synthase beta chain | C | Oxidative phosphorylation<br>Photosynthesis | GTR+F+R5 | LG+R4 |
| 96 | ATP synthase epsilon chain | C | Oxidative phosphorylation<br>Photosynthesis | GTR+F+R4 | LG+R4 |
| 97 | *N*-acetylglucosamine-1-phosphate uridyltransferase | M | Amino sugar and nucleotide sugar metabolism | GTR+F+R6 | LG+F+R5 |
| 98 | Octanoate-[acyl-carrier-protein]-protein-*N*-octanoyltransferase | H | Lipoic acid metabolism | GTR+F+R6 | WAG+R5 |
| 99 | Phosphoribosylformylglycinamidine cyclo-ligase | F | Purine metabolism | TIM3+F+R6 | WAG+F+R5 |
| 100 | Nucleoside diphosphate kinase | F | Drug metabolism - other enzymes<br>Purine metabolism<br>Pyrimidine metabolism | GTR+F+R5 | WAG+I+G4 |
| 101 | Cytosol aminopeptidase PepA | E | Glutathione metabolism | GTR+F+R6 | LG+F+R6 |
| 102 | 4-hydroxythreonine-4-phosphate dehydrogenase | H | Vitamin B6 metabolism | TVM+F+R6 | LG+F+R6 |
| 103 | SSU rRNA (adenine(1518)-N(6)/adenine(1519)-N(6))-dimethyltransferase | J | Ribosome biogenesis | TPM3u+F+R6 | LG+R5 |

| Group | Annotation | COG | KEGG | Best substitution model | |
|---|---|---|---|---|---|
| | | | | Nucleotides | Amino acids |
| 104 | GTP-binding protein TypA/BipA | T | Signaling proteins | GTR+F+R5 | LG+R5 |
| 105 | Excinuclease ABC subunit B | L | Nucleotide excision repair | GTR+F+R6 | LG+F+R5 |
| 106 | N(6)-L-threonylcarbamoyladenine synthase | O | Transfer RNA biogenesis | GTR+F+R6 | LG+F+R5 |
| 107 | Glycerol-3-phosphate dehydrogenase [NAD(P)+] | C | Glycerophospholipid metabolism | TVM+F+R6 | LG+F+R5 |
| 108 | Hypothetical protein | S | Not assigned | TIM3+F+I+G4 | LG+G4 |
| 109 | Hypothetical protein | Not assigned | Not assigned | TN+F+R5 | LG+G4 |
| 110 | Ribosomal protein S12p Asp88 methylthiotransferase | J | Ribosome biogenesis | GTR+F+R6 | LG+F+R5 |
| 111 | 5'-nucleotidase SurE | F | Nicotinate and nicotinamide metabolism<br>Purine metabolism<br>Pyrimidine metabolism | GTR+F+R5 | LG+R5 |
| 112 | Seryl-tRNA synthetase | J | Aminoacyl-tRNA biosynthesis | GTR+F+R6 | LG+F+R5 |
| 113 | Aspartate carbamoyltransferase | F | Alanine, aspartate and glutamate metabolism<br>Pyrimidine metabolism | GTR+F+R5 | WAG+F+R5 |
| 114 | ATP-dependent Clp protease proteolytic subunit | O | Cell cycle | GTR+F+R5 | LG+R5 |
| 115 | ATP-dependent Clp protease ATP-binding subunit ClpX | O | Cell cycle | GTR+F+R5 | LG+R4 |
| 116 | Ribosomal large subunit pseudouridine synthase D | J | Ribosome biogenesis | TVM+F+R5 | LG+F+R6 |
| 117 | *N*-acetylmuramoyl-L-alanine amidase | M | Unclassified: metabolism | GTR+F+R6 | LG+F+R5 |
| 118 | Sulfite exporter TauE/SafE | S | Function unknown | TVM+F+R5 | LG+F+R5 |
| 119 | Valyl-tRNA synthetase | J | Aminoacyl-tRNA biosynthesis | GTR+F+R6 | LG+F+R6 |
| 120 | Ribosomal large subunit pseudouridine synthase C | J | Ribosome biogenesis | GTR+F+R6 | LG+F+R5 |
| 121 | SSU ribosomal protein S21p | J | Ribosome | GTR+F+I+G4 | LG+R2 |

| Group | Annotation | COG | KEGG | Best substitution model | |
|---|---|---|---|---|---|
| | | | | Nucleotides | Amino acids |
| 122 | Aspartyl-tRNA(Asn) amidotransferase subunit C | J | Aminoacyl-tRNA biosynthesis | GTR+F+R5 | WAG+G4 |
| 123 | Aspartyl-tRNA(Asn) amidotransferase subunit A | J | Aminoacyl-tRNA biosynthesis | GTR+F+R6 | LG+F+R6 |
| 124 | Aspartyl-tRNA(Asn) amidotransferase subunit B | J | Aminoacyl-tRNA biosynthesis | GTR+F+R5 | LG+F+R5 |
| 125 | ClpB protein | O | Chaperones and folding catalysts | GTR+F+R6 | LG+F+R5 |
| 126 | Ribonuclease HI | L | DNA replication | TIM3+F+I+G4 | LG+I+G4 |
| 127 | 4-hydroxy-3-methylbut-2-enyl diphosphate reductase | I | Terpenoid backbone biosynthesis | GTR+F+R5 | LG+R5 |
| 128 | Arginyl-tRNA synthetase | J | Aminoacyl-tRNA biosynthesis | GTR+F+R6 | LG+F+R6 |
| 129 | beta-*N*-acetylglucosaminidase | G | Amino sugar and nucleotide sugar metabolism<br>Glycosaminoglycan degradation | TIM3+F+R6 | LG+F+R6 |
| 130 | Twin-arginine translocation protein TatC | U | Protein export | TPM3u+F+R5 | LG+R6 |
| 131 | Threonine dehydratase, catabolic | E | Glycine, serine and threonine metabolism | GTR+F+R6 | LG+R6 |
| 132 | Glutamine synthetase type I | E | Alanine, aspartate and glutamate metabolism<br>Arginine biosynthesis<br>Glyoxylate and dicarboxylate metabolism<br>Nitrogen metabolism | GTR+F+R5 | LG+R4 |
| 133 | Nitrogen regulatory protein P-II | E | Two-component system | TVM+F+I+G4 | LG+I+G4 |
| 134 | Peptidase B | E | Glutathione metabolism | TIM3+F+R6 | LG+F+I+G4 |
| 135 | Methionine aminopeptidase | J | Peptidases and inhibitors | GTR+F+R5 | LG+I+G4 |
| 136 | Pterin-4-alpha-carbinolamine dehydratase | H | Folate biosynthesis | TIM3+F+R5 | WAG+G4 |
| 137 | 16S rRNA (cytosine(1402)-N(4))-methyltransferase | M | Ribosome biogenesis | TIM3+F+R5 | LG+F+I+G4 |
| 138 | Phospho-*N*-acetylmuramoyl-pentapeptide-transferase | M | Peptidoglycan biosynthesis | GTR+F+R6 | LG+F+R4 |
| 139 | UDP-*N*-acetylmuramate--L-alanine ligase | M | D-Glutamine and D-glutamate metabolism | GTR+F+R6 | LG+F+R6 |

| Group | Annotation | COG | KEGG | Best substitution model | |
|---|---|---|---|---|---|
| | | | | Nucleotides | Amino acids |
| | | | Peptidoglycan biosynthesis | | |
| 140 | UDP-*N*-acetylenolpyruvoylglucosamine reductase | M | Amino sugar and nucleotide sugar metabolism<br>Peptidoglycan biosynthesis | GTR+F+R5 | LG+R5 |
| 141 | GTP-binding protein Obg | S | Ribosome biogenesis | GTR+F+R6 | LG+F+R5 |
| 142 | Aerobic cobaltochelatase CobS subunit | H | Porphyrin and chlorophyll metabolism | GTR+F+R5 | LG+R5 |
| 143 | Putative pre-16S rRNA nuclease Yqg | L | Unclassified: metabolism | TIM3+F+R5 | LG+F+I+G4 |
| 144 | Pyridoxine 5'-phosphate synthase | H | Vitamin B6 metabolism | GTR+F+R6 | LG+R4 |
| 145 | Threonine synthase | E | Glycine, serine and threonine metabolism<br>Vitamin B6 metabolism | GTR+F+R6 | LG+F+R6 |
| 146 | LSU ribosomal protein L27p | J | Ribosome | TIM+F+I+G4 | DCMut+G4 |
| 147 | Glutamate--cysteine ligase | H | Cysteine and methionine metabolism<br>Glutathione metabolism | GTR+F+R6 | LG+R5 |
| 148 | Lysyl-tRNA synthetase (class I) | J | Aminoacyl-tRNA biosynthesis | GTR+F+R5 | LG+F+R5 |
| 149 | Energy-dependent translational throttle protein EttA | S | Transfer RNA biogenesis | GTR+F+R5 | LG+R5 |
| 150 | Transcriptional regulator, AsnC family | K | Transcription factors | GTR+F+R5 | LG+R4 |
| 151 | Biotin carboxylase of acetyl-CoA carboxylase | I | Carbon fixation pathways in prokaryotes<br>Fatty acid biosynthesis<br>Propanoate metabolism<br>Pyruvate metabolism | GTR+F+R5 | LG+R5 |
| 152 | Biotin carboxyl carrier protein of acetyl-CoA carboxylase | I | Carbon fixation pathways in prokaryotes<br>Fatty acid biosynthesis<br>Propanoate metabolism | TIM3+F+R6 | LG+F+R5 |

| Group | Annotation | COG | KEGG | Best substitution model | |
|---|---|---|---|---|---|
| | | | | Nucleotides | Amino acids |
| | | | Pyruvate metabolism | | |
| 153 | Two-component transcriptional response regulator, LuxR family | T | Two-component system | TIM+F+R5 | WAG+R4 |
| 154 | Decaprenyl diphosphate synthase | H | Terpenoid backbone biosynthesis | GTR+F+R5 | LG+F+R6 |
| 155 | DNA polymerase III subunits gamma and tau | L | DNA replication<br>Homologous recombination<br>Mismatch repair | GTR+F+R7 | LG+F+R6 |
| 156 | ATP-dependent protease La Type I | O | Cell cycle | GTR+F+R6 | LG+F+R5 |
| 157 | DNA-binding protein HU-beta | L | DNA replication | GTR+F+I+G4 | Dayhoff+G4 |
| 158 | SSU ribosomal protein S2p | J | Ribosome | TIM+F+R5 | LG+R4 |
| 159 | Translation elongation factor Ts | J | Translation factors | TIM2+F+R6 | LG+F+R4 |
| 160 | Uridine monophosphate kinase | F | Pyrimidine metabolism | GTR+F+R5 | LG+R5 |
| 161 | Ribosome recycling factor | J | Translation factors | GTR+F+R5 | LG+I+G4 |
| 162 | Undecaprenyl diphosphate synthase | I | Terpenoid backbone biosynthesis | TIM3+F+I+G4 | LG+F+R6 |
| 163 | 1-deoxy-D-xylulose 5-phosphate reductoisomerase | I | Terpenoid backbone biosynthesis | GTR+F+R6 | LG+F+R6 |
| 164 | 3-hydroxyacyl-[acyl-carrier-protein] dehydratase, FabZ form | I | Biotin metabolism<br>Fatty acid biosynthesis | GTR+F+R5 | LG+I+G4 |
| 165 | LSU ribosomal protein L31p | J | Ribosome | TIM+F+I+G4 | WAG+G4 |
| 166 | Cytochrome c-type biogenesis protein CcmC, putative heme lyase for CcmE | O | ABC transporters | TVM+F+R5 | mtZOA+F+R6 |
| 167 | Signal recognition particle receptor FtsY | U | Protein export<br>Quorum sensing | GTR+F+R5 | LG+R4 |
| 168 | Diaminopimelate epimerase | E | Lysine biosynthesis | GTR+F+R6 | LG+F+R6 |

| Group | Annotation | COG | KEGG | Best substitution model | |
|---|---|---|---|---|---|
| | | | | Nucleotides | Amino acids |
| 169 | CDP-diacylglycerol--glycerol-3-phosphate 3-phosphatidyltransferase | I | Glycerophospholipid metabolism | TVM+F+R6 | LG+F+I+G4 |
| 170 | Aspartyl-tRNA synthetase | J | Aminoacyl-tRNA biosynthesis | GTR+F+R6 | LG+R6 |
| 171 | Acyl carrier protein | I | Fatty acid biosynthesis | TIM2+F+I+G4 | JTTDCMut+G4 |
| 172 | 3-oxoacyl-[acyl-carrier-protein] synthase, KASII | I | Biotin metabolism<br>Fatty acid biosynthesis | GTR+F+R6 | LG+R5 |
| 173 | Thioredoxin reductase | O | Selenocompound metabolism | GTR+F+R6 | WAG+R5 |
| 174 | Psp operon transcriptional activator | K | Transcription factors | GTR+F+R6 | LG+F+R6 |
| 175 | 5-aminolevulinate synthase | H | Glycine, serine and threonine metabolism<br>Porphyrin and chlorophyll metabolism | GTR+F+R6 | LG+R4 |
| 176 | Acyl-phosphate:glycerol-3-phosphate O-acyltransferase PlsY | S | Glycerolipid metabolism<br>Glycerophospholipid metabolism | GTR+F+R5 | LG+F+R5 |
| 177 | DNA topoisomerase I | L | NA repair and recombination<br>DNA replication | GTR+F+R7 | LG+F+R5 |
| 178 | GTP-binding protein Era | K | Ribosome biogenesis | GTR+F+R6 | LG+R5 |
| 179 | ATP-dependent RNA helicase RhlE | L | RNA degradation | GTR+F+R6 | LG+F+R5 |
| 180 | 3-dehydroquinate synthase | E | Phenylalanine, tyrosine and tryptophan biosynthesis | GTR+F+R6 | LG+F+R6 |
| 181 | Shikimate kinase I | E | Phenylalanine, tyrosine and tryptophan biosynthesis | TPM3+F+R5 | LG+F+I+G4 |
| 182 | Site-specific tyrosine recombinase XerD | L | Chromosome and associated proteins | TVM+F+R6 | LG+F+R5 |
| 183 | Acetyl-coenzyme A carboxyl transferase alpha chain | I | Carbon fixation pathways in prokaryotes<br>Fatty acid biosynthesis | TIM3+F+R5 | LG+R5 |

| Group | Annotation | COG | KEGG | Best substitution model | |
|---|---|---|---|---|---|
| | | | | Nucleotides | Amino acids |
| | | | Propanoate metabolism<br>Pyruvate metabolism | | |
| 184 | Transcription termination protein NusA | K | Transcription machinery | GTR+F+R6 | LG+R5 |
| 185 | 23S rRNA (adenine(2503)-C(2))-methyltransferase | J | Ribosome biogenesis | GTR+F+R6 | LG+F+R5 |
| 186 | Translation initiation factor 2 | J | Translation factors | GTR+F+R6 | LG+F+R6 |
| 187 | 4-hydroxy-tetrahydrodipicolinate synthase | E | Lysine biosynthesis<br>Monobactam biosynthesis | GTR+F+R6 | LG+I+G4 |
| 188 | tmRNA-binding protein SmpB | O | Unclassified: genetic information processing | TIM+F+R5 | LG+R4 |
| 189 | RecA protein | L | Homologous recombination | GTR+F+R5 | LG+I+G4 |
| 190 | Alanyl-tRNA synthetase | J | Aminoacyl-tRNA biosynthesis | GTR+F+R6 | LG+F+R5 |
| 191 | Isocitrate dehydrogenase [NADP] | C | Carbon fixation pathways in prokaryotes<br>Citrate cycle<br>Glutathione metabolism<br>Peroxisome | GTR+F+R5 | LG+R5 |
| 192 | ATP-dependent RNA helicase Atu1833 | L | RNA degradation | GTR+F+R6 | LG+F+R6 |
| 193 | ATP-dependent DNA helicase UvrD/PcrA | L | Mismatch repair<br>Nucleotide excision repair | GTR+F+R6 | LG+F+R6 |
| 194 | Ribosomal large subunit pseudouridine synthase B | J | Ribosome biogenesis | GTR+F+R5 | LG+R5 |
| 195 | Ribosome-binding factor A | J | Ribosome biogenesis | GTR+F+R5 | LG+R4 |
| 196 | Acetyl-coenzyme A carboxyl transferase beta chain | H | Carbon fixation pathways in prokaryotes<br>Fatty acid biosynthesis<br>Propanoate metabolism | GTR+F+R6 | LG+I+G4 |

| Group | Annotation | COG | KEGG | Best substitution model | |
|---|---|---|---|---|---|
| | | | | Nucleotides | Amino acids |
| | | | Pyruvate metabolism | | |
| 197 | Tryptophan synthase alpha chain | E | Glycine, serine and threonine metabolism<br>Phenylalanine, tyrosine and tryptophan biosynthesis | GTR+F+R5 | WAG+R5 |
| 198 | Tryptophan synthase beta chain | E | Glycine, serine and threonine metabolism<br>Phenylalanine, tyrosine and tryptophan biosynthesis | GTR+F+R5 | LG+R5 |
| 199 | Phosphoribosylanthranilate isomerase | E | Phenylalanine, tyrosine and tryptophan biosynthesis | TVM+F+R5 | LG+I+G4 |
| 200 | Orotidine 5'-phosphate decarboxylase | F | Pyrimidine metabolism | GTR+F+R5 | LG+R5 |
| 201 | Adenylosuccinate lyase | F | Alanine, aspartate and glutamate metabolism<br>Purine metabolism | GTR+F+R6 | LG+F+R5 |
| 202 | Hydroxyacylglutathione hydrolase | C | Pyruvate metabolism | TIM3+F+R6 | WAG+I+R4 |
| 203 | SSU ribosomal protein S1p | J | Ribosome | TIM+F+R5 | LG+F+R5 |
| 204 | Cytidylate kinase | F | beta-Alanine metabolism<br>Pyrimidine metabolism<br>Pantothenate and CoA biosynthesis | GTR+F+R5 | WAG+F+R5 |
| 205 | 3-phosphoshikimate 1-carboxyvinyltransferase | E | Novobiocin biosynthesis<br>Phenylalanine, tyrosine and tryptophan biosynthesis | GTR+F+R6 | LG+F+R6 |
| 206 | Signal recognition particle protein Ffh | U | Protein export<br>Quorum sensing | GTR+F+R6 | LG+F+R5 |
| 207 | tRNA (guanine(37)-N(1))-methyltransferase | J | Transfer RNA biogenesis | GTR+F+R6 | LG+I+G4 |

| Group | Annotation | COG | KEGG | Best substitution model | |
|---|---|---|---|---|---|
| | | | | Nucleotides | Amino acids |
| 208 | LSU ribosomal protein L19p | J | Ribosome | GTR+F+I+G4 | LG+I+G4 |
| 209 | Aspartate-semialdehyde dehydrogenase | E | Cysteine and methionine metabolism<br>Glycine, serine and threonine metabolism<br>Lysine biosynthesis<br>Monobactam biosynthesis | GTR+F+R6 | LG+R5 |
| 210 | LSU ribosomal protein L9p | J | Ribosome | GTR+F+R5 | LG+F+I+G4 |
| 211 | SSU ribosomal protein S18p | J | Ribosome | TIM2+F+I+G4 | rtREV+R3 |
| 212 | SSU ribosomal protein S6p | J | Ribosome | TIM+F+I+G4 | LG+R3 |
| 213 | Malonyl CoA-acyl carrier protein transacylase | I | Fatty acid biosynthesis<br>Prodigiosin biosynthesis | TIM3+F+R5 | LG+F+R5 |
| 214 | 3-oxoacyl-[acyl-carrier protein] reductase | I | Biotin metabolism<br>Fatty acid biosynthesis<br>Prodigiosin biosynthesis | GTR+F+R5 | LG+R4 |
| 215 | DNA polymerase III beta subunit | L | DNA replication<br>Homologous recombination<br>Mismatch repair | GTR+F+R5 | LG+F+R5 |
| 216 | Translation elongation factor LepA | M | Legionellosis | GTR+F+R6 | LG+R5 |
| 217 | Competence lipoprotein ComL | M | Transporters | GTR+F+R5 | LG+I+G4 |
| 218 | SSU ribosomal protein S15p | J | Ribosome | GTR+F+I+G4 | WAG+I+G4 |
| 219 | DNA repair protein RecN | L | DNA repair and recombination | GTR+F+R6 | LG+F+R5 |
| 220 | DNA ligase (NAD(+)) | L | Base excision repair<br>DNA replication | GTR+F+R6 | LG+F+R6 |

| Group | Annotation | COG | KEGG | Best substitution model | |
|---|---|---|---|---|---|
| | | | | Nucleotides | Amino acids |
| | | | Mismatch repair<br>Nucleotide excision repair | | |
| 221 | Polyribonucleotide nucleotidyltransferase | J | RNA degradation | GTR+F+R6 | LG+F+R5 |
| 222 | Succinate dehydrogenase flavoprotein subunit | C | Butanoate metabolism<br>Carbon fixation pathways in prokaryotes<br>Citrate cycle<br>Oxidative phosphorylation | TIM+F+R5 | LG+R5 |
| 223 | Inosine-5'-monophosphate dehydrogenase | F | Drug metabolism - other enzymes<br>Purine metabolism | GTR+F+R6 | LG+F+R5 |
| 224 | Adenosine (5')-pentaphospho-(5'')-adenosine pyrophosphohydrolase | J | RNA degradation | TIM3+F+R5 | LG+I+R4 |
| 225 | Amidophosphoribosyltransferase | F | Alanine, aspartate and glutamate metabolism<br>Purine metabolism | GTR+F+R6 | LG+R5 |
| 226 | Lipoprotein-releasing system ATP-binding protein LolD | P | Transporters | TIM3+F+R5 | LG+I+G4 |
| 227 | DNA polymerase III alpha subunit | L | DNA replication<br>Homologous recombination<br>Mismatch repair | GTR+F+R6 | LG+F+R6 |
| 228 | Succinate dehydrogenase iron-sulfur protein | C | Butanoate metabolism<br>Carbon fixation pathways in prokaryotes<br>Citrate cycle<br>Oxidative phosphorylation | GTR+F+R6 | LG+R4 |
| 229 | GTP-binding and nucleic acid-binding protein YchF | J | Ribosome biogenesis | GTR+F+R5 | LG+F+R5 |
| 230 | Hypothetical protein | S | Chromosome and associated proteins | TVM+F+I+G4 | LG+I+G4 |
| 231 | Peptidyl-tRNA hydrolase | J | Translation factors | GTR+F+R5 | LG+R5 |

| Group | Annotation | COG | KEGG | Best substitution model | |
|---|---|---|---|---|---|
| | | | | Nucleotides | Amino acids |
| 232 | Glycyl-tRNA synthetase alpha chain | J | Aminoacyl-tRNA biosynthesis | GTR+F+R5 | WAG+R4 |
| 233 | Cysteine synthase | E | Cysteine and methionine metabolism<br>Sulfur metabolism | GTR+F+R6 | LG+R5 |
| 234 | Tyrosyl-tRNA synthetase | J | Aminoacyl-tRNA biosynthesis | GTR+F+R6 | LG+F+R6 |
| 235 | Topoisomerase IV subunit B | L | DNA repair and recombination<br>DNA replication | GTR+F+R6 | LG+R5 |
| 236 | 16S rRNA (cytidine(1402)-2'-O)-methyltransferase | S | Ribosome biogenesis | GTR+F+R5 | LG+F+I+G4 |
| 237 | Glutathione synthetase | H | Cysteine and methionine metabolism<br>Glutathione metabolism | GTR+F+R5 | LG+R5 |
| 238 | Radical SAM family enzyme | H | Porphyrin and chlorophyll metabolism | GTR+F+R6 | LG+F+R5 |
| 239 | Nucleoside 5-triphosphatase RdgB (dHAPTP, dITP, XTP-specific) | F | Purine metabolism | TIM3+F+R5 | LG+F+R4 |
| 240 | Ribonuclease PH | J | Transfer RNA biogenesis | GTR+F+R5 | LG+R4 |
| 241 | Chaperone protein DnaK | O | RNA degradation | GTR+F+R6 | LG+F+R5 |
| 242 | Chaperone protein DnaJ | O | Chaperones and folding catalysts<br>Mitochondrial biogenesis | GTR+F+R5 | LG+F+R5 |
| 243 | DNA repair protein RadA | O | DNA repair and recombination | TIM3+F+R6 | LG+F+R5 |
| 244 | Dihydrolipoamide dehydrogenase of pyruvate dehydrogenase complex | C | Citrate cycle<br>Glycine, serine and threonine metabolism<br>Glycolysis<br>Glyoxylate and dicarboxylate metabolism<br>Lysine degradation<br>Propanoate metabolism | GTR+F+R5 | LG+F+R5 |

| Group | Annotation | COG | KEGG | Best substitution model | |
|---|---|---|---|---|---|
| | | | | Nucleotides | Amino acids |
| | | | Pyruvate metabolism<br>Tryptophan metabolism<br>Valine, leucine and isoleucine degradation | | |
| 245 | Methionyl-tRNA synthetase | J | Aminoacyl-tRNA biosynthesis<br>Selenocompound metabolism | GTR+F+R6 | LG+F+R6 |
| 246 | Uncharacterized metal-dependent hydrolase YcfH | L | Unclassified: metabolism | TIM3+F+R6 | LG+F+R5 |
| 247 | Nucleoside triphosphate pyrophosphohydrolase MazG | F | Chromosome and associated proteins | GTR+F+R6 | LG+F+R6 |
| 248 | Ribosome LSU-associated GTP-binding protein HflX | S | Ribosome biogenesis | TIM3+F+R6 | LG+F+R6 |
| 249 | Nitrogen regulation protein NtrX | T | Two-component system | GTR+F+R6 | LG+F+R6 |
| 250 | tRNA-dihydrouridine synthase DusB | J | Transfer RNA biogenesis | GTR+F+R6 | LG+F+R5 |
| 251 | 2-C-methyl-D-erythritol 4-phosphate cytidylyltransferase | I | Terpenoid backbone biosynthesis | GTR+F+R7 | WAG+F+R6 |
| 252 | Nicotinamide-nucleotide amidase | S | Nicotinate and nicotinamide metabolism | TVM+F+R5 | LG+R4 |
| 253 | Lipoyl synthase | H | Lipoic acid metabolism | GTR+F+R9 | LG+R5 |
| 254 | DNA gyrase subunit A | L | DNA repair and recombination<br>DNA replication | GTR+F+R6 | LG+F+R6 |
| 255 | Methylenetetrahydrofolate--tRNA-(uracil-5-)-methyltransferase TrmFO | J | Transfer RNA biogenesis | GTR+F+R6 | LG+F+R5 |
| 256 | Glutaredoxin 3 (Grx2) | O | Chaperones and folding catalysts | GTR+F+I+G4 | WAG+I+G4 |
| 257 | 16 kDa heat shock protein A | O | Chaperones and folding catalysts | GTR+F+R5 | LG+R5 |
| 258 | CTP synthase | F | Pyrimidine metabolism | GTR+F+R6 | LG+F+R5 |
| 259 | Triosephosphate isomerase | G | Carbon fixation in photosynthetic organisms<br>Fructose and mannose metabolism<br>Glycolysis | TIM3+F+R6 | LG+F+R5 |

| Group | Annotation | COG | KEGG | Best substitution model | |
|---|---|---|---|---|---|
| | | | | Nucleotides | Amino acids |
| | | | Inositol phosphate metabolism | | |
| 260 | Anthranilate synthase, aminase component | E | Phenazine biosynthesis<br>Phenylalanine, tyrosine and tryptophan biosynthesis<br>Quorum sensing | GTR+F+R6 | WAG+F+R6 |
| 261 | Anthranilate synthase, amidotransferase component | E | Phenazine biosynthesis<br>Phenylalanine, tyrosine and tryptophan biosynthesis<br>Quorum sensing | GTR+F+R5 | WAG+I+G4 |
| 262 | Anthranilate phosphoribosyltransferase | E | Phenylalanine, tyrosine and tryptophan biosynthesis | GTR+F+R5 | LG+F+R5 |
| 263 | Indole-3-glycerol phosphate synthase | E | Phenylalanine, tyrosine and tryptophan biosynthesis | TIM3+F+R5 | LG+F+I+G4 |
| 264 | SOS-response repressor and protease LexA | K | DNA repair and recombination<br>Peptidases and inhibitors | GTR+F+R6 | LG+R5 |
| 265 | Glutamyl-tRNA synthetase | J | Aminoacyl-tRNA biosynthesis<br>Porphyrin and chlorophyll metabolism | TIM3+F+R6 | LG+f+R5 |
| 266 | Pantothenate kinase type III, CoaX-like | K | Pantothenate and CoA biosynthesis | GTR+F+R6 | LG+R5 |
| 267 | NADH-ubiquinone oxidoreductase chain N | C | Oxidative phosphorylation | TVM+F+R6 | LG+F+R5 |
| 268 | NADH-ubiquinone oxidoreductase chain M | C | Oxidative phosphorylation | GTR+F+R6 | LG+F+R5 |
| 269 | NADH-ubiquinone oxidoreductase chain L | C | Oxidative phosphorylation | GTR+F+R6 | LG+F+R5 |
| 270 | NADH-ubiquinone oxidoreductase chain K | C | Oxidative phosphorylation | TVM+F+I+G4 | mtZOA+R4 |

| Group | Annotation | COG | KEGG | Best substitution model | |
|---|---|---|---|---|---|
| | | | | Nucleotides | Amino acids |
| 271 | NADH-ubiquinone oxidoreductase chain J | C | Oxidative phosphorylation | TVM+F+R5 | LG+I+G4 |
| 272 | NADH-ubiquinone oxidoreductase chain I | C | Oxidative phosphorylation | GTR+F+R5 | LG+R3 |
| 273 | NADH-ubiquinone oxidoreductase chain H | C | Oxidative phosphorylation | TIM+F+R6 | LG+F+R5 |
| 274 | NADH-ubiquinone oxidoreductase chain G | C | Oxidative phosphorylation | GTR+F+R6 | LG+F+R5 |
| 275 | NADH-ubiquinone oxidoreductase chain F | C | Oxidative phosphorylation | GTR+F+R6 | LG+R4 |
| 276 | NADH-ubiquinone oxidoreductase chain D | C | Oxidative phosphorylation | GTR+F+R6 | LG+R4 |
| 277 | NADH-ubiquinone oxidoreductase chain C | C | Oxidative phosphorylation | GTR+F+R6 | WAG+I+G4 |
| 278 | NADH-ubiquinone oxidoreductase chain B | C | Oxidative phosphorylation | TIM+F+R5 | JTTDCMut+R5 |
| 279 | 3-ketoacyl-CoA thiolase | I | Benzoate degradation<br>Butanoate metabolism<br>Carbon fixation pathways in prokaryotes<br>Fatty acid degradation<br>Glyoxylate and dicarboxylate metabolism<br>Lysine degradation<br>Propanoate metabolism<br>Pyruvate metabolism<br>Synthesis and degradation of ketone bodies<br>Terpenoid backbone biosynthesis<br>Tryptophan metabolism<br>Valine, leucine and isoleucine degradation | GTR+F+R6 | LG+F+R5 |
| 280 | Peptide chain release factor 2 | J | Translation factors | TIM+F+R5 | LG+F+R5 |
| 281 | DNA mismatch repair protein MutL | L | Mismatch repair | TIM3+F+R6 | LG+F+R6 |
| 282 | Rod shape-determining protein MreB | D | Chromosome and associated proteins | GTR+F+R5 | LG+R4 |

| Group | Annotation | COG | KEGG | Best substitution model | |
|---|---|---|---|---|---|
| | | | | Nucleotides | Amino acids |
| | | | Cytoskeleton proteins<br>Prokaryotic defense system | | |
| 283 | Enolase | G | Glycolysis<br>Methane metabolism<br>RNA degradation | GTR+F+R6 | LG+F+R5 |
| 284 | Transcription elongation factor GreA | K | Transcription machinery | GTR+F+R5 | LG+I+G4 |
| 285 | Carbamoyl-phosphate synthase large chain | F | Alanine, aspartate and glutamate metabolism<br>Pyrimidine metabolism | GTR+F+R6 | LG+F+R6 |
| 286 | Carbamoyl-phosphate synthase small chain | F | Alanine, aspartate and glutamate metabolism<br>Pyrimidine metabolism | GTR+F+R6 | LG+R5 |
| 287 | Transamidase GatB domain protein | S | Function unknown | TIM3+F+R5 | LG+F+I+G4 |
| 288 | RNA polymerase sigma factor RpoD | K | Transcription machinery | GTR+F+R6 | LG+F+R5 |

Fig. S1. Maximum-likelihood tree based on 16S rRNA gene sequences showing the phylogenetic relationship of *Erythrobacteraceae* type strains. Bootstrap values are based on 1,000 replicates; only bootstrap values ≥70 % are shown. Bar, 0.02 substitutions per nucleotide position. Type species within the family *Erythrobacteraceae* are marked in bold. Red, orange, yellow, green, blue, indigo and purple represent the genera *Altererythrobacter*, *Croceibacterium*, *Croceicoccus*, *Erythrobacter*, *Erythromicrobium*, *Porphyrobacter* and *Qipengyuania*, respectively. *Rhodospirillum rubrum* ATCC 11170$^{T}$ was used as an outgroup.

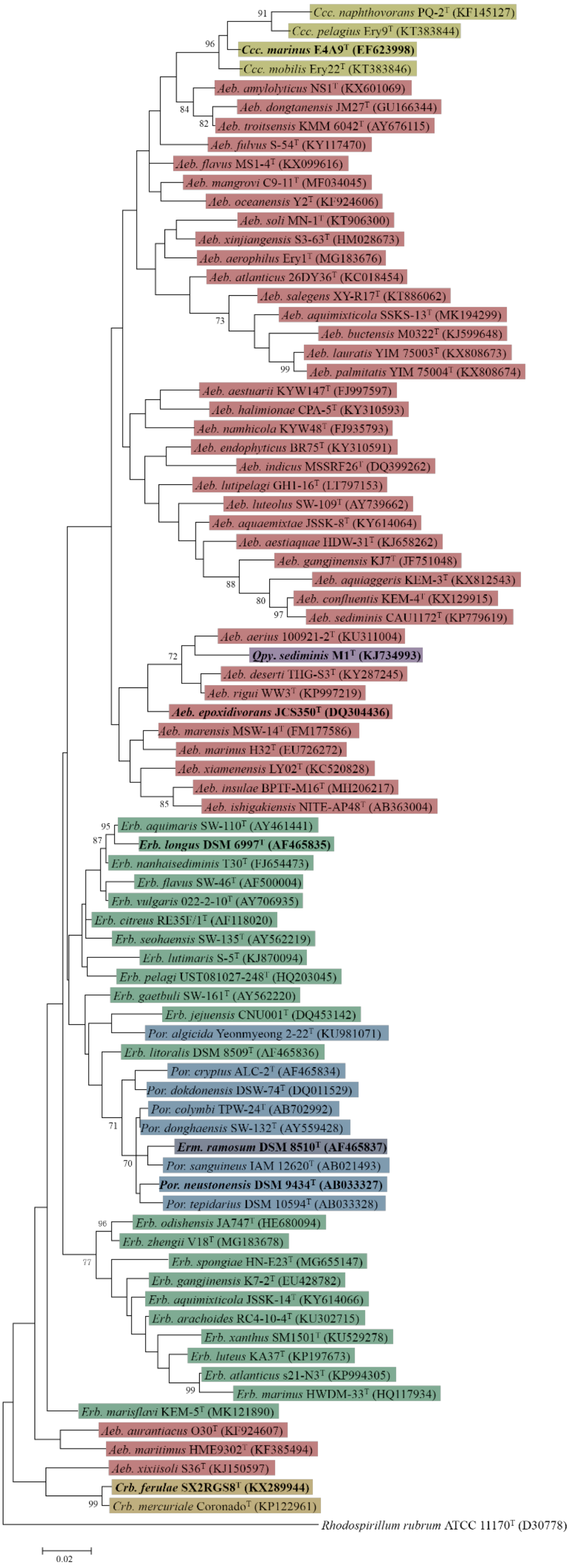

Ccc. naphthovorans PQ-2$^T$ (KF145127)
Ccc. pelagius Fry9$^T$ (KT383844)
Ccc. marinus E4A9$^T$ (EF623998)
Ccc. mobilis Fry22$^T$ (KT383846)
Aeb. amylolyticus NS1$^T$ (KX601069)
Aeb. dongtanensis JM27$^T$ (GU166344)
Aeb. troitsensis KMM 6042$^T$ (AY676115)
Aeb. fulvus S-54$^T$ (KY117470)
Aeb. flavus MS1-4$^T$ (KX099616)
Aeb. mangrovi C9-11$^T$ (MF034045)
Aeb. oceanensis Y2$^T$ (KF924606)
Aeb. soli MN-1$^T$ (KT906300)
Aeb. xinjiangensis S3-63$^T$ (HM028673)
Aeb. aerophilus Ery1$^T$ (MG183676)
Aeb. atlanticus 26DY36$^T$ (KC018454)
Aeb. salegens XY-R17$^T$ (KT886062)
Aeb. aquimixticola SSKS-13$^T$ (MK194299)
Aeb. buctensis M0322$^T$ (KJ599648)
Aeb. lauratis YIM 75003$^T$ (KX808673)
Aeb. palmitatis YIM 75004$^T$ (KX808674)
Aeb. aestuarii KYW147$^T$ (FJ997597)
Aeb. halimionae CPA-5$^T$ (KY310593)
Aeb. namhicola KYW48$^T$ (FJ935793)
Aeb. endophyticus BR75$^T$ (KY310591)
Aeb. indicus MSSRF26$^T$ (DQ399262)
Aeb. lutipelagi GH1-16$^T$ (LT797153)
Aeb. luteolus SW-109$^T$ (AY739662)
Aeb. aquaemixtae JSSK-8$^T$ (KY614064)
Aeb. aestiaquae HDW-31$^T$ (KJ658262)
Aeb. gangjinensis KJ7$^T$ (JF751048)
Aeb. aquiaggeris KEM-3$^T$ (KX812543)
Aeb. confluentis KEM-4$^T$ (KX129915)
Aeb. sediminis CAU1172$^T$ (KP779619)
Aeb. aerius 100921-2$^T$ (KU311004)
Qpy. sediminis M1$^T$ (KJ734993)
Aeb. deserti THG-S3$^T$ (KY287245)
Aeb. rigui WW3$^T$ (KP997219)
Aeb. epoxidivorans JCS350$^T$ (DQ304436)
Aeb. marensis MSW-14$^T$ (FM177586)
Aeb. marinus H32$^T$ (EU726272)
Aeb. xiamenensis LY02$^T$ (KC520828)
Aeb. insulae BPTF-M16$^T$ (MH206217)
Aeb. ishigakiensis NITE-AP48$^T$ (AB363004)
Erb. aquimaris SW-110$^T$ (AY461441)
Erb. longus DSM 6997$^T$ (AF465835)
Erb. nanhaisediminis T30$^T$ (FJ654473)
Erb. flavus SW-46$^T$ (AF500004)
Erb. vulgaris 022-2-10$^T$ (AY706935)
Erb. citreus RE35F/1$^T$ (AF118020)
Erb. seohaensis SW-135$^T$ (AY562219)
Erb. lutimaris S-5$^T$ (KJ870094)
Erb. pelagi UST081027-248$^T$ (HQ203045)
Erb. gaetbuli SW-161$^T$ (AY562220)
Erb. jejuensis CNU001$^T$ (DQ453142)
Por. algicida Yeonmyeong 2-22$^T$ (KU981071)
Erb. litoralis DSM 8509$^T$ (AF465836)
Por. cryptus ALC-2$^T$ (AF465834)
Por. dokdonensis DSW-74$^T$ (DQ011529)
Por. colymbi TPW-24$^T$ (AB702992)
Por. donghaensis SW-132$^T$ (AY559428)
Erm. ramosum DSM 8510$^T$ (AF465837)
Por. sanguineus IAM 12620$^T$ (AB021493)
Por. neustonensis DSM 9434$^T$ (AB033327)
Por. tepidarius DSM 10594$^T$ (AB033328)
Erb. odishensis JA747$^T$ (HE680094)
Erb. zhengii V18$^T$ (MG183678)
Erb. spongiae HN-E23$^T$ (MG655147)
Erb. gangjinensis K7-2$^T$ (EU428782)
Erb. aquimixticola JSSK-14$^T$ (KY614066)
Erb. arachoides RC4-10-4$^T$ (KU302715)
Erb. xanthus SM1501$^T$ (KU529278)
Erb. luteus KA37$^T$ (KP197673)
Erb. atlanticus s21-N3$^T$ (KP994305)
Erb. marinus HWDM-33$^T$ (HQ117934)
Erb. marisflavi KEM-5$^T$ (MK121890)
Aeb. aurantiacus O30$^T$ (KF924607)
Aeb. maritimus HME9302$^T$ (KF385494)
Aeb. xixiisoli S36$^T$ (KJ150597)
Crb. ferulae SX2RGS8$^T$ (KX289944)
Crb. mercuriale Coronado$^T$ (KP122961)
Rhodospirillum rubrum ATCC 11170$^T$ (D30778)
91
96
84
82
73
99
88
80
97
72
85
95
87
71
70
96
77
99
99
0.02

Fig. S2. Maximum-likelihood tree based on the concatenation of 288 single-copy OCs protein sequences showing the phylogenetic relationship of type strains belonging to the family *Erythrobacteraceae*. Bootstrap values are based on 100 replicates. Bar. 0.2 substitutions per amino acid position. *Rhodospirillum rubrum* ATCC 11170$^{T}$ was used as an outgroup (not shown).

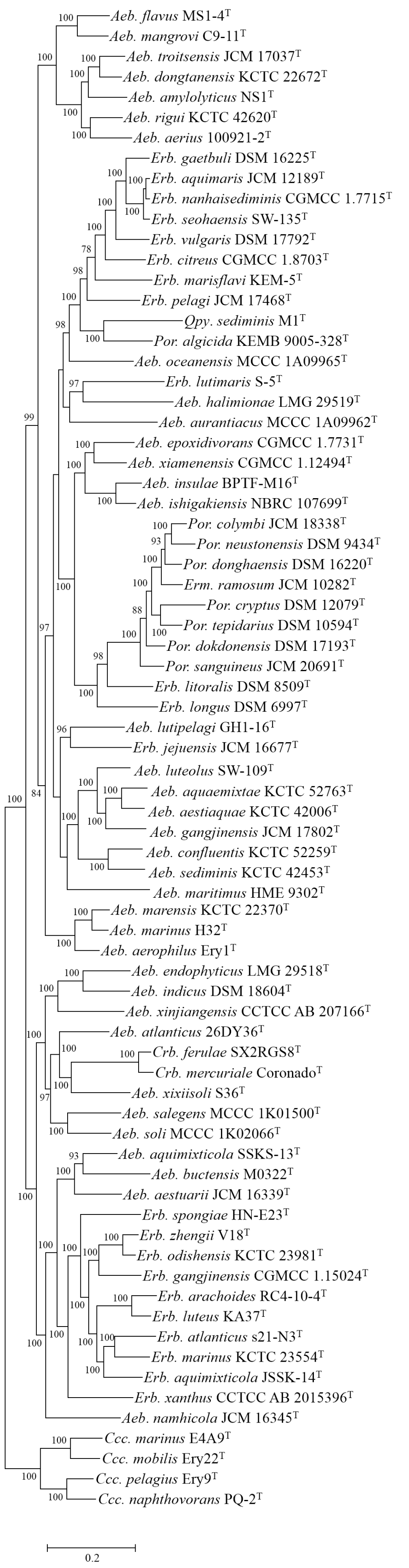
Aeb. flavus MS1-4T
Aeb. mangrovi C9-11T
Aeb. troitsensis JCM 17037T
Aeb. dongtanensis KCTC 22672T
Aeb. amylolyticus NS1T
Aeb. rigui KCTC 42620T
Aeb. aerius 100921-2T
Erb. gaetbuli DSM 16225T
Erb. aquimaris JCM 12189T
Erb. nanhaisediminis CGMCC 1.7715T
Erb. seohaensis SW-135T
Erb. vulgaris DSM 17792T
Erb. citreus CGMCC 1.8703T
Erb. marisflavi KEM-5T
Erb. pelagi JCM 17468T
Qpy. sediminis M1T
Por. algicida KEMB 9005-328T
Aeb. oceanensis MCCC 1A09965T
Erb. lutimaris S-5T
Aeb. halimionae LMG 29519T
Aeb. aurantiacus MCCC 1A09962T
Aeb. epoxidivorans CGMCC 1.7731T
Aeb. xiamenensis CGMCC 1.12494T
Aeb. insulae BPTF-M16T
Aeb. ishigakiensis NBRC 107699T
Por. colymbi JCM 18338T
Por. neustonensis DSM 9434T
Por. donghaensis DSM 16220T
Erm. ramosum JCM 10282T
Por. cryptus DSM 12079T
Por. tepidarius DSM 10594T
Por. dokdonensis DSM 17193T
Por. sanguineus JCM 20691T
Erb. litoralis DSM 8509T
Erb. longus DSM 6997T
Aeb. lutipelagi GH1-16T
Erb. jejuensis JCM 16677T
Aeb. luteolus SW-109T
Aeb. aquaemixtae KCTC 52763T
Aeb. aestiaquae KCTC 42006T
Aeb. gangjinensis JCM 17802T
Aeb. confluentis KCTC 52259T
Aeb. sediminis KCTC 42453T
Aeb. maritimus HME 9302T
Aeb. marensis KCTC 22370T
Aeb. marinus H32T
Aeb. aerophilus Ery1T
Aeb. endophyticus LMG 29518T
Aeb. indicus DSM 18604T
Aeb. xinjiangensis CCTCC AB 207166T
Aeb. atlanticus 26DY36T
Crb. ferulae SX2RGS8T
Crb. mercuriale CoronadoT
Aeb. xixiisoli S36T
Aeb. salegens MCCC 1K01500T
Aeb. soli MCCC 1K02066T
Aeb. aquimixticola SSKS-13T
Aeb. buctensis M0322T
Aeb. aestuarii JCM 16339T
Erb. spongiae HN-E23T
Erb. zhengii V18T
Erb. odishensis KCTC 23981T
Erb. gangjinensis CGMCC 1.15024T
Erb. arachoides RC4-10-4T
Erb. luteus KA37T
Erb. atlanticus s21-N3T
Erb. marinus KCTC 23554T
Erb. aquimixticola JSSK-14T
Erb. xanthus CCTCC AB 2015396T
Aeb. namhicola JCM 16345T
Ccc. marinus E4A9T
Ccc. mobilis Ery22T
Ccc. pelagius Ery9T
Ccc. naphthovorans PQ-2T
0.2

Fig. S3. Maximum-likelihood tree based on the partition of 288 single-copy OCs gene sequences showing the phylogenetic relationship of type strains belonging to the family *Erythrobacteraceae*. Bootstrap values are based on 100 replicates. Bar. 0.1 substitutions per nucleotide position. *Rhodospirillum rubrum* ATCC 11170$^T$ was used as an outgroup (not shown).

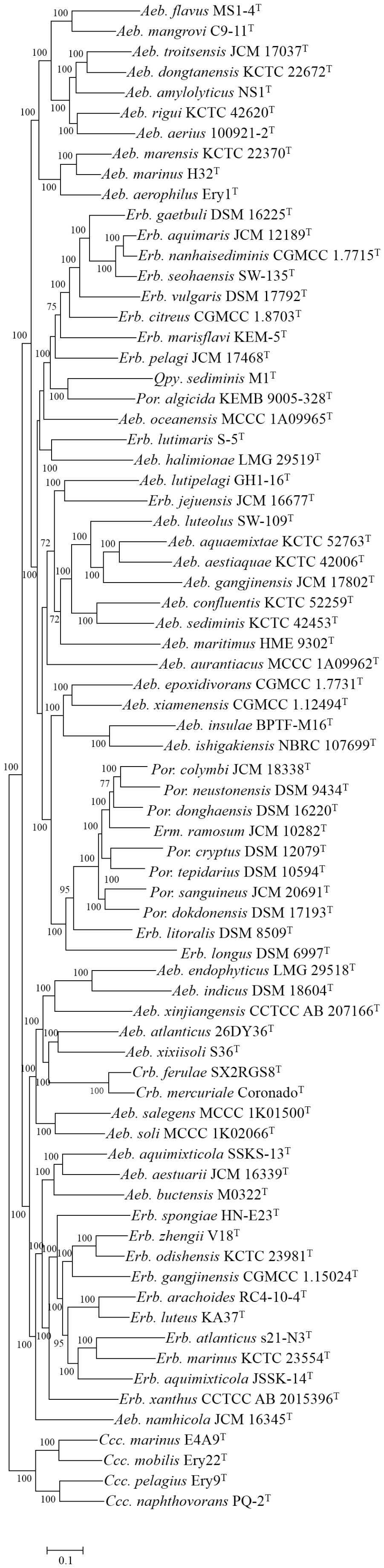
Aeb. flavus MS1-4T
Aeb. mangrovi C9-11T
Aeb. troitsensis JCM 17037T
Aeb. dongtanensis KCTC 22672T
Aeb. amylolyticus NS1T
Aeb. rigui KCTC 42620T
Aeb. aerius 100921-2T
Aeb. marensis KCTC 22370T
Aeb. marinus H32T
Aeb. aerophilus Ery1T
Erb. gaetbuli DSM 16225T
Erb. aquimaris JCM 12189T
Erb. nanhaisediminis CGMCC 1.7715T
Erb. seohaensis SW-135T
Erb. vulgaris DSM 17792T
Erb. citreus CGMCC 1.8703T
Erb. marisflavi KEM-5T
Erb. pelagi JCM 17468T
Qpy. sediminis M1T
Por. algicida KEMB 9005-328T
Aeb. oceanensis MCCC 1A09965T
Erb. lutimaris S-5T
Aeb. halimionae LMG 29519T
Aeb. lutipelagi GH1-16T
Erb. jejuensis JCM 16677T
Aeb. luteolus SW-109T
Aeb. aquaemixtae KCTC 52763T
Aeb. aestiaquae KCTC 42006T
Aeb. gangjinensis JCM 17802T
Aeb. confluentis KCTC 52259T
Aeb. sediminis KCTC 42453T
Aeb. maritimus HME 9302T
Aeb. aurantiacus MCCC 1A09962T
Aeb. epoxidivorans CGMCC 1.7731T
Aeb. xiamenensis CGMCC 1.12494T
Aeb. insulae BPTF-M16T
Aeb. ishigakiensis NBRC 107699T
Por. colymbi JCM 18338T
Por. neustonensis DSM 9434T
Por. donghaensis DSM 16220T
Erm. ramosum JCM 10282T
Por. cryptus DSM 12079T
Por. tepidarius DSM 10594T
Por. sanguineus JCM 20691T
Por. dokdonensis DSM 17193T
Erb. litoralis DSM 8509T
Erb. longus DSM 6997T
Aeb. endophyticus LMG 29518T
Aeb. indicus DSM 18604T
Aeb. xinjiangensis CCTCC AB 207166T
Aeb. atlanticus 26DY36T
Aeb. xixiisoli S36T
Crb. ferulae SX2RGS8T
Crb. mercuriale CoronadoT
Aeb. salegens MCCC 1K01500T
Aeb. soli MCCC 1K02066T
Aeb. aquimixticola SSKS-13T
Aeb. aestuarii JCM 16339T
Aeb. buctensis M0322T
Erb. spongiae HN-E23T
Erb. zhengii V18T
Erb. odishensis KCTC 23981T
Erb. gangjinensis CGMCC 1.15024T
Erb. arachoides RC4-10-4T
Erb. luteus KA37T
Erb. atlanticus s21-N3T
Erb. marinus KCTC 23554T
Erb. aquimixticola JSSK-14T
Erb. xanthus CCTCC AB 2015396T
Aeb. namhicola JCM 16345T
Ccc. marinus E4A9T
Ccc. mobilis Ery22T
Ccc. pelagius Ery9T
Ccc. naphthovorans PQ-2T
0.1

Fig. S4. Maximum-likelihood tree based on the concatenation of 288 single-copy OCs gene sequences showing the phylogenetic relationship of type strains belonging to the family *Erythrobacteraceae*. Bootstrap values are based on 100 replicates. Bar. 0.1 substitutions per nucleotide position. *Rhodospirillum rubrum* ATCC $11170^T$ was used as an outgroup (not shown).

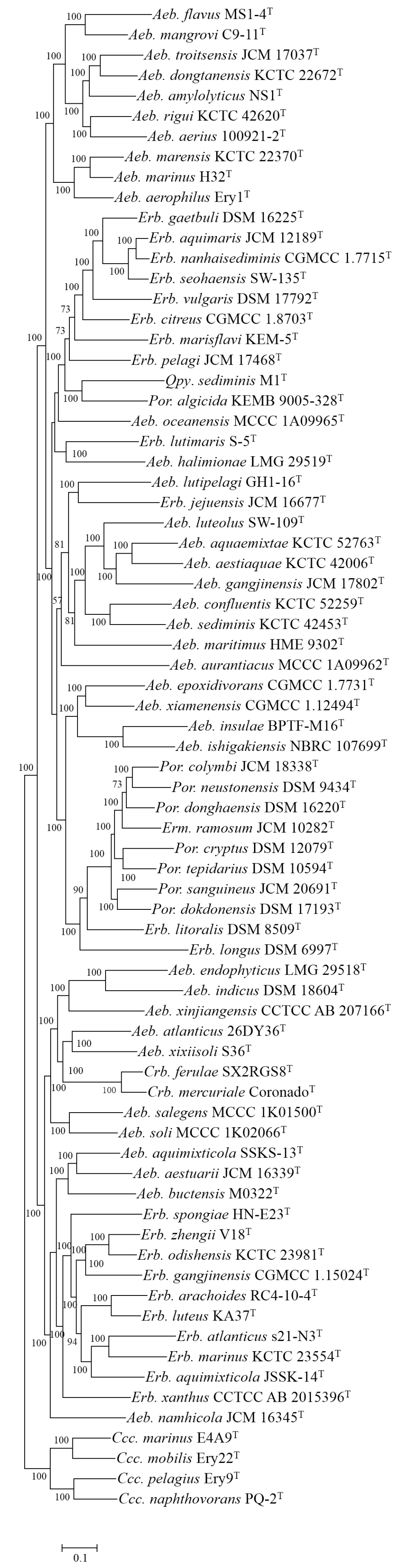
Aeb. flavus MS1-4T
Aeb. mangrovi C9-11T
Aeb. troitsensis JCM 17037T
Aeb. dongtanensis KCTC 22672T
Aeb. amylolyticus NS1T
Aeb. rigui KCTC 42620T
Aeb. aerius 100921-2T
Aeb. marensis KCTC 22370T
Aeb. marinus H32T
Aeb. aerophilus Ery1T
Erb. gaetbuli DSM 16225T
Erb. aquimaris JCM 12189T
Erb. nanhaisediminis CGMCC 1.7715T
Erb. seohaensis SW-135T
Erb. vulgaris DSM 17792T
Erb. citreus CGMCC 1.8703T
Erb. marisflavi KEM-5T
Erb. pelagi JCM 17468T
Qpy. sediminis M1T
Por. algicida KEMB 9005-328T
Aeb. oceanensis MCCC 1A09965T
Erb. lutimaris S-5T
Aeb. halimionae LMG 29519T
Aeb. lutipelagi GH1-16T
Erb. jejuensis JCM 16677T
Aeb. luteolus SW-109T
Aeb. aquaemixtae KCTC 52763T
Aeb. aestiaquae KCTC 42006T
Aeb. gangjinensis JCM 17802T
Aeb. confluentis KCTC 52259T
Aeb. sediminis KCTC 42453T
Aeb. maritimus HME 9302T
Aeb. aurantiacus MCCC 1A09962T
Aeb. epoxidivorans CGMCC 1.7731T
Aeb. xiamenensis CGMCC 1.12494T
Aeb. insulae BPTF-M16T
Aeb. ishigakiensis NBRC 107699T
Por. colymbi JCM 18338T
Por. neustonensis DSM 9434T
Por. donghaensis DSM 16220T
Erm. ramosum JCM 10282T
Por. cryptus DSM 12079T
Por. tepidarius DSM 10594T
Por. sanguineus JCM 20691T
Por. dokdonensis DSM 17193T
Erb. litoralis DSM 8509T
Erb. longus DSM 6997T
Aeb. endophyticus LMG 29518T
Aeb. indicus DSM 18604T
Aeb. xinjiangensis CCTCC AB 207166T
Aeb. atlanticus 26DY36T
Aeb. xixiisoli S36T
Crb. ferulae SX2RGS8T
Crb. mercuriale CoronadoT
Aeb. salegens MCCC 1K01500T
Aeb. soli MCCC 1K02066T
Aeb. aquimixticola SSKS-13T
Aeb. aestuarii JCM 16339T
Aeb. buctensis M0322T
Erb. spongiae HN-E23T
Erb. zhengii V18T
Erb. odishensis KCTC 23981T
Erb. gangjinensis CGMCC 1.15024T
Erb. arachoides RC4-10-4T
Erb. luteus KA37T
Erb. atlanticus s21-N3T
Erb. marinus KCTC 23554T
Erb. aquimixticola JSSK-14T
Erb. xanthus CCTCC AB 2015396T
Aeb. namhicola JCM 16345T
Ccc. marinus E4A9T
Ccc. mobilis Ery22T
Ccc. pelagius Ery9T
Ccc. naphthovorans PQ-2T
0.1

51

Fig. S5. A heatmap of ANI values for 74 type strains within the family *Erythrobacteraceae*.

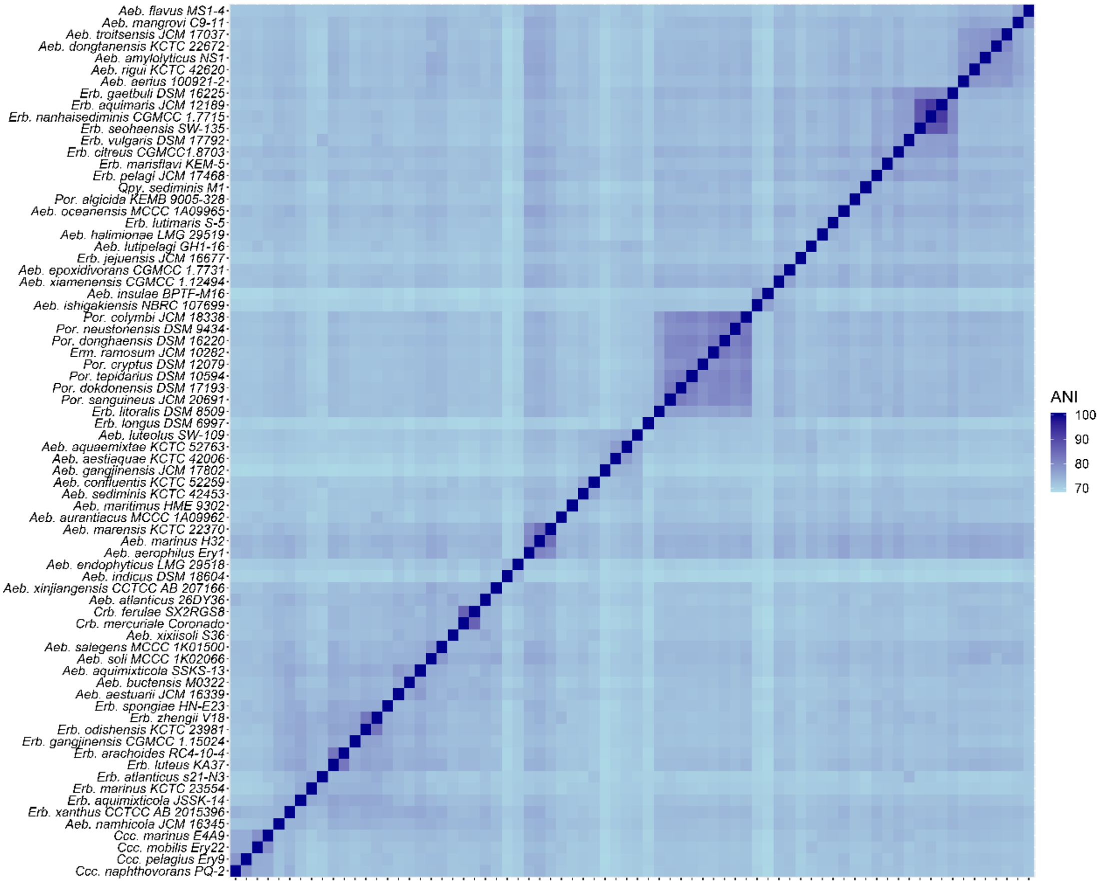

Fig. S6. A heatmap of AAI values for 74 type strains within the family *Erythrobacteraceae*.

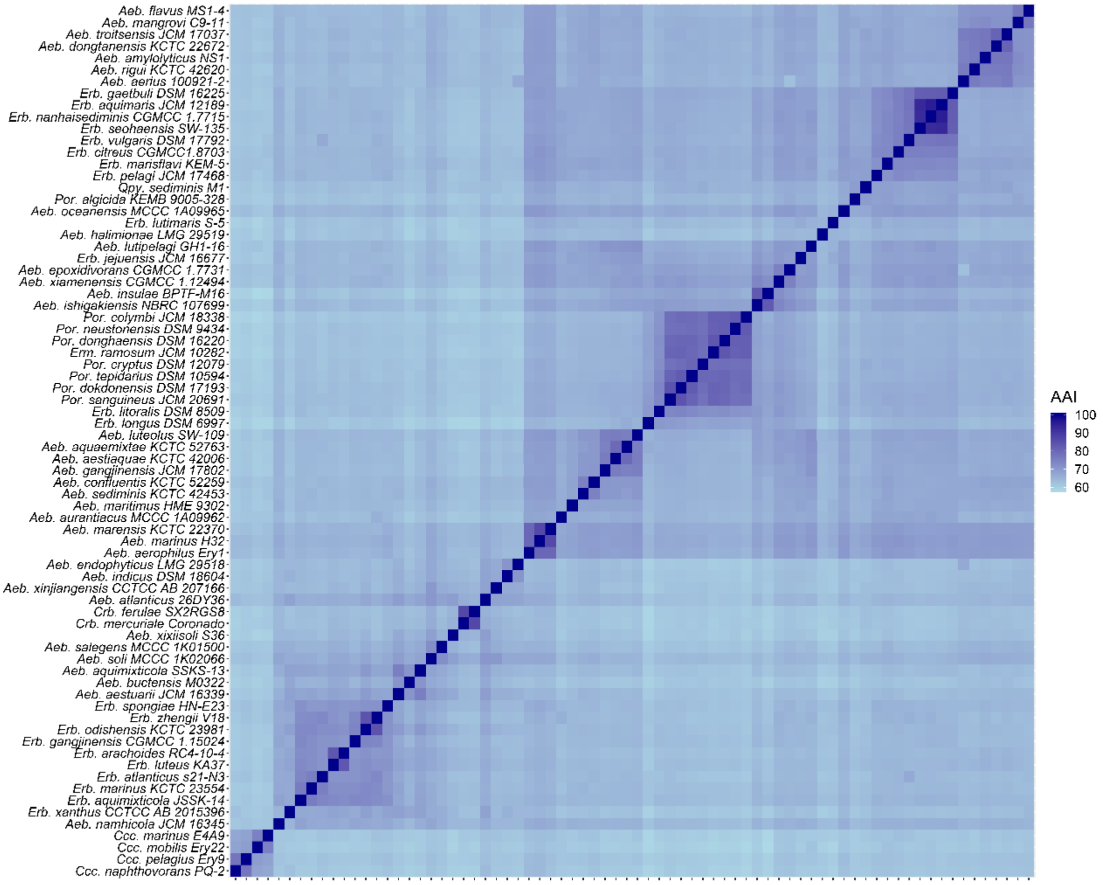

Fig. S7. A heatmap of POCP values for 74 type strains within the family *Erythrobacteraceae*.

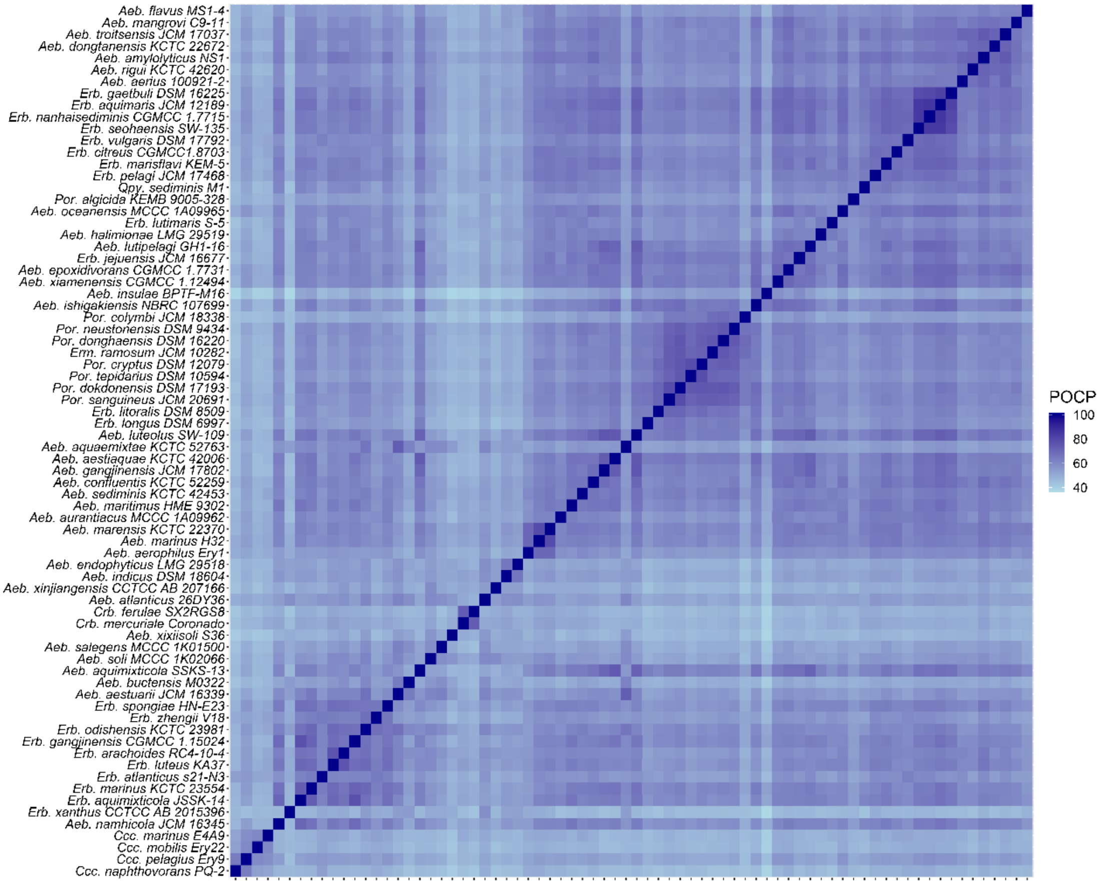

Fig. S8. Maximum-likelihood tree based on protein sequences of aerobic anoxygenic photosynthesis. Bootstrap values are based on 1,000 replicates; only bootstrap values ≥70 % are shown. Bar, 0.05 substitutions per amino acid position. *Sphingomonas sanxanigenens* NX02$^{T}$ was used as an outgroup.

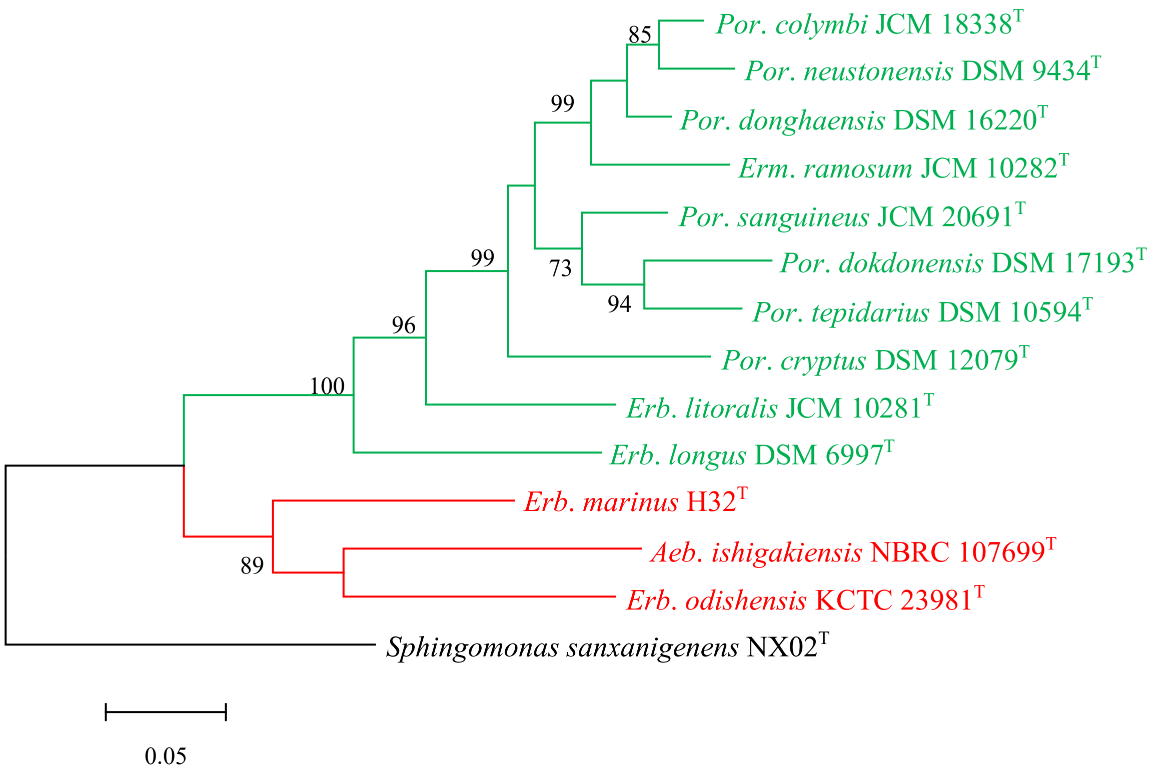

## References


1. **Xue H, Piao C-G, Guo M-W, Wang L-F, Fang W *et al.*** Description of *Altererythrobacter aerius* sp. nov., isolated from air, and emended description of the genus *Altererythrobacter*. *Int J Syst Evol Microbiol* 2016;66:4543-4548.
2. **Meng F-X, Li G, Fang C, Wu Y-H, Cheng H *et al.*** *Altererythrobacter aerophilus* sp. nov., isolated from deep-sea water of the north-west Pacific. *Int J Syst Evol Microbiol* 2019;69:1689-1695.
3. **Jung Y-T, Park S, Lee J-S, Yoon J-H**. *Altererythrobacter aestiaquae* sp. nov., isolated from seawater. *Int J Syst Evol Microbiol* 2014;64:3943-3949.
4. **Park SC, Baik KS, Choe HN, Lim C-H, Kim HJ *et al.*** *Altererythrobacter namhicola* sp. nov. and *Altererythrobacter aestuarii* sp. nov., isolated from seawater. *Int J Syst Evol Microbiol* 2011;61:709-715.
5. **Qu J-H, Ma W-W, Li H-F, Wang X-F, Lu B-B *et al.*** *Altererythrobacter amylolyticus* sp. nov., isolated from lake sediment. *Int J Syst Evol Microbiol* 2019;69:1231-1236.
6. **Park S, Jung Y-T, Choi SJ, Yoon J-H**. *Altererythrobacter aquaemixtae* sp. nov., isolated from the junction between the ocean and a freshwater spring. *Int J Syst Evol Microbiol* 2017;67:3446-3451.
7. **Jung Y-T, Park S, Lee J-S, Yoon J-H**. *Altererythrobacter aquiaggeris* sp. nov., isolated from water of an estuary bank. *Int J Syst Evol Microbiol* 2017;67:3410-3416.
8. **Park S, Park J-M, Yoon J-H**. *Altererythrobacter aquimixticola* sp. nov., isolated from sediment sampled at the junction between the ocean and a freshwater spring. *Int J Syst Evol Microbiol* 2019;69:2408-2414.
9. **Wu Y-H, Xu L, Meng F-X, Zhang D-S, Wang C-S *et al.*** *Altererythrobacter atlanticus* sp. nov., isolated from deep-sea sediment. *Int J Syst Evol Microbiol* 2014;64:116-121.
10. **Zhang G, Yang Y, Wang L**. *Altererythrobacter aurantiacus* sp. nov., isolated from deep-sea sediment. *Antonie van Leeuwenhoek* 2016;109:1245-1251.
11. **Zhang W, Yuan X, Feng Q, Zhang R, Zhao X *et al.*** *Altererythrobacter buctense* sp. nov., isolated from mudstone core. *Antonie van Leeuwenhoek* 2016;109:793-799.
12. **Park S, Jung Y-T, Park J-M, Yoon J-H**. *Altererythrobacter confluentis* sp. nov., isolated from water of an estuary environment. *Int J Syst Evol Microbiol* 2016;66:4002-4008.
13. **Yan Z-F, Lin P, Won K-H, Yang J-E, Li C-T *et al.*** *Altererythrobacter deserti* sp. nov., isolated from desert soil. *Int J Syst Evol Microbiol* 2017;67:3806-3811.
14. **Fan Z-Y, Xiao Y-P, Hui W, Tian G-R, Lee J-S *et al.*** *Altererythrobacter dongtanensis* sp. nov., isolated from a tidal flat. *Int J Syst Evol Microbiol* 2011;61:2035-2039.
15. **Fidalgo C, Rocha J, Martins R, Proença DN, Morais PV *et al.*** *Altererythrobacter halimionae* sp. nov. and *Altererythrobacter endophyticus* sp.

nov., two endophytes from the salt marsh plant *Halimione portulacoides*. *Int J Syst Evol Microbiol* 2017;67:3057-3062.

16. **Kwon KK, Woo J-H, Yang S-H, Kang J-H, Kang SG *et al.*** *Altererythrobacter epoxidivorans* gen. nov., sp. nov., an epoxide hydrolase-active, mesophilic marine bacterium isolated from cold-seep sediment, and reclassification of *Erythrobacter luteolus* Yoon *et al*. 2005 as *Altererythrobacter luteolus* comb. nov. *Int J Syst Evol Microbiol* 2007;57:2207-2211.
17. **Ma H, Ren H, Huang L, Luo Y**. *Altererythrobacter flavus* sp. nov., isolated from mangrove sediment. *Int J Syst Evol Microbiol* 2018;68:2265-2270.
18. **Dahal RH, Kim J**. *Altererythrobacter fulvus* sp. nov., a novel alkalitolerant alphaproteobacterium isolated from forest soil. *Int J Syst Evol Microbiol* 2018;6:1502-1508.
19. **Jeong SH, Jin HM, Lee HJ, Jeon CO**. *Altererythrobacter gangjinensis* sp. nov., a marine bacterium isolated from a tidal flat. *Int J Syst Evol Microbiol* 2013;63:971-976.
20. **Kumar NR, Nair S, Langer S, Busse HJ, Kämpfer P**. *Altererythrobacter indicus* sp. nov., isolated from wild rice (*Porteresia coarctata* Tateoka). *Int J Syst Evol Microbiol* 2008;58:839-844.
21. **Park S, Park J-M, Oh T-K, Yoon J-H**. *Altererythrobacter insulae* sp. nov., a lipolytic bacterium isolated from a tidal flat. *Int J Syst Evol Microbiol* 2019;69:1009-1015.
22. **Matsumoto M, Iwama D, Arakaki A, Tanaka A, Tanaka T *et al.*** *Altererythrobacter ishigakiensis* sp. nov., an astaxanthin-producing bacterium isolated from a marine sediment. *Int J Syst Evol Microbiol* 2011;61:2956-2961.
23. **Yuan C-G, Chen X, Jiang Z, Chen W, Liu L *et al.*** *Altererythrobacter lauratis* sp. nov. and *Altererythrobacter palmitatis* sp. nov., isolated from a Tibetan hot spring. *Antonie van Leeuwenhoek* 2017;110:1077-1086.
24. **Yoon J-H, Kang KH, Yeo S-H, Oh T-K**. *Erythrobacter luteolus* sp. nov., isolated from a tidal flat of the Yellow Sea in Korea. *Int J Syst Evol Microbiol* 2005;55:1167-1170.
25. **Lee SD**. *Altererythrobacter lutipelagi* sp. nov., isolated from a tidal mudflat, and emended description of the genus *Altererythrobacter*. *Int J Syst Evol Microbiol* 2019;69:1980-1985.
26. **Liao H, Li Y, Zhang M, Lin X, Lai Q *et al.*** *Altererythrobacter mangrovi* sp. nov., isolated from mangrove sediment. *Int J Syst Evol Microbiol* 2017;67:4851-4856.
27. **Seo SH, Lee SD**. *Altererythrobacter marensis* sp. nov., isolated from seawater. *Int J Syst Evol Microbiol* 2010;60:307-311.
28. **Lai Q, Yuan J, Shao Z**. *Altererythrobacter marinus* sp. nov., isolated from deep seawater. *Int J Syst Evol Microbiol* 2009;59:2973-2976.
29. **Kang H, Kim H, Joh K**. *Altererythrobacter maritimus* sp. nov., isolated from seawater. *Int J Syst Evol Microbiol* 2019;69:1566-1572.

30. **Yang Y, Zhang G, Sun Z, Cheung MK, Huang C**. *Altererythrobacter oceanensis* sp. nov., isolated from the Western Pacific. *Antonie van Leeuwenhoek* 2014;106:1191-1198.
31. **Kang JW, Kim MS, Lee JH, Baik KS, Seong CN**. *Altererythrobacter rigui* sp. nov., isolated from wetland freshwater. *Int J Syst Evol Microbiol* 2016;66:2491-2496.
32. **Liang X, Lin H, Wang K, Liao Y, Lai Q *et al.*** *Altererythrobacter salegens* sp. nov., a slightly halophilic bacterium isolated from surface sediment. *Int J Syst Evol Microbiol* 2017;67:909-913.
33. **Kim J-H, Yoon J-H, Kim W**. *Altererythrobacter sediminis* sp. nov., isolated from lagoon sediments. *Int J Syst Evol Microbiol* 2016;66:5424-5429.
34. **Zhao Q, Li H-R, Han Q-Q, He A-L, Nie C-Y *et al.*** *Altererythrobacter soli* sp. nov., isolated from desert sand. *Int J Syst Evol Microbiol* 2017;67:454-459.
35. **Nedashkovskaya OI, Cho S-H, Joung Y, Joh K, Kim MN *et al.*** *Altererythrobacter troitsensis* sp. nov., isolated from the sea urchin *Strongylocentrotus intermedius*. *Int J Syst Evol Microbiol* 2013;63:93-97.
36. **Lei X, Li Y, Chen Z, Zheng W, Lai Q *et al.*** *Altererythrobacter xiamenensis* sp. nov., an algicidal bacterium isolated from red tide seawater. *Int J Syst Evol Microbiol* 2014;64:631-637.
37. **Xue X, Zhang K, Cai F, Dai J, Wang Y *et al.*** *Altererythrobacter xinjiangensis* sp. nov., isolated from desert sand, and emended description of the genus *Altererythrobacter*. *Int J Syst Evol Microbiol* 2012;62:28-32.
38. **Yuan N, Zeng Y, Feng H, Yu Z, Huang Y**. *Altererythrobacter xixiisoli* sp. nov., isolated from wetland soil. *Int J Syst Evol Microbiol* 2017;67:3655-3659.
39. **Liu Y-H, Fang B-Z, Dong Z-Y, Li L, Mohamad OAA *et al.*** *Croceibacterium* gen. nov., with description of *Croceibacterium ferulae* sp. nov., an endophytic bacterium isolated from *Ferula sinkiangensis* K. M. Shen and reclassification of *Porphyrobacter mercurialis* as *Croceibacterium mercuriale* comb. nov. *Int J Syst Evol Microbiol* 2019;69:2547-2554.
40. **Coil DA, Flanagan JC, Stump A, Alexiev A, Lang JM *et al.*** *Porphyrobacter mercurialis* sp nov., isolated from a stadium seat and emended description of the genus *Porphyrobacter*. *PeerJ* 2015;3.
41. **Xu X-W, Wu Y-H, Wang C-S, Wang X-G, Oren A *et al.*** *Croceicoccus marinus* gen. nov., sp nov., a yellow-pigmented bacterium from deep-sea sediment, and emended description of the family *Erythrobacteraceae*. *Int J Syst Evol Microbiol* 2009;59:2247-2253.
42. **Wu Y-H, Li G-Y, Jian S-L, Cheng H, Huo Y-Y *et al.*** *Croceicoccus pelagius* sp. nov. and *Croceicoccus mobilis* sp. nov., isolated from marine environments. *Int J Syst Evol Microbiol* 2016;61:4506-4511.
43. **Huang Y, Zeng Y, Feng H, Wu Y, Xu X**. *Croceicoccus naphthovorans* sp. nov., a polycyclic aromatic hydrocarbons-degrading and acylhomoserine-lactone-

producing bacterium isolated from marine biofilm, and emended description of the genus *Croceicoccus*. *Int J Syst Evol Microbiol* 2015;65:1531-1536.

44. **Yoon J-H, Kang K-H, Oh T-K, Park Y-H**. *Erythrobacter aquimaris* sp. nov., isolated from sea water of a tidal flat of the Yellow Sea in Korea. *Int J Syst Evol Microbiol* 2004;54:1981-1985.
45. **Park S, Jung Y-T, Choi SJ, Yoon J-H**. *Erythrobacter aquimixticola* sp. nov., isolated from the junction between the ocean and a freshwater spring. *Int J Syst Evol Microbiol* 2017;67:2964-2969.
46. **Xing T, Liu Y, Wang N, Xu B, Liu K *et al.*** *Erythrobacter arachoides* sp. nov., isolated from ice core. *Int J Syst Evol Microbiol* 2017;67:4235-4239.
47. **Zhuang L, Liu Y, Wang L, Wang W, Shao Z**. *Erythrobacter atlanticus* sp. nov., a bacterium from ocean sediment able to degrade polycyclic aromatic hydrocarbons. *Int J Syst Evol Microbiol* 2015;65:3714-3719.
48. **Denner EB, Vybiral D, Koblízek M, Kämpfer P, Busse HJ *et al.*** *Erythrobacter citreus* sp. nov., a yellow-pigmented bacterium that lacks bacteriochlorophyll *a*, isolated from the western Mediterranean Sea. *Int J Syst Evol Microbiol* 2002;52:1655-1661.
49. **Yoon J-H, Kim H, Kim I-G, Kang KH, Park Y-H**. *Erythrobacter flavus* sp. nov., a slight halophile from the East Sea in Korea. *Int J Syst Evol Microbiol* 2003;53:1169-1174.
50. **Yoon J-H, Oh T-K, Park Y-H**. *Erythrobacter seohaensis* sp. nov. and *Erythrobacter gaetbuli* sp. nov., isolated from a tidal flat of the Yellow Sea in Korea. *Int J Syst Evol Microbiol* 2005;55:71-75.
51. **Lee YS, Lee D-H, Kahng H-Y, Kim EM, Jung JS**. *Erythrobacter gangjinensis* sp. nov., a marine bacterium isolated from seawater. *Int J Syst Evol Microbiol* 2010;60:1413-1417.
52. **Yoon B-J, Lee D-H, Oh D-C**. *Erythrobacter jejuensis* sp. nov., isolated from seawater. *Int J Syst Evol Microbiol* 2013;63:1421-1426.
53. **Yurkov V, Stackebrandt E, Holmes A, Fuerst JA, Hugenholtz P *et al.*** Phylogenetic positions of novel aerobic, bacteriochlorophyll *a*-containing bacteria and description of *Roseococcus thiosulfatophilus* gen. nov., sp. nov., *Erythromicrobium ramosum* gen. nov., sp. nov., and *Erythrobacter litoralis* sp. nov. *Int J Syst Bacteriol* 1994;44:427-434.
54. **Shiba T, Simidu U**. *Erythrobacter longus* gen. nov., sp. nov., an aerobic bacterium which contains bacteriochlorophyll *a*. *Int J Syst Bacteriol* 1982;32:211-217.
55. **Lei X, Zhang H, Chen Y, Li Y, Chen Z *et al.*** *Erythrobacter luteus* sp. nov., isolated from mangrove sediment. *Int J Syst Evol Microbiol* 2015;65:2472-2478.
56. **Jung Y-T, Park S, Lee J-S, Yoon J-H**. *Erythrobacter lutimaris* sp. nov., isolated from a tidal flat sediment. *Int J Syst Evol Microbiol* 2014;64:4184-4190.
57. **Jung Y-T, Park S, Oh T-K, Yoon J-H**. *Erythrobacter marinus* sp. nov., isolated from seawater. *Int J Syst Evol Microbiol* 2012;62:2050-2055.

58. **Park S, Won SM, Yoon J-H**. *Erythrobacter marisflavi* sp. nov., isolated from isolated from estuary water. *Int J Syst Evol Microbiol* 2019;69:2696-2702.
59. **Xu M, Xin Y, Yu Y, Zhang J, Zhou Y *et al.*** *Erythrobacter nanhaisediminis* sp. nov., isolated from marine sediment of the South China Sea. *Int J Syst Evol Microbiol* 2010;60:2215-2220.
60. **Subhash Y, Tushar L, Sasikala C, Ramana CV**. *Erythrobacter odishensis* sp. nov. and *Pontibacter odishensis* sp. nov. isolated from dry soil of a solar saltern. *Int J Syst Evol Microbiol* 2013;63:4524-4532.
61. **Wu H-X, Lai PY, Lee OO, Zhou X-J, Miao L *et al.*** *Erythrobacter pelagi* sp nov., a member of the family *Erythrobacteraceae* isolated from the Red Sea. *Int J Syst Evol Microbiol* 2012;62:1348-1353.
62. **Zhuang L, Lin B, Xu L, Li G, Wu C-J *et al.*** *Erythrobacter spongiae* sp. nov., isolated from marine sponge. *Int J Syst Evol Microbiol* 2019;69:1111-1116.
63. **Ivanova EP, Bowman JP, Lysenko AM, Zhukova NV, Gorshkova NM *et al.*** *Erythrobacter vulgaris* sp. nov., a novel organism isolated from the marine invertebrates. *Syst Appl Microbiol* 2005;28:123-130.
64. **Li D-D, Zhang Y-Q, Peng M, Wang N, Wang X-J *et al.*** *Erythrobacter xanthus* sp. nov., isolated from surface seawater of the South China Sea. *Int J Syst Evol Microbiol* 2017;67:2459-2464.
65. **Fang C, Wu Y-H, Sun C, Wang H, Cheng H *et al.*** *Erythrobacter zhengii* sp. nov., a bacterium isolated from deep-sea sediment. *Int J Syst Evol Microbiol* 2019;69:241-248.
66. **Kristyanto S, Lee SD, Kim J**. *Porphyrobacter algicida* sp. nov., an algalytic bacterium isolated from seawater. *Int J Syst Evol Microbiol* 2017;67:4526-4533.
67. **Furuhata K, Edagawa A, Miyamoto H, Kawakami Y, Fukuyama M**. *Porphyrobacter colymbi* sp. nov. isolated from swimming pool water in Tokyo, Japan. *J Gen Appl Microbiol* 2013;59:245-250.
68. **Rainey FA, Silva J, Nobre MF, Silva MT, da Costa MS**. *Porphyrobacter cryptus* sp. nov., a novel slightly thermophilic, aerobic, bacteriochlorophyll *a*-containing species. *Int J Syst Evol Microbiol* 2003;53:35-41.
69. **Yoon J-H, Kang S-J, Lee M-H, Oh HW, Oh T-K**. *Porphyrobacter dokdonensis* sp. nov., isolated from sea water. *Int J Syst Evol Microbiol* 2006;56:1079-1083.
70. **Yoon J-H, Lee M-H, Oh T-K**. *Porphyrobacter donghaensis* sp. nov., isolated from sea water of the East Sea in Korea. *Int J Syst Evol Microbiol* 2004;54:2231-2235.
71. **Fuerst JA, Hawkins JA, Holmes A, Sly LI, Moore CJ *et al.*** *Porphyrobacter neustonensis* gen. nov., sp. nov., an aerobic bacteriochlorophyll-synthesizing budding bacterium from fresh water. *Int J Syst Bacteriol*1993;43:125-134.
72. **Hiraishi A, Yonemitsu Y, Matsushita M, Shin YK, Kuraishi H *et al.*** Characterization of *Porphyrobacter sanguineus* sp. nov., an aerobic bacteriochlorophyll-containing bacterium capable of degrading biphenyl and dibenzofuran. *Arch Microbiol* 2002;178:45-52.

73. **Hanada S, Kawase Y, Hiraishi A, Takaichi S, Matsuura K *et al.*** *Porphyrobacter tepidarius* sp. nov., a moderately thermophilic aerobic photosynthetic bacterium isolated from a hot spring. *Int J Syst Bacteriol* 1997;47:408-413.
74. **Feng X-M, Mo Y-X, Han L, Nogi Y, Zhu Y-H *et al.*** *Qipengyuania sediminis* gen. nov., sp. nov., a member of the family *Erythrobacteraceae* isolated from subterrestrial sediment. *Int J Syst Evol Microbiol* 2015;65:3658-3665.
